# The molecular epidemiology of multiple zoonotic origins of SARS-CoV-2

**DOI:** 10.1126/science.abp8337

**Published:** 2022-07-26

**Authors:** Jonathan E. Pekar, Andrew Magee, Edyth Parker, Niema Moshiri, Katherine Izhikevich, Jennifer L. Havens, Karthik Gangavarapu, Lorena Mariana Malpica Serrano, Alexander Crits-Christoph, Nathaniel L. Matteson, Mark Zeller, Joshua I. Levy, Jade C. Wang, Scott Hughes, Jungmin Lee, Heedo Park, Man-Seong Park, Katherine Zi Yan Ching, Raymond Tzer Pin Lin, Mohd Noor Mat Isa, Yusuf Muhammad Noor, Tetyana I. Vasylyeva, Robert F. Garry, Edward C. Holmes, Andrew Rambaut, Marc A. Suchard, Kristian G. Andersen, Michael Worobey, Joel O. Wertheim

**Affiliations:** ^1^ Bioinformatics and Systems Biology Graduate Program, University of California San Diego, La Jolla, CA 92093, USA.; ^2^ Department of Biomedical Informatics, University of California San Diego, La Jolla, CA 92093, USA.; ^3^ Department of Human Genetics, David Geffen School of Medicine, University of California Los Angeles, Los Angeles, CA 90095, USA.; ^4^ Department of Immunology and Microbiology, The Scripps Research Institute, La Jolla, CA 92037, USA.; ^5^ Department of Computer Science and Engineering, University of California San Diego, La Jolla, CA 92093, USA.; ^6^ Department of Mathematics, University of California San Diego, La Jolla, CA 92093, USA.; ^7^ Department of Ecology and Evolutionary Biology, University of Arizona, Tucson, AZ 85721, USA.; ^8^ W. Harry Feinstone Department of Molecular Microbiology and Immunology, Johns Hopkins Bloomberg School of Public Health, Baltimore, Maryland 21205, USA.; ^9^ New York City Public Health Laboratory, New York City Department of Health and Mental Hygiene, New York, NY 11101, USA.; ^10^ Department of Microbiology, Institute for Viral Diseases, Biosafety Center, College of Medicine, Korea University, Seoul, South Korea.; ^11^ BK21 Graduate Program, Department of Biomedical Sciences, Korea University College of Medicine, Seoul, 02841, Republic of Korea.; ^12^ National Public Health Laboratory, National Centre for Infectious Diseases, Singapore.; ^13^ Malaysia Genome and Vaccine Institute, Jalan Bangi, 43000 Kajang, Selangor, Malaysia.; ^14^ Department of Medicine, University of California San Diego, La Jolla, CA 92093, USA.; ^15^ Tulane University, School of Medicine, Department of Microbiology and Immunology, New Orleans, LA 70112, USA.; ^16^ Zalgen Labs, LCC, Frederick, MD 21703 USA.; ^17^ Global Virus Network (GVN), Baltimore, MD 21201, USA.; ^18^ Sydney Institute for Infectious Diseases, School of Life and Environmental Sciences and School of Medical Sciences, The University of Sydney, Sydney, NSW 2006, Australia.; ^19^ Institute of Evolutionary Biology, University of Edinburgh, King's Buildings, Edinburgh, EH9 3FL, UK.; ^20^ Department of Biomathematics, David Geffen School of Medicine, University of California Los Angeles, Los Angeles, CA 90095, USA.; ^21^ Department of Biostatistics, Fielding School of Public Health, University of California Los Angeles, Los Angeles, CA 90095, USA.; ^22^ Scripps Research Translational Institute, La Jolla, CA 92037, USA.

## Abstract

Understanding the circumstances that lead to pandemics is important for their prevention. Here, we analyze the genomic diversity of severe acute respiratory syndrome coronavirus 2 (SARS-CoV-2) early in the coronavirus disease 2019 (COVID-19) pandemic. We show that SARS-CoV-2 genomic diversity before February 2020 likely comprised only two distinct viral lineages, denoted A and B. Phylodynamic rooting methods, coupled with epidemic simulations, reveal that these lineages were the result of at least two separate cross-species transmission events into humans. The first zoonotic transmission likely involved lineage B viruses around 18 November 2019 (23 October–8 December), while the separate introduction of lineage A likely occurred within weeks of this event. These findings indicate that it is unlikely that SARS-CoV-2 circulated widely in humans prior to November 2019 and define the narrow window between when SARS-CoV-2 first jumped into humans and when the first cases of COVID-19 were reported. As with other coronaviruses, SARS-CoV-2 emergence likely resulted from multiple zoonotic events.

Severe acute respiratory syndrome coronavirus 2 (SARS-CoV-2) is responsible for the coronavirus disease 19 (COVID-19) pandemic that caused more than 5 million confirmed deaths in the two years following its detection at the Huanan Seafood Wholesale Market (hereafter the ‘Huanan market’) in December 2019 in Wuhan, China (*1*–*3*). As the original outbreak spread to other countries, the diversity of SARS-CoV-2 quickly increased and led to the emergence of multiple variants of concern, but the beginning of the pandemic was marked by two major lineages denoted ‘A’ and ‘B’ (*4*).

Lineage B has been the most common throughout the pandemic and includes all eleven sequenced genomes from humans directly associated with the Huanan market, including the earliest sampled genome, Wuhan/IPBCAMS-WH-01/2019, and the reference genome, Wuhan/Hu-1/2019 (hereafter ‘Hu-1’) (*5*), sampled on 24 and 26 December 2019, respectively. The earliest lineage A viruses, Wuhan/IME-WH01/2019 and Wuhan/WH04/2020, were sampled on 30 December 2019 and 5 January 2020, respectively (*6*). Lineage A differs from lineage B by two nucleotide substitutions, C8782T and T28144C, which are also found in related coronaviruses from *Rhinolophus* bats (*4*), the presumed host reservoir (*7*). Lineage B viruses have a ‘C/T’ pattern at these key sites (C8782, T28144), whereas lineage A viruses have a ‘T/C’ pattern (C8782T, T28144C). The earliest lineage A genomes from humans lack a direct epidemiological connection to the Huanan market, but were sampled from individuals who lived or had recently stayed close to the market (*8*). It has been hypothesized that lineages A and B emerged separately (*9*), but ‘C/C’ and ‘T/T’ genomes intermediate to lineages A and B present a challenge to that hypothesis, as their existence suggests within-human evolution of one lineage toward the other via a transitional form.

Questions about these lineages remain: if lineage B viruses are more distantly related to sarbecoviruses from *Rhinolophus* bats, (i) why were lineage B viruses detected earlier than lineage A viruses and (ii) why did lineage B predominate early in the pandemic?

Answering these questions requires determining the ancestral haplotype, the genomic sequence characteristics of the most recent common ancestor (MRCA) at the root of the SARS-CoV-2 phylogeny. In this study, we combined genomic and epidemiological data from early in the COVID-19 pandemic with phylodynamic models and epidemic simulations. We eliminated many of the haplotypes previously suggested as the MRCA of SARS-CoV-2 and show that the pandemic most likely began with at least two separate zoonotic transmissions starting in November 2019.

## Results

### Erroneous assignment of haplotypes intermediate to lineages A and B

There are 787 near-full length genomes available from lineages A and B sampled by 14 February 2020 (data S1 and S2). However, there are also 20 genomes of intermediate haplotypes from this period containing either T28144C or C8782T but not both mutations: C/C or T/T, respectively.

We identified numerous instances of C/C and T/T genomes sharing rare mutations with lineage A or lineage B viruses, often sequenced in the same laboratory, indicating these intermediate genomes are likely artifacts of contamination or bioinformatics (*10*), similar to findings from our analysis of the emergence of SARS-CoV-2 in North America (*11*) (fig. S1 and supplementary text). We confirmed that a C/C genome from South Korea sharing three such mutations had low sequencing depth at position 28144 (≤10x), a T/T genome sampled in Singapore had low coverage at both 8782 and 28144 (≤10x), and three T/T genomes sampled in Wuhan had low sequencing depth and indeterminate nucleotide assignment at position 8782 (table S1). Further, the authors of eleven C/C genomes sampled in Wuhan and Sichuan confirmed that low sequencing depth at position 8782 led to the erroneous assignment of intermediate haplotypes.

C/C and T/T genomes continue to be observed throughout the pandemic as a result of convergent evolution, including T/T aboard the Diamond Princess cruise ship outbreak and subsequent COVID-19 waves in New York City and San Diego (fig. S2 to S5 and supplementary text). Instances of convergent evolution are identifiable because SARS-CoV-2 phylogenies exist in ‘near-perfect’ tree space where topology can be inferred with high accuracy (*12*). These findings cast doubt on the claim that transitional C/C or T/T haplotypes between lineages A and B circulated in humans, reopening the door to the hypothesis that lineages A and B represent separate zoonotic introductions.

### Progenitor genome reconstruction

To better understand SARS-CoV-2 mutational patterns, we reconstructed the genome of a hypothetical progenitor of SARS-CoV-2. Using maximum likelihood ancestral state reconstruction across 15 non-recombinant regions of SARS-CoV-2 and closely related sarbecovirus genomes sampled from bats and pangolins (*13*), we inferred the genome of this recombinant common ancestor (“recCA”) (figs. S6 and S7 and supplementary text). The recCA differed from Hu-1 by just 381 substitutions, including C8782T and T28144C. It is more informative than an outgroup sarbecovirus because it accounts for the closest relative across all recombinant segments (figs. S8 to S14 and supplementary text) (*14*), and, as an internal node on the phylogeny, is more genetically similar to SARS-CoV-2 than any extant sarbecovirus.

### Reversions across the early pandemic phylogeny

**Fig. 1.**
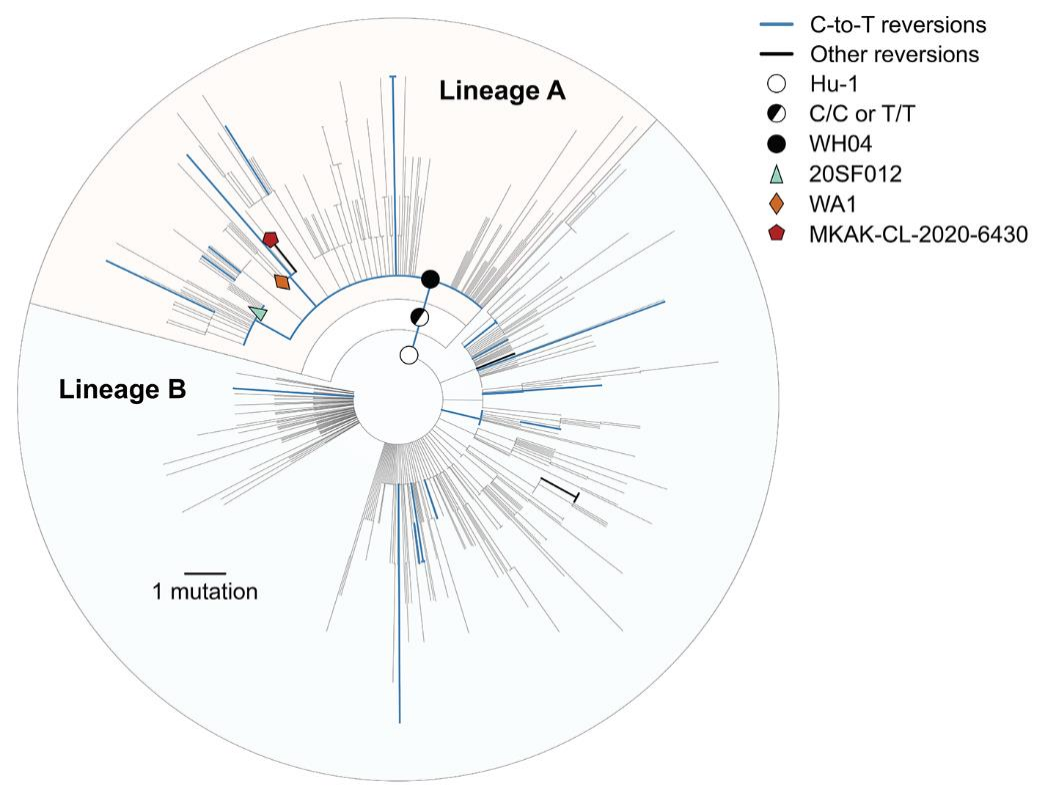
Maximum likelihood phylogeny of the early SARS-CoV-2 pandemic, showing nucleotide reversions and putative candidates for the ancestral haplotype at the most common recent ancestor (MRCA). Putative ancestral haplotypes are identified with colored shapes. Reversions from the Hu-1 reference genotype to the recCA are colored. Blue represents C-to-T reversions and black indicates all other reversions. The tree is rooted on Hu-1 to show reversion dynamics to the recCA.

The ubiquity of SARS-CoV-2 reversions (*i.e.*, mutations from Hu-1 toward the recCA) indicates that genetic similarity to related viruses is a poor proxy for the ancestral haplotype. We observe 23 unique reversions and 631 unique substitutions (excluding reversions) across the SARS-CoV-2 phylogeny from the COVID-19 pandemic up to 14 February 2020 (Fig. 1). Substitutions were overrepresented at the 381 sites separating the recCA from Hu-1 (23/381 = 6.04%), compared with substitutions at all other sites (631/29,134 = 2.17%).

Most reversions were C-to-T mutations (19/23 = 82.6%), matching the mutational bias of SARS-CoV-2 (*15*–*17*). Genomes with C-to-T reversions can be found within lineage A, including C18060T (lineage A.1; *e.g.*, WA1) and C29095T (*e.g.*, 20SF012), as well as C24023T, C25000T, C4276T, and C22747T in mid-late January and February 2020. Hence, triple revertant genomes, like WA1 and 20SF012, are neither unique nor rare. We also identified a lineage A genome (Malaysia/MKAK-CL-2020-6430/2020), sampled on 4 February 2020 from a Malaysian citizen traveling from Wuhan whose only four mutations from Hu-1 are all reversions (lineage A.1+T6025C) (Fig. 1). Therefore, no highly revertant haplotype can automatically be assumed to represent the MRCA of SARS-CoV-2, especially when these reversions are most often the result of C-to-T mutations. In fact, we continue to observe these reversion patterns throughout the pandemic, including in the emergence of WHO-named variants (figs. S15 and S16).

### Inferring the MRCA of SARS-CoV-2

To infer the ancestral SARS-CoV-2 haplotype, we developed a non-reversible, random-effects substitution process model in a Bayesian phylodynamic framework that simultaneously reconstructs the underlying coalescent processes and the sequence of the MRCA of the SARS-CoV-2 phylogeny. The random-effects substitution model captures the C-to-T transition and G-to-T transversion biases (fig. S17 and supplementary text). Using this model, referred to as the unconstrained rooting (fig. S18A), we inferred the ancestral haplotype of the 787 lineage A and B genomes sampled by 14 February 2020.

Our unconstrained rooting strongly favors a lineage B or C/C ancestral haplotype and shows that a lineage A ancestral haplotype is inconsistent with the molecular clock [Bayes factor (BF) = 48.1] (Table 1). Lineage B exhibits more divergence from the root of the tree than would be expected if lineage A were the ancestral virus in humans (figs. S19 and S20). The T/T ancestral haplotype was also disfavored (BF>10), likely because of the C-to-T transition bias (fig. S17). We acknowledge that the timing of the earliest sampled lineage B genomes associated with the Huanan market could bias rooting inference toward lineage B haplotypes; however, lineage A was still disfavored after excluding all market-associated genomes (BF=11.0).

**Table 1.** Posterior probabilities of inferred ancestral haplotype at the MRCA of SARS-CoV-2. Positions 8782 and 28144 are indicated in parentheses. Representative genome is that with its sequence matching the haplotype. “No market” excludes 15 market-associated genomes (13 lineage B genomes associated with the Huanan market plus one lineage A and one lineage B genome not associated with the Huanan market). *BF > 10. **BF > 100. ***BF > 1000; BFs are in favor of hypothesis rejection.

**Haplotype**	**Mutations from Hu-1 reference**	**Representative genome**	**Phylodynamic analysis**		
**Unconstrained** **(%)**	**No market** **(%)**	**recCA** **(%)**
B (C/T)	N/A	Hu-1	80.85^†^	62.96†	8.18
A (T/C)	C8782T+T28144C	WH04	1.68*	5.73*	77.28†
C/C	T28144C	N/A	10.32	23.02	10.49
T/T	C8782T	N/A	0.92*	1.68*	3.71*
A+C29025T (T/C)	C8782T+T28144C+C29095T	20SF012	<0.01***	<0.01***	0.20**
A.1 (T/C)	C8782T+T28144C+C18060T	WA1	<0.01***	<0.01***	0.04***

†Haplotype with greatest posterior probability; reference for BF.

Even though sequence similarity to closely related sarbecoviruses alone is insufficient to determine the SARS-CoV-2 ancestral haplotype, this similarity can inform phylodynamic inference. Rather than rely on outgroup rooting [fig. S18B and (*18*)], we developed a rooting method that assigns the recCA as the progenitor of the inferred SARS-CoV-2 MRCA (fig. S18C). As opposed to the unconstrained rooting, the recCA root favored a lineage A haplotype over lineage B, although support for C/C was unchanged (Table 1). Our results were insensitive to the method of breakpoint identification in the recCA (supplementary text).

The A.1 and A+C29095T proposed ancestral haplotypes were strongly rejected by all the phylodynamic analyses, even when rooting with recCA or bat sarbecovirus outgroups, which include both C18060T and C29095T (Table 1 and data S3). Hence, WA1-like and 20SF012-like haplotypes cannot plausibly represent the MRCA of SARS-CoV-2 as previously suggested (*19*–*21*): the similarity of these genomes to the recCA is due to C-to-T reversions. Haplotypes not reported in Table 1 were similarly rejected (data S3).

We inferred the tMRCA for SARS-CoV-2 to be 11 December 2019 (95% HPD: 25 November–12 December) using unconstrained rooting. It has been suggested that a phylogenetic root in lineage A would produce an older time of most recent common ancestor (tMRCA) than a lineage B rooting (*21*). Therefore, we developed an approach to assign a haplotype as the SARS-CoV-2 MRCA and inferred the tMRCA (*i.e.*, A, B, C/C, A.1 or A+C29095T) (fig. S18D). The tMRCA was consistent with the recCA-rooted and fixed ancestral haplotype analyses (table S2 and supplementary text).

We infer only three plausible ancestral haplotypes: lineage A, lineage B, and C/C. However, the inability to reconcile the molecular clock at the outset of the COVID-19 pandemic with a lineage A ancestor without information from related sarbecoviruses (*e.g.*, the recCA) requires us to question the assumption that both lineages A and B resulted from a single introduction.

### Separate introductions of lineages A and B

We next sought to determine whether a single introduction from one of the plausible ancestral haplotypes (lineage A, lineage B, or C/C) is consistent with the SARS-CoV-2 phylogeny. We simulated SARS-CoV-2-like epidemics (*22*, *23*) with a doubling time of 3.47 days [95% highest density interval (HDI) across simulations: 1.35–5.44] (*24*–*26*) to account for the rapid spread of SARS-CoV-2 before it was identified as the etiological agent of COVID-19 (figs. S21 and S22, tables S3 and S4, and supplementary text). We then simulated coalescent processes and viral genome evolution across these epidemics to determine how frequently we recapitulated the observed SARS-CoV-2 phylogeny.

**Fig. 2.**
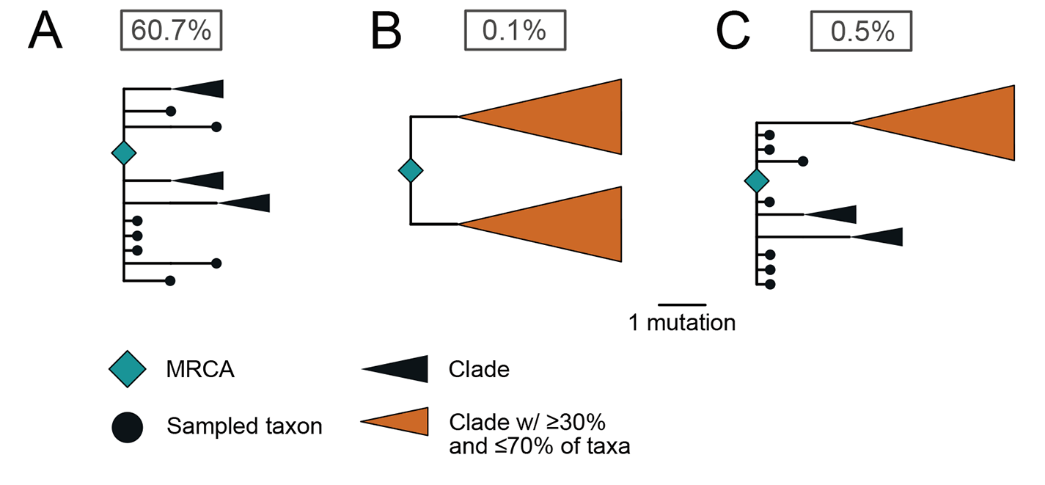
Probability of phylogenetic structures arising from a single introduction of SARS-CoV-2 in epidemic simulations. (**A**) A large polytomy of at least 20 descendent lineages, consistent with the base of both lineages A and B. (**B**) Topology matching a C/C ancestral haplotype: two clades each one mutation from the ancestor, both with polytomies of at least 20 descendent lineages. (**C**) Topology matching either a lineage A or lineage B ancestral haplotype: a basal polytomy with at least 20 descendent lineages including a large clade separated by two mutations, also possessing a polytomy of at least 20 descendent lineages. Basal taxa have short branch lengths for clarity. The probability of each phylogenetic structure after a single introduction is reported in the box.

Lineages A and B comprise 35.2% and 64.8% of the early SARS-CoV-2 genomes, and each lineage is characterized by a large polytomy (*i.e.*, many sampled lineages descending from a single node on the phylogenetic tree), with the base of lineages A and B being the two largest polytomies observed in the early pandemic (Fig. 1). Furthermore, large polytomies are characteristic of SARS-CoV-2 introductions into geographical regions at the start of the pandemic (e.g., fig. S23) (*11*, *27*–*29*) and would similarly be expected to occur after a successful introduction of SARS-CoV-2 into humans. Congruently, the most common topology in our simulations is a large basal polytomy (with ≥100 descendent lineages), present in 47.5% of simulated epidemics (Fig. 2A).

In contrast, a topology corresponding to a single introduction of an ancestral C/C haplotype, characterized by two clades, each comprising ≥30% of the taxa, possessing a large polytomy at the base, and separated from the MRCA by one mutation (Fig. 2B), was only observed in 0.1% of our simulations. Further, a topology corresponding to a single introduction of an ancestral lineage A or lineage B haplotype, characterized by a large basal polytomy and a large clade, comprising between 30% and 70% of taxa, two mutations from the root with no intermediate genomes, was observed in only 0.5% of our simulations (Fig. 2C, see supplementary text for details).

Our epidemic simulations do not support a single introduction of SARS-CoV-2 giving rise to the observed phylogeny. We therefore quantified the relative support for two introductions resulting in the empirical topology. By synthesizing posterior probabilities of inferred ancestral haplotypes, frequencies of topologies in epidemic simulations, and the expected relationships between these haplotypes and topologies, we infer strong support favoring separate introductions of lineages A and B (BF=61.6 and BF=60.0 using the recCA and unconstrained rooting, respectively; see Methods). This support is robust across shorter and longer doubling times, varying ascertainment rates, and minimum polytomy size (tables S4 and S5).

**Fig. 3.**
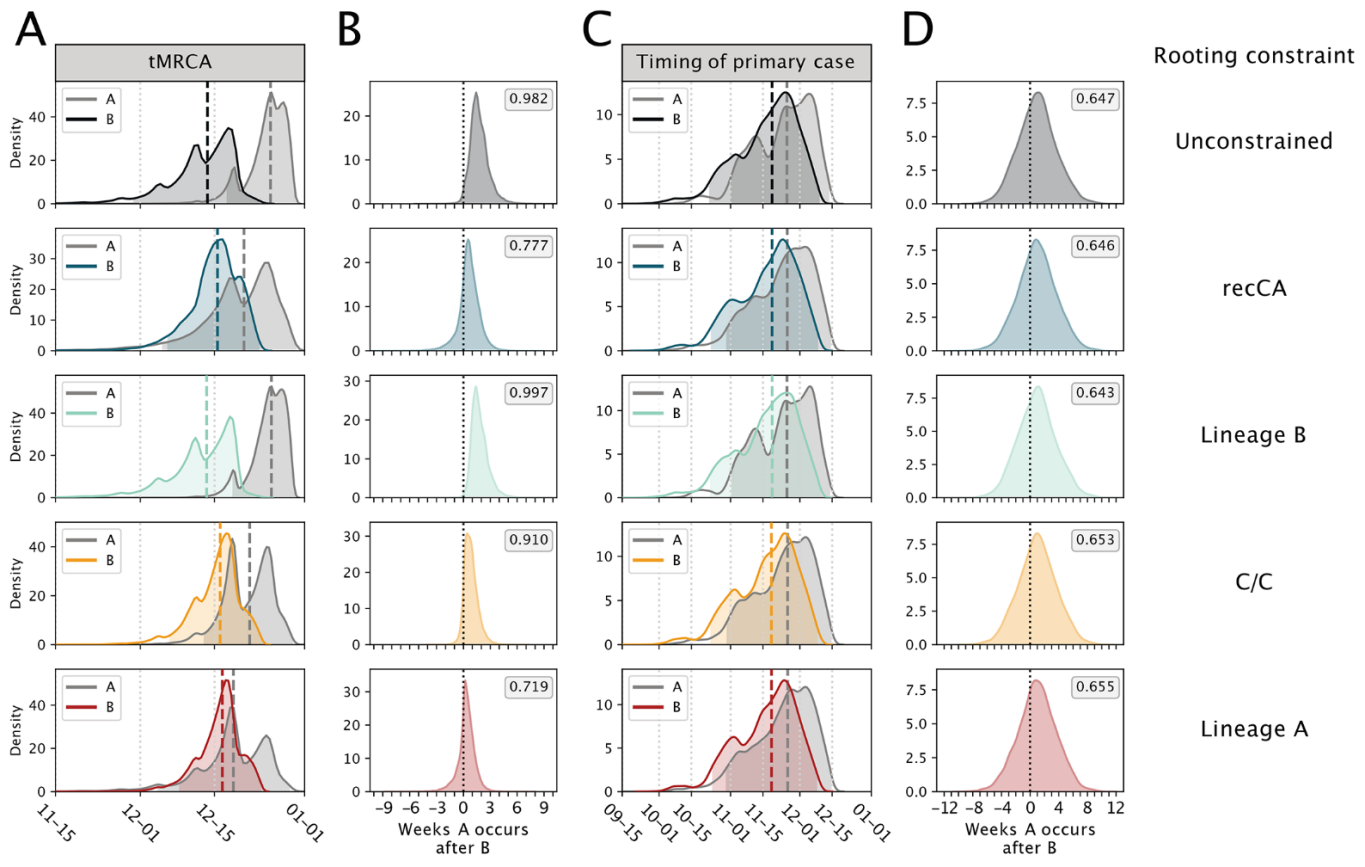
Comparison of the tMRCA and primary case dates for lineage A and lineage B across rooting strategies. Each row represents a different rooting constraint in phylodynamic analysis, with lineage B, C/C and lineage A representing a fixed ancestral haplotype. (**A**) The tMRCA for lineages A and B. (**B**) The number of weeks the tMRCA of lineage A occurs after the tMRCA of lineage B. (**C**) The timing of the primary case for lineages A and B. (**D**) The number of weeks the time of the primary case of lineage A occurs after the time of the primary case of lineage B. Long dashed lines indicate the median and shading represents the 95% HPD for each distribution. Short dashed lines indicate 0 weeks difference between lineages A and B. Posterior probability that lineage A originated after lineage B is reported in the grey box.

If lineages A and B arose from separate introductions, then the MRCA of SARS-CoV-2 was not in humans, and it is the tMRCAs of lineages A and B that are germane to the origins of SARS-CoV-2 (i.e, not the timing of their shared ancestor). Rooting with the recCA, we inferred the median tMRCA of lineage B to be 15 December (95% HPD: 5 December to 23 December) and the median tMRCA of lineage A to be 20 December (95% HPD: 5 December to 29 December) (Fig. 3A). The tMRCA of lineage B consistently predates the tMRCA of lineage A (Fig. 3B). These results are robust to using unconstrained rooting, fixing the ancestral haplotype, and excluding market-associated genomes (Fig. 3, A and B; table S2; and supplementary text).

### Timing the introductions of lineages A and B

The primary case, the first human infected with a virus in an outbreak, could precede the tMRCA if basal lineages went extinct during cryptic transmission (*23*, *30*, *31*). The index case, the first identified case, is rarely also the primary case (*32*, *33*). We next used an extension of our previously published framework combining epidemic simulations and phylodynamic tMRCA inference [see Methods; (*23*, *30*, *31*)] to infer the timing of the lineage B and lineage A primary cases, accounting for both the index case symptom onset date and earliest documented COVID-19 hospitalization date.

The earliest unambiguous case of COVID-19, with symptom onset on 10 December and hospitalization on 16 December, was a seafood vendor at the Huanan market. Unfortunately no published genome is available for this case (*8*). Nonetheless, we can reasonably assume this individual had a lineage B virus (supplementary text), as an environmental sample (EPI_ISL_408512) from the stall this vendor operated was lineage B. The earliest lineage A genome (IME-WH01) is from a familial cluster where the earliest symptom onset is 15 December and earliest hospitalization is 25 December (*34*). Accounting for these dates and using the recCA rooting, we inferred the infection date of the lineage B primary case to be 18 November (95% HPD: 23 October to 8 December) and the infection date of the primary case of lineage A to be 25 November (95% HPD: 29 October to 14 December). The lineage B primary case predated that of lineage A in 64.6% of the posterior sample, by a median of 7 days (Fig. 3D and table S6).

Our lineage A and B primary case inference is robust to rooting on the recCA and fixing the plausible ancestral haplotype to lineage A, lineage B, or C/C, as well as different index case dates, accounting for only hospitalization dates, and varying growth rates and ascertainment rates (tables S7 to S10 and supplementary text). Therefore, our results indicate that lineage B was introduced into humans no earlier than late-October and likely in mid-November 2019, and the introduction of lineage A occurred within days to weeks of this event.

**Fig. 4.**
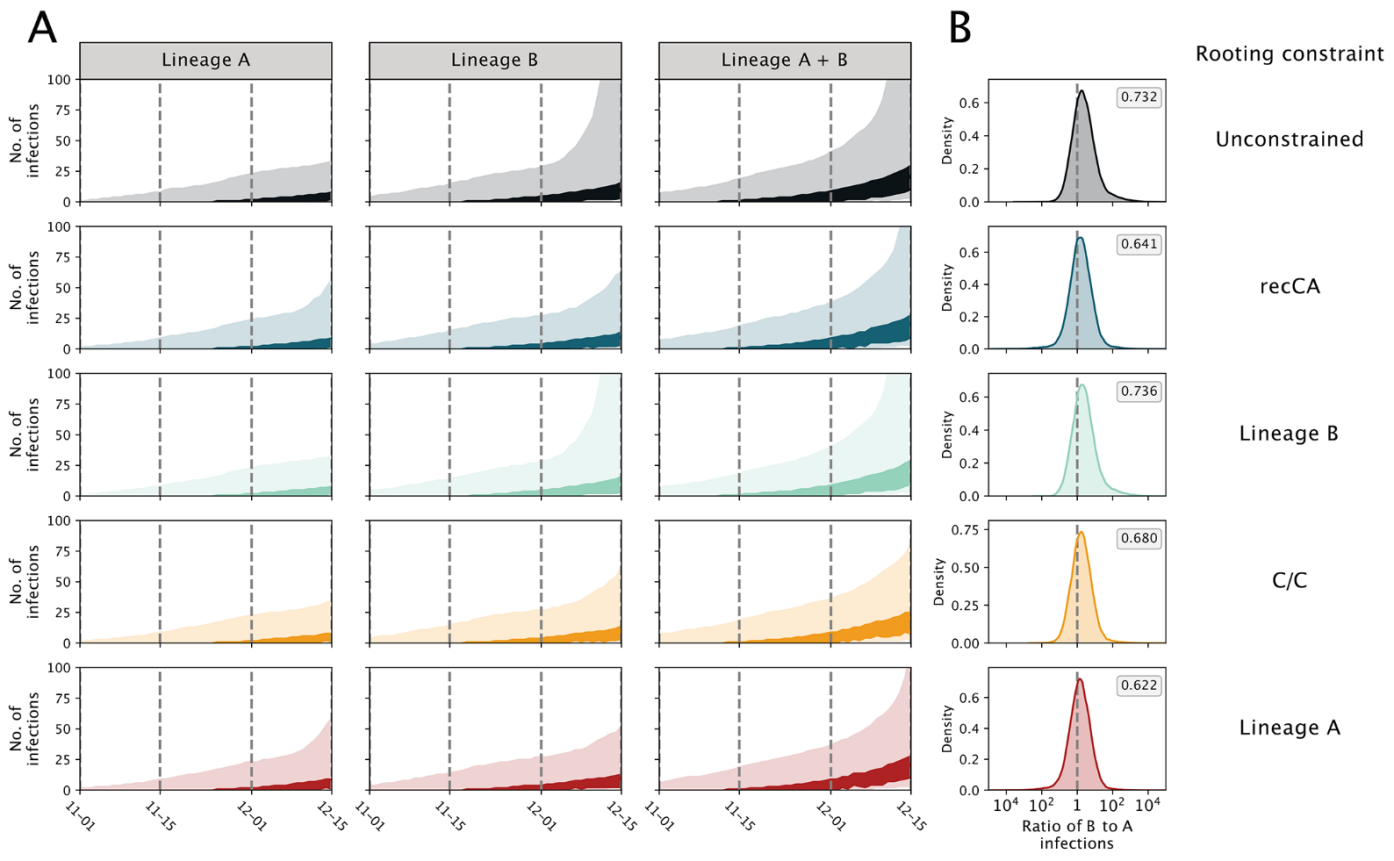
Dynamics of simulated SARS-CoV-2 epidemics resulting from separate introductions of lineages A and B. Each row represents a different rooting constraint in phylodynamic analysis, with lineage B, C/C and lineage A representing a fixed ancestral haplotype. (**A**) Estimated number of infections. The header of each column indicates whether the number of infections are caused by lineage A, lineage B, or the two lineages combined. Darker and lighter shading represent the 50% and 95% HPD, respectively. (**B**) The log ratio of lineage B to lineage A infections on 15 December 2019. Posterior probability of having more lineage B infections than lineage A reported in the grey box.

We then inferred the number of ascertained infections and hospitalizations arising from these separate introductions. We find that an earlier introduction of lineage B leads to a faster rise in lineage B-associated infections, dominating the simulated epidemics (Fig. 4) and recapitulating the predominance of lineage B observed in China in early 2020 (*35*). Similarly, simulated lineage B hospitalizations are more common than those from lineage A through January 2020 (fig. S24). We observe these patterns regardless of rooting strategy (unconstrained or recCA), ancestral haplotype (B, A, or C/C) (Fig. 4 and tables S11 and S12), and doubling time (figs. S25 to S28).

### Minimal cryptic circulation of SARS-CoV-2

We do not see evidence for substantial cryptic circulation before December 2019 (Fig. 4), even if we assume a single introduction (fig. S29 and supplementary text). Our simulated epidemics have a median of three (95% HPD 1-18) cumulative infections at the tMRCA, with 99% of simulated epidemics resulting in at most 33 infections (table S13 and supplementary text). Further, it is unlikely there were any COVID-19 related hospitalizations before December (*36*), as the simulated epidemics show a median of zero (95% HPD: 0–2) hospitalizations by 1 December 2019. These results are in accordance with the lack of a single SARS-CoV-2-positive sample among tens of thousands of serology samples from healthy blood donors from September to December 2019 (*37*) and thousands of specimens obtained from influenza-like illness patients at Wuhan hospitals from October to December 2019 (*34*). Therefore, there was likely extremely low prevalence of SARS-CoV-2 in Wuhan before December 2019. Even when we simulated epidemics with a longer doubling time, resulting in an earlier timing of the primary cases (tables S8 and S10), there were still few infections prior to December 2019 (table S13).

### Additional introductions

The extinction rate of our simulated epidemics (*i.e.*, simulations that did not produce self-sustaining transmission chains) indicate there were likely multiple failed introductions of SARS-CoV-2. Similar to our previous findings (*23*), 77.8% of simulated epidemics went extinct. These failed introductions produced a mean of 2.06 infections and 0.10 hospitalizations; hence, failed introductions could easily go unnoticed. If we treat each SARS-CoV-2 introduction, failed or successful, as a Bernoulli trial and simulate introductions until we see two successful introductions, we estimate that eight (95% HPD: 2–23) introductions led to the establishment of both lineage A and B in humans.

### Limitations

Our analysis of the putative intermediate haplotypes suggests there remain lineage assignment errors between lineages A and B, particularly of genomes sampled in January and February of 2020, which could influence the precision of the phylogenetic topology and tMRCA inference. Importantly, we lack direct evidence of a virus closely related to SARS-CoV-2 in non-human mammals at the Huanan market or its supply chain. The genome sequence of a virus directly ancestral to SARS-CoV-2 would provide more precision regarding the timing of the introductions of SARS-CoV-2 into humans and the epidemiological dynamics prior to its discovery. Although we simulated epidemics across a range of plausible epidemiological dynamics, our models represent a timeframe prior to the ascertainment of COVID-19 cases and sequencing of SARS-CoV-2 genomes and thus prior to when these models could be empirically validated.

## Discussion

The genomic diversity of SARS-CoV-2 during the early pandemic presents a paradox. Lineage A viruses are at least two mutations closer to bat coronaviruses, indicating that the ancestor of SARS-CoV-2 arose from this lineage. However, lineage B viruses predominated early in the pandemic, particularly at the Huanan market, indicating that this lineage began spreading earlier in humans. Further complicating this matter is the molecular clock of SARS-CoV-2 in humans, which rejects a single-introduction origin of the pandemic from a lineage A virus. Here, we resolve this paradox by showing that early SARS-CoV-2 genomic diversity and epidemiology is best explained by at least two separate zoonotic transmissions, in which lineage A and B progenitor viruses were both circulating in non-human mammals prior to their introduction into humans (figs. S30 and S31).

The most probable explanation for the introduction of SARS-CoV-2 into humans involves zoonotic jumps from as-yet undetermined, intermediate host animals at the Huanan market (*34*, *38*, *39*). Through late-2019 the Huanan market sold animals that are known to be susceptible to SARS-CoV-2 infection and capable of intra-species transmission (*40*–*42*). The presence of potential animal reservoirs, coupled with the timing of the lineage B primary case and the geographic clustering of early cases around the Huanan market (*39*), support the hypothesis that SARS-CoV-2 lineage B jumped into humans at the Huanan market in mid-November 2019.

In a related study (*39*), we show that the two earliest lineage A cases are more closely positioned geographically to the Huanan market than expected compared with other COVID-19 cases in Wuhan in early 2020, despite having no known association with the market. This geographic proximity is consistent with a separate and subsequent origin of lineage A at the Huanan market in late-November 2019. The presence of lineage A virus at the Huanan market was confirmed by Gao *et al*. (*43*) from a sample taken from discarded gloves.

The high extinction rate of SARS-CoV-2 transmission chains, observed in both our simulations and real-world data (*44*), indicates that the two zoonotic events establishing lineages A and B may have been accompanied by additional, cryptic introductions. However, such introductions could easily be missed, particularly if their subsequent transmission chains quickly went extinct or the introduced viruses had a lineage A or B haplotype. Failed introductions of intermediate haplotypes are also possible. Critically, we have no evidence of subsequent zoonotic introductions in late-December leading up to the closure of the Huanan market on 1 January 2020. By then, the susceptible host animals that had been documented at the market during the previous months were no longer found in the Huanan market (*34*).

Other coronavirus epidemics and outbreaks in humans, including SARS-CoV-1, MERS-CoV, and, most recently, porcine deltacoronavirus in Haiti, have been the result of repeated introductions from animal hosts (*45*–*47*). These repeated introductions were easily identifiable because human viruses in these outbreaks were more closely related to viruses sampled in the animal reservoirs than to other human viruses. However, the genomic diversity within the putative SARS-CoV-2 animal reservoir at the Huanan market was likely shallower than that seen in SARS-CoV-1 and MERS-CoV reservoirs (*45*, *46*, *48*). Hence, even though lineages A and B had nearly identical haplotypes, their MRCA likely existed in an animal reservoir. The ability to disentangle repeated introductions of SARS-CoV-2 from a shallow genetic reservoir has previously been shown in the early SARS-CoV-2 epidemic in Washington state, where two viruses, separated by two mutations, were independently introduced from, and shared an MRCA in, China (figs. S23 and S30 and supplementary text) (*11*).

Successful transmission of both lineage A and B viruses after independent zoonotic events indicates that evolutionary adaptation within humans was not needed for SARS-CoV-2 to spread (*49*). We now know that SARS-CoV-2 can readily spread after reverse-zoonosis to Syrian hamsters (*Mesocricetus auratus*), American mink (*Neovison vison*), and white-tailed deer (*Odocoileus virginianus*), indicating its host generalist capacity (*50*–*55*). Furthermore, once an animal virus acquires the capacity for human infection and transmission, the only remaining barrier to spillover is contact between humans and the pathogen. Thereafter, a single zoonotic transmission event indicates the conditions necessary for spillovers have been met, which portends additional jumps. For example, there were at least two zoonotic jumps of SARS-CoV-2 into humans from pet hamsters in Hong Kong (*56*) and dozens from minks to humans on Dutch fur farms (*52*, *53*).

We show that it is highly unlikely that SARS-CoV-2 circulated widely in humans earlier than November 2019 and that there was limited cryptic spread, with, at most, dozens of SARS-CoV-2 infections in the weeks leading up to the inferred tMRCA, but likely far fewer. By late-December, when SARS-CoV-2 was identified as the etiological agent of COVID-19 (*8*), the virus had likely been introduced into humans multiple times as a result of persistent contact with a viral reservoir.

## Materials and methods summary

Materials and methods described in full detail can be found in the supplementary materials.

### Sequence data

We queried the GISAID database (*57*), GenBank, and National Genomics Data Center of the China National Center for Bioinformatics (CNCB), for complete high-coverage SARS-CoV-2 genomes collected by 14 February 2020, resulting in a dataset of 787 taxa belonging to lineages A and B and 20 taxa with C/C or T/T haplotypes. Genomes were aligned using MAFFT v7.453 (*58*) to the SARS-CoV-2 reference genome (Wuhan/Hu-1/2019) and 388 sites were masked at the 5′ and 3′ ends and at sites based on De Maio *et al*. (*59*). All genome accessions are available in data S1 and S2.

### Progenitor genome reconstruction and reversion analysis

We reconstructed the progenitor of SARS-CoV-2, the recombinant common ancestor (the recCA). We (i) inferred a maximum likelihood tree of 31 sarbecovirus genomes (SARS-CoV-2 and 30 closely related sarbecoviruses sampled from bats and pangolins) across 15 predefined non-recombinant regions (*13*) with IQ-TREE v2.0.7 (*60*), (ii) inferred the sequence of the ancestor of SARS-CoV-2 in each tree with TreeTime v0.8.1 (*61*), and (iii) concatenated the resulting sequences. We next inferred a maximum likelihood tree of the 787 SARS-CoV-2 taxa with IQ-TREE and performed ancestral state reconstruction with TreeTime to identify substitutions that were reversions from Wuhan-Hu-1 to the recCA across the SARS-CoV-2 phylogeny.

### Phylodynamic inference and epidemic simulations

We performed phylodynamic inference using ​​BEAST v1.10.5 (*62*) with the 787-taxa dataset to infer the ancestral haplotype and the tMRCA of SARS-CoV-2 (and the tMRCAs of lineages A and B), employing a non-reversible random-effects substitution model and exploring unconstrained rooting, recCA-rooting, fixing the ancestral haplotype as a root, and outgroup rooting. SARS-CoV-2-like epidemics were simulated with FAVITES-COVID-Lite v0.0.1 (*22*, *63*) using a scale-free network of 5 million individuals and a customized extension of the SAPHIRE model (*64*), producing coalescent trees on which we simulated mutations. We calculated the Bayes factor comparing the support of two introductions of SARS-CoV-2 to one introduction by considering the posterior probabilities of the four most likely ancestral haplotypes from the phylodynamic inference (Lineage A, Lineage B, C/C, and T/T), the frequencies of the phylogenetic structures associated with introductions of these haplotypes in the epidemic simulations, and equal prior probabilities for each ancestral haplotype and one versus two introductions.

We connected the phylodynamic inference and epidemic simulations via a rejection sampling-based approach (*23*), accounting for the tMRCAs of lineages A and B and the earliest documented COVID-19 illness onset and hospitalization dates. We then inferred the timing of the introductions of lineages A and B and the infections and hospitalizations for each lineage. The proportion of epidemic simulations that went extinct (*i.e.*, no onward transmission by the end of the simulation) was used to approximate the number of SARS-CoV-2 introductions needed to result in two introductions with sustained onward transmission.

## Supplementary Materials

### This PDF file includes:

Materials and Methods Supplementary Text Figs. S1 to S31 Tables S1 to S15 References (*67* *–* *110*)

### Other Supplementary Material for this manuscript includes the following:

MDAR Reproducibility Checklist
