# Supplementary material for: The molecular epidemiology of multiple zoonotic origins of SARS-CoV-2
Source: Science. 2022 Jul 26:eabp8337. doi: 10.1126/science.abp8337 (PMC9348752; doi:10.1126/science.abp8337)
Supplement: Supplementary file 1 — Materials and Methods Supplementary Text Figs. S1 to S31 Tables S1 to S15 References ( 67 – 110 ) [file science.abp8337_sm.pdf]

Science
AAAS

Supplementary Materials for

The molecular epidemiology of multiple zoonotic origins of SARS-CoV-2

Jonathan E. Pekar *et al.*

Corresponding authors: Jonathan E. Pekar, jepekar@ucsd.edu; Marc A. Suchard, msuchard@ucla.edu; Kristian G. Andersen, andersen@scripps.edu; Michael Worobey, worobey@arizona.edu; Joel O. Wertheim, jwertheim@health.ucsd.edu

DOI: 10.1126/science.abp8337

The PDF file includes:

Materials and Methods
Supplementary Text
Figs. S1 to S31
Tables S1 to S15
References

Other Supplementary Material for this manuscript includes the following:

MDAR Reproducibility Checklist
Data S1 to S3

Materials and Methods

Sequence data. We queried the GISAID database SARS-CoV-2 viral genome alignment for sequences collected by 14 February 2020 (*57*). We selected this date to have a data set whose size is appropriate for Bayesian phylodynamic analyses (*i.e.*, under 1000 genomes). We restricted our data set to sequences that (i) were ≥29,000 nucleotides, (ii) had high coverage with ≤0.5% unique amino acid mutations, (iii) had fewer than 1% 'N's, (iv) were not identified as potentially problematic via NextStrain (*67*), and (v) had a year-month-day sampling date reported. We additionally queried for the National Genomics Data Center, part of the China National Center for Bioinformatics (CNCB), for additional genomes collected by 14 February 2020 not represented on GISAID. Genomes described in the WHO report (*34*) to have erroneous mutations were updated, and if the virus was sequenced multiple times, the corrections belonging to the genome with the highest coverage were used. The first 88 and last 195 nucleotides of each genome were masked due to poor evidence of homology, and an additional 105 sites were masked based on the work by De Maio *et al.* (*59*), leading to a total of 388 masked sites. Genomes with an ambiguous nucleotide (*e.g.*, Y or N) at site 8782 or 28144 were excluded. We excluded an additional 20 genomes from the primary phylodynamic analyses, because these sequences contained either C8782T or T28144C, but not both, for reasons described in the main text and De Maio et al. (2020) (*10*). The final dataset from the early COVID-19 pandemic comprised 787 taxa. A list of GISAID accessions are available in Data S1, and a list of CNCB and GenBank accessions are available in S2. All GISAID accessions are also available through GISAID when using the identifier *EPI_SET_20220305ud*.

Examining shared mutations between early C/C and T/T genomes and lineages A and B. The 787 SARS-CoV-2 genome dataset and the 20 genomes with either C8782T or T28144C were aligned with MAFFT v7.453 (*58*) (options --auto --keeplength --addfragments) to reference genome Hu-1 (GenBank accession MN908947.3; GISAID accession EPI_ISL_402125). We then identified pairs of genomes comprising an intermediate genome (C/C or T/T) and a major lineage (lineage A or B) that shared derived mutations.

We additionally examined 83 genomes from the Diamond Princess outbreak, aligned to reference genome Hu-1 with MAFFT. We inferred a maximum likelihood tree using IQ-TREE 2 v2.0.7 (*60*) under a GTR+F+I substitution model, which was visualized using FigTree (*68*).

Sequencing quality of early C/C and T/T genomes. We aligned reads FASTQ files belonging to EPI_ISL_413017, a C/C genome from South Korean, and EPI_ISL_462306, a T/T genome from Singapore, using Minimap2 (*69*), sorted the subsequent SAM files using samtools (*70*), and called variants using iVar (*71*). The variant calls were then manually inspected for depth and indeterminacy at specific sites, including 8782 and 28144. The raw data for EPI_ISL_413017 and EPI_ISL_462306 are available at https://www.ncbi.nlm.nih.gov/sra, with project IDs PRJNA806767 and PRJNA802993, respectively.

C/C and T/T genomes in San Diego. The SEARCH consortium has sequenced over 35,000 genomes from San Diego during the course of the pandemic, by 3 February 2022. We generated a multiple sequence alignment of these genomes using Minimap2 and gofasta (*72*). We queried this alignment for consensus genomes with both C8782T and T28144, or both C8782 and T28144C, and validated these mutations by checking the read depth and allele frequency in the original alignment files.

The consensus genomes and associated BAM files are publicly available at https://github.com/andersen-lab/HCoV-19-Genomics, and the genome accessions are listed in Data S2. The tree figures were rendered using baltic (*73*).

T/T genomes in NYC. Molecular surveillance conducted by the New York City Public Health Laboratory, part of the Department of Health and Mental Hygiene, has sequenced >5000 SARS-CoV-2 genomes through the end of 2021. We queried these data for genomes with both C8782T and T28144 in the consensus sequence and validated the consensus sequence by checking the read depth and allele frequency from the primer removed BAM files. The genome accessions are listed in Data S1. The tree figure was rendered using baltic.

Constructing the recombinant common ancestor. We used the aligned sarbecovirus genomes (5' non-coding and poly-A tail excluded; includes the entirety of the SARS-CoV-2 genome; genome accession IDs available in Data S1 and S2) and 14 breakpoints inferred from GARD (*74*) and provided by Sarah Temmam and Marc Eloit (*13*) to infer the phylogenetic history of 15 non-recombinant regions. We inferred a maximum likelihood tree of the animal viruses in the alignment and Hu-1 for each non-recombinant region using IQ-TREE 2 under a GTR+F+G+I substitution model. We midpoint-rooted each maximum likelihood tree and used TreeTime v0.8.1 (*61*) to perform ancestral sequence reconstruction for each fragment. The genome belonging to the parent node of Hu-1 for each non-recombinant region was inferred, and these inferred regions were concatenated to construct the recombinant common ancestor (recCA) of SARS-CoV-2 in the sarbecovirus clade. The mid-point root of each tree is always separated from the parent node of SARS-CoV-2 by at least several internal nodes, indicating that the recCA inference would not be sensitive to rooting.

There are 382 substitutions between Hu-1 and the recCA, and one of these sites is masked in the phylodynamic analyses. Ignoring the 387 masked sites of the remaining 29,521 sites, there are 29,134 sites identical between Hu-1 and the recCA. When we used a different SARS-CoV-2 genome (*e.g.*, WH04, WA1) to construct the recCA, the recCA sequence was consistent, indicating the recCA can reliably be used as an ancestor of SARS-CoV-2.

We created a simplot of the sarbecovirus genomes and the recCA against Hu-1 using RDP4 (*75*), and our phylogenies were visualized using FigTree. The genome accessions are listed in Data S1 for genomes from GISAD and Data S2 for genomes from GenBank.

recCA robustness analysis. To examine how sensitive the recCA and downstream analyses were to the recombination inference method, we constructed a second recCA. Using the alignment provided by

Sarah Temmam and Marc Eloit, we re-inferred breakpoints with 3SEQ (*76*) with an approach based on Boni et al. (*77*). Briefly, after running 3SEQ on the 31-genome alignment, we (1) collected all inferred breakpoints into a single set, (2) complemented this set to generate the set of non-breakpoints, (3) grouped non-breakpoints into continuous breakpoint free regions, (4) reran 3SEQ on the all BFRs >3,000 nucleotides to examine them for mosaicism, (5) pooled the breakpoints from the first and second 3SEQ runs, and (6) collapsed all breakpoints within 100 nucleotides of each other into the most upstream (5') breakpoint. This resulted in 21 breakpoints and 22 BFRs. We next constructed a recCA using the same methods as above.

Reversions early in the pandemic. Here, we define mutations away from the Hu-1 reference genome toward the recCA, such as C8782T and T28144C, as reversions. The 787 genomes sampled by 14 February 2020 were aligned with MAFFT to Hu-1 (GISAID accession EPI_ISL_402125). The phylogenetic history of SARS-COV-2 in China was first inferred in a maximum likelihood framework in IQ-TREE 2 using a GTR+F+I model. We used TreeTime to perform ancestral state reconstruction on the maximum likelihood tree of 787 genomes, rooted on Hu-1. We determined which branches had reversions. Each unique reversion and non-reversion substitution was only counted once to account for phylogenetic uncertainty. The tree figure was rendered using baltic.

Reversions throughout the pandemic. We extended the reversion analysis from above to the following variants: Alpha (PANGO lineage B.1.1.7), Beta (B.1.351), Gamma (P.1), Delta (B.1.617.2), Epsilon 1 (B.1.427), Epsilon 2 (B.1.429), Zeta (P.2), Iota (B.1.526), and Kappa (B.1.617.1). To match the 52-day window for sequence collection from the early pandemic, we found the earliest 52-day window in GISAID for each variant that contained at least 750 sequences. The genomes for each lineage were then aligned using MAFFT to reference genome Hu-1. We next used IQ-TREE 2 and TreeTime as above to generate a phylogeny and perform ancestral state reconstruction. We followed the same protocol as above with the recCA to determine which branches had reversions. The tree figures were rendered using baltic.

To examine reversions across a subsample of the entire pandemic, we extracted the global tree and associated public genomes from Nextstrain (*67*) on 14 January 2022 and constrained it to sequences before November 2021. We aligned the genomes using MAFFT as above, used TreeTime with the NextStrain tree and alignment to perform ancestral state reconstruction, and determined reversions with the recCA. The genomes used for the clade and global pandemic reversion analysis can be found in Data S1 and S2, respectively.

Phylogenetic inference. Molecular clock phylodynamic inference was conducted using a Bayesian approach in BEAST v1.10.5 (*62*). For the primary analysis, we developed and employed a non-reversible, random-effects substitution model (described below), a strict molecular clock, a non-parametric skygrid prior with 20 grid points and a cut off of 0.37, which translates to 5 October 2019. To facilitate Markov chain Monte Carlo (MCMC) chain convergence, (i) we used our previous results of $7.9x10^{-4}$ and $6.8x10^{-5}$ substitutions/site/year as the mean and standard deviation, respectively, of a normal prior for the clock rate, and (ii) we initiated the MCMC sampling using the maximum likelihood

phylogeny that had been transformed into a chronogram via TempEst v1.5.3 (*78*). We ran four independent chains of 400 million generations, sub-sampling every 25 thousand iterations to continuous parameter log files, 100 thousand iterations for the tree file, and 100 thousand iterations for the ancestral state reconstruction of the most recent common ancestor (MRCA); the first 15% of the chains were discarded as burnin. Convergence and mixing was assessed in Tracer v1.7.1 (*79*) and all 4 chains were combined in LogCombiner, such that all relevant effective sample size (ESS) values were >200. The accession IDs can be found in Data S1 and S2, and the XML files can be found at (*65*). The outputs can be found at (*66*).

Random-effects substitution model. To accommodate the mutational bias in SARS-CoV-2 for C-to-T transitions (*15–17*), we developed a random-effects substitution model. This model employs a standard phylogenetic substitution model as a base model, incorporated as fixed effects, while the random effects allow each individual mutation rate (*e.g.*, C-to-T, separate from T-to-C) to be elevated (or decreased) relative to that model. Note that this makes the model non-reversible. We use HKY as the base model, which is defined by the parameters κ (governing the relative rate of transitions versus transversions) and $\boldsymbol{\pi}$ (governing the root and stationary frequencies). Working on the log-scale and denoting the random effect ϵ_{ij}, our HKY+RE model gives the log of the substitution rate matrix entries as,

$$log(q_{ij}) = log(\pi_j) + log(\kappa) + \epsilon_{ij} + log(\xi) \text{ if i>j is a transition}$$

or,

$$log(q_{ij}) = log(\pi_j) + \epsilon_{ij} + log(\xi) \text{ if i>j is a transversion,}$$

where ξ is a normalizing constant.

To accommodate among-site rate variation, we employ a proportion of invariable sites in the model, with a Uniform(0,1) prior. We place independent and identical Normal(mean=0,SD=σ_ϵ) priors on ϵ_{ij}, an improper infinite uniform prior on σ_ϵ, and a Normal(mean=0,SD=1.25) prior on log(κ). We fix $\boldsymbol{\pi}$ to the frequencies observed in the alignment.

Quantifying the support for ancestral haplotypes. We assume equally likely prior probabilities for each sequence in the ancestral state reconstruction posterior. Therefore, the Bayes factor (BF) in favor of sequence S_1 against another sequence S_0, given the data *D*, can be expressed as follows:

$$B_{10} = \frac{P(S_1|D)}{P(S_0|D)}$$

where $P(S|D)$ is the posterior probability of a sequence. Note that all BFs were calculated with the sequence comprising the highest posterior probability as S_1, and BFs for each phylodynamic model were calculated separately.

Phylogenetic inference with constrained roots and recCA. In a standard phylogenetic model, the sequences at all internal nodes (and the root) are integrated out. A prior distribution is required for the root sequence in order to compute the likelihood or sample ancestral states at nodes. Typically, the prior distribution for the root sequence assumes every site is drawn identically and independently from some multinomial distribution defining the prior probability of an A, C, G, or T nucleotide at any (and every) site. Here, we consider two novel approaches.

The first approach constrains the character state of the MRCA of all human SARS-CoV-2 sequences to be identical to a specific hypothesized ancestral haplotype (Fig 4B). We consider 6 ancestral haplotypes that match the sequences belonging to Wuhan Hu-1, the C/C haplotype (Hu-1 with T28144C), the T/T haplotype (Hu-1 with C8782T), WH04 (lineage A, or Hu-1 with C8782T and T28144C), 20SF2012 (WH04 with C29095T), and WA1 (lineage A.1, or WH04 with C18060T) . Note that in this model, the root of the tree is the MRCA of all 787 human SARS-CoV-2 sequences, and all 787 genomes, including the genomes that are identical to the ancestral haplotype, remain taxa—the tree is not rooted on them.

The second approach places a per-site prior distribution on the ancestral haplotype. Specifically, we add a branch ancestral to the MRCA and fix the sequence at the root to be the sequence of the recCA (Fig 4D). This approach places a prior distribution on the ancestral haplotype, as the root and MRCA are distinct in this model. At each site, this prior is determined by the nucleotide present in the recCA, the length of the branch leading to the MRCA, and the substitution model parameters. Our primary analysis uses this approach with the recCA based on the 14 breakpoints from GARD. We performed a robustness analysis by using this approach with the recCA based on the 21 breakpoints from 3SEQ.

BEAST sensitivity analyses. We performed a series of sensitivity analyses for the Bayesian phylogenetic inference: (i) unconstrained rooting with a GTR+F+I model; (ii) unconstrained rooting with unmasked sequences; (iii) unconstrained rooting excluding 15 market-associated genomes; (iv) unconstrained rooting excluding all genomes sampled from Wuhan; (v) unconstrained rooting including the 20 “intermediate” genomes sampled before 14 February 2020; (vi) unconstrained rooting but including the 16 C/C genomes sampled before 14 February 2020; (vii) unconstrained rooting but including the 4 T/T genomes sampled before 14 February 2020; (viii) recCA rooting using the recCA based on the 14 breakpoints from GARD and with a GTR+F+I model; (ix) outgroup rooting with RaTG13 as an outgroup; and (x) outgroup rooting with BANAL-20-52 as an outgroup.

The 7 July 2020 sampling date of BANAL-20-52, which post-dates the last included SARS-CoV-2 genome in the dataset by several months, creating a problematic months-long lineage for the coalescent skygrid prior. To accomodate this issue for sensitivity analysis rooted on BANAL-20-52, we truncate the branch leading to BANAL-20-52 at 14 February 2020. Compared to the overall duration of this branch (roughly 17 years), the effect of the truncation is negligible to the likelihood.

Inferring empirical doubling times. To determine the appropriate doubling time for the epidemic simulations, we inferred the doubling time of the early pandemic. We extracted the daily number of

cases from the WHO report (*34*) through the end of December 2019 and coupled it with the data from Li et al. (*80*). Because the case data included early cases that were later found to not associated with SARS-CoV-2 infection, we (i) removed the case from 2 December 2019 [as per the WHO report (*34*)] and (ii) shifted the case from 8 December to 16 December [as per Worobey (*8*)]. Additionally, we (iii) added a case to 10 December [as per Worobey (*8*)]. We used EpiNow2 and its default parameters with the daily case count to infer the doubling time of SARS-CoV-2 through 15 January 2020 (*81*).

Epidemic simulation. To explore the evolutionary dynamics during the beginning of the COVID-19 pandemic, we developed FAVITES-COVID-Lite (*63*), a simplified individual-based simulation pipeline based on FAVITES (*22*), and performed a series of epidemic simulations. First, we generated static contact networks comprising 5 million individuals (nodes) using NiemaGraphGen (*82*) under a preferential-attachment model using the Barabási–Albert algorithm (*83*). We used this algorithm to generate the contact networks, because its scale-free properties recapitulate infectious disease spread (*83*), including the superspreading dynamics of SARS-CoV-2 (*11, 23*). We chose to simulate a static contact network because our focus is on the number of people infected at the beginning of the epidemic, and we used an intermediate value of 16 contacts per day (mean degree), based on Mossong et al. (*85*).

We extended the SAPHIRE [Susceptible (S)-Ascertained (I)-Presymptomatic (P)-Hospitalized (H)-Not Ascertained (A)-Removed (R)-Exposed (E)] model developed by Hao et al. (*64*), and implemented in our previous study on the timing of the primary case (*23*), to have two ascertained compartments. Individuals from the first ascertained compartment can either enter the recovered compartment or an "ascertained-pre-hospitalization" (I_H) compartment, where they eventually transition to the hospitalized compartment. We extended the model with the I_H compartment to decouple the proportion of people hospitalized with the amount of time until hospitalization. We did not include the travel component of the original SAPHIRE model (*i.e.*, individuals flying into and out of Wuhan), because our focus was on the early dynamics of the pandemic before its spread outside of Wuhan. The dynamics of these compartments across time (*t*) are described by the following set of ordinary differential equations:

$$\frac{dS}{dt} = -\frac{bS(\alpha P + \alpha A + I + I_h)}{N}$$
$$\frac{dE}{dt} = \frac{bS(\alpha P + \alpha A + I + I_h)}{N} - \frac{E}{D_e}$$
$$\frac{dP}{dt} = \frac{E}{D_e} - \frac{P}{D_p}$$
$$\frac{dA}{dt} = \frac{(1-r)P}{D_p} - \frac{A}{D_i}$$
$$\frac{dI}{dt} = \frac{rP}{D_p} - \frac{I}{D_i}$$
$$\frac{dI_h}{dt} = \frac{hI}{D_i} - \frac{I_h}{D_q - D_i}$$

$$\frac{dH}{dt} = \frac{I_h}{D_q - D_i} - \frac{H}{D_h}$$
$$\frac{dR}{dt} = \frac{A + (1-h)I}{D_i} + \frac{H}{D_h}$$

We note that we rescale the transmission rate (*b*) reported in Table S3 and used for each analysis to the average degree centrality in the network (16).

We performed forward simulations using this extended model to generate a viral transmission network using GEMF (*86*). Simulated epidemics started with a single seed infection among our 5 million susceptible individuals, and infected nodes infect other nodes via Poisson processes. The parameters were primarily determined by Hao et al. (*64*) (Table S3), except we required half the ascertained population to become hospitalized with an average of 11 days between symptom onset and hospitalization, matching reports of hospitalization of the early pandemic in Wuhan (*87–89*). Each simulation was run for 100 days and produced an output documenting when individuals transitioned from one compartment to another throughout the entire simulation. We used these outputs to determine the number of individuals in a given compartment (*e.g.*, total infections, ascertained infections, unascertained infections, and hospitalized individuals) across each day in the simulation.

Once the forward simulations were complete, we subsampled the first 50,000 infected individuals. If there were fewer than 50,000 infections, we used each infected individual for subsequent analyses. Then, we sample a sequencing time for each ascertained individual from a uniform distribution that starts when they enter the first ascertained compartment and ends when they were recovered. To match real-world data from December 2019, we include only individuals with genome sampling times that occur after the first hospitalization of the simulated epidemic. Additionally, the primary case (*i.e.*, first infected individual in the simulation) is sampled regardless of ascertainment status. We sample the primary case regardless of ascertainment or hospitalization status to properly determine stable coalescence (described below). Unascertained individuals are not sampled.

Lastly, we provide the genome sampling times and transmission network to CoaTran (*82*), which uses a coalescent process to generate time trees. We then use a constant substitution rate of 9.2×10^{-4} substitutions/site/year (inferred from our primary BEAST results) to convert the branch lengths from years to substitutions per site to generate mutation trees.

For the primary analysis, we ran successive epidemic simulations until we reached 1100 successful simulations, defined as those simulations in which ≥400 people had become infected and ≥1 person was still infectious at the end of the simulation. Failed epidemics were those simulations that did not become established (*i.e.*, 0 infectious people at the end of the simulation) or had fewer than 400 people infected over the entire simulation; 3857 (77.8%) simulations failed to reach this epidemic threshold after 100 days. Our epidemics had a median doubling time of 3.47 days (95% range: 1.35-5.44), slightly shorter than the doubling times from Hao et al. (*64*) to match empirical estimates of the growth rate from before 1 January 2020 in Wuhan (*24*, *25*). Twelve of the 4957 simulations (0.2%) did not reach

400 infections but were still persisting at the end of the 100-day simulation. Given the rarity of these 12 simulations, it is highly improbable that SARS-CoV-2 was characterized by their growth dynamics.

All input parameters for the primary and sensitivity analyses can be found in (*65*), the output files and post-processed results can be found at doi (*66*).

Doubling time inference. To characterize the growth dynamics of our epidemic simulations and compare them to empirical growth dynamics, we estimated the doubling time of the primary epidemic simulations. For our primary simulations, we calculated both the cumulative doubling time and 14-day doubling time at each day in the simulation (prior to day 14, the 14-day doubling time is identical to the cumulative doubling time). However, because of the stochasticity inherent in these epidemic simulations, the simulations can be more easily compared to one another when the same number of individuals are infected. Therefore, we also estimated the cumulative and 14-day doubling times at certain numbers of infections (*e.g.*, 100 infections).

Based on the doubling time results (Fig. S22), we reason that at 1,000 infections, there is sustained transmission, the variability in doubling times across simulations has slightly decreased, and the 5 million node network has not yet started to saturate. Therefore, unless otherwise specified, the doubling times we report for each set of epidemic simulations is the 14-day doubling time when 1,000 individuals are infected.

Sensitivity analysis–faster rate of infection. We simulated epidemics for a more rapidly spreading virus using the same parameters from the primary analysis, except the transmission rate was increased from 0.28 to 0.38 per day (1.36x) and the simulation time was decreased from 100 to 70 days (0.70x). We produced 1100 successful simulations with at least 400 infected individuals and a median epidemic doubling time of 2.65 days (95% range: 1.50-4.10), and 2204 (66.7%) simulations failed to reach the epidemic threshold.

Sensitivity analysis–slower rate of infection. We simulated epidemics for a more slowly spreading virus using the same parameters from the primary analysis, except the transmission rate was decreased from 0.28 to 0.22 per day (0.79x) and the simulation time was increased from 100 to 150 days (1.50x). We produced 1100 successful simulations with at least 400 infected individuals and a median epidemic doubling time of 4.45 days (95% range: 1.50-7.44), and 7202 (92.8%) simulations failed to reach the epidemic threshold.

Sensitivity analysis–higher ascertainment rate. We simulated epidemics with a higher ascertainment rate using the same parameters from the primary analysis, except the ascertainment rate was increased from 0.15 to 0.25 (1.67x) and the simulation time was increased from 100 to 120 days (1.20x). Additionally, we decreased the transmission rate from 0.28 to 0.255 (0.91x) to keep the doubling time consistent with the primary analysis. We produced 1100 successful simulations with at least 400 infected individuals and a median epidemic doubling time of 3.52 days (95% range: 1.38-5.64), and 3698 (77.1%) simulations failed to reach the epidemic threshold.

Sensitivity analysis–lower ascertainment rate. We simulated epidemics with a lower ascertainment rate using the same parameters from the primary analysis, except the ascertainment rate was decreased from 0.15 to 0.05 (0.33x) and the simulation time was increased from 100 to 120 days (1.20x). Additionally, we increased the transmission rate from 0.28 to 0.295 (1.05x) to keep the doubling time consistent with the primary analysis. We produced 1100 successful simulations with at least 400 infected individuals and a median epidemic doubling time of 3.50 days (95% range: 1.51-5.65), and 4632 (80.8%) simulations failed to reach the epidemic threshold.

Determining stable coalescence. As in our previous study (*23*), we define the stable coalescence as the tMRCA that does not shift forward in time by more than one day, even as new individuals become infected and previously infected individuals recover (*i.e.*, the time to the most recent common ancestor [tMRCA]). The stable coalescence is reached the first day that the coalescence for the currently infected individuals is within one day of the time of MRCA after the simulation is complete or once 50,000 total individuals have been infected. Therefore, the stable coalescence ignores the preceding basal lineages that have gone extinct.

We extracted the tMRCA of infected and sampled individuals every day across each simulation using TreeSwift 1.1.14 (*90*). This tMRCA was calculated for each day of the 100 days or until 50,000 individuals had been infected, whichever came first. We chose not to explore dynamics after 50,000 infections due to a slowing in exponential growth arising from the saturation of the contact network.

Determining the topological patterns of simulated phylogenies. We examined the topology of the phylogenetic trees resulting from the epidemic simulations. For each tree, after determining the point of stable coalescence, we extracted the subtree rooted at the internal node at stable coalescence (*i.e.*, the MRCA of the sampled genomes). We then simulated mutations down the branches of the subtree using a substitution rate of $9x2x10^{-4}$ substitutions/site/year (the inferred substitution rate from the unconstrained Bayesian phylogenetic analysis). We then counted (i) the number of descendent lineages (*i.e.*, the size of the basal polytomy, including basal taxa, tips with unique mutations, and clades) from the subtree root, (ii) the clade size of each descendant lineage that was one or two mutations from the root, and (iii) the number of lineages descending from each one- or two-mutation derived clade from (ii) (*i.e.*, the size of the polytomy of the large derived clades). Note that (i) is represented in Fig. 2A (and the basal polytomy in Fig. 2C), and (ii) and (iii) describe the large clades in Fig. 2B and 2C. For clarity, we display the subtree rooted at stable coalescence (ignoring the preceding lineages that have gone extinct) in Fig. 2.

We use the results of i–iii above to quantify the frequencies of different topologies in the simulated epidemics (τ).

If there were at least *n* descendant lineages from the subtree root, the simulated phylogeny had a polytomy. We refer to this topology as τ_P.

When there were only two clades, each one mutation from the subtree root, the simulation matched the observed topology if there were a single introduction of the C/C or T/T ancestral haplotype. We refer to this topology as τ_{2C}. We first further constrained τ_{2C} to require each clade to be ≥30% and ≤70% of the simulated taxa, approximating lineage A and lineage B comprising 35.2% and 64.8% of all taxa, respectively. We constrained τ_{2C} once more to require at least *n* lineages descending from the root of the clade, to match the polytomies at the bases of the lineage A and lineage B clades.

When there was a polytomy at the base of the simulated phylogeny and a large clade two mutations from the root with a large portion of the taxa, the topology matches the observed topology had there been a single introduction of the lineage A or B ancestral haplotype. We refer to this topology as τ_{1C}. As with τ_{2C}, we constrain τ_{1C} to require the two-mutation clade to comprise ≥30% of the taxa. We constrained τ_{1C} once more to require at least *n* lineages descending from the large two-mutation clade (*i.e.,* a polytomy, as with lineage B if lineage A were the single introduction and vice-versa).

The empirical phylogeny has 787 taxa and two large polytomies: the 108-descendant lineage A polytomy and the 231-descendant lineage B polytomy. Our primary results therefore require the polytomy size *n* to be 100 lineages, reflecting the empirical data. Importantly, this is a conservative requirement because 98.4% of the simulated phylogenies had more than 1,000 taxa.

However, since 1.5% of the simulated phylogenies had fewer than 787 taxa, we also performed sensitivity analyses requiring *n* to be either 20 or 50 lineages. Additionally, since 96.7% of the simulated phylogenies had more than 5,000 taxa, we also performed sensitivity analyses requiring *n* to be 200 or 500 lineages.

Quantifying the support for two introductions against a single introduction of SARS-CoV-2. Here we synthesize the posterior probabilities of inferred ancestral haplotypes, frequencies of topologies in the forward epidemic simulations, and the expected relationships between these haplotypes and topologies to quantify the support for a two-introduction scenario over a single-introduction scenario. Our goal is to obtain $P(I_n \mid \mathbf{Y})$, the probability of *n* introductions (I_n) given our sequence data $\mathbf{Y}$, so that we can compare the probabilities of single- and multiple-introduction hypotheses.

We consider three phylogenetic topologies, or root-shapes: τ_P for a large polytomy at the root (stable coalescence), τ_{1C} for one well-defined clade two mutations from the root, and τ_{2C} for two well-defined clades each one mutation from the root, with no other lineages descending from the root. We define S_{MRCA} as the ancestral haplotype of all sampled SARS-CoV-2 genomes, and we specifically consider the four most likely ancestral haplotypes (Table 1): S_A for lineage A, S_B for lineage B, $S_{T/T}$ for the intermediate T/T haplotype, and $S_{C/C}$ for the intermediate C/C haplotype.

If there are *n* introductions, there are *n* distinct trees with *n* distinct root-shapes. We will use the vector $\boldsymbol{\tau}$ to track these. In the case that there was one introduction, this is a single root-shape; two introductions have two root-shapes; and we do not consider more than two introductions here.

We can rewrite the ratio of posterior probabilities as a ratio of joint probabilities,

$$\frac{P(I_2|\boldsymbol{Y})}{P(I_1|\boldsymbol{Y})} = \frac{P(I_2, \boldsymbol{Y})}{P(I_1, \boldsymbol{Y})}$$

Marginalizing over S_{MRCA} and assuming that I_n only affects **Y** through S_{MRCA},

$$\begin{aligned} P(I_n, \boldsymbol{Y}) &= \sum_{S_{MRCA}} P(I_n, \boldsymbol{Y}, S_{MRCA}) \\ &= \sum_{S_{MRCA}} P(\boldsymbol{Y}|I_n, S_{MRCA})P(I_n, S_{MRCA}) \\ &= \sum_{S_{MRCA}} P(\boldsymbol{Y}|S_{MRCA})P(I_n, S_{MRCA}) \end{aligned}$$

Therefore we get,

$$\begin{aligned} \frac{P(I_2, \boldsymbol{Y})}{P(I_1, \boldsymbol{Y})} &= \frac{\sum_{S_{MRCA}} P(\boldsymbol{Y}|S_{MRCA})P(I_2, S_{MRCA})}{\sum_{S_{MRCA}} P(\boldsymbol{Y}|S_{MRCA})P(I_1, S_{MRCA})} \\ &= \frac{\sum_{S_{MRCA}} \frac{P(S_{MRCA}|\mathbf{Y})P(\mathbf{Y})}{P(S_{MRCA})} P(I_2, S_{MRCA})}{\sum_{S_{MRCA}} \frac{P(S_{MRCA}|\mathbf{Y})P(\mathbf{Y})}{P(S_{MRCA})} P(I_1, S_{MRCA})} \\ &= \frac{\sum_{S_{MRCA}} \frac{P(S_{MRCA}|\mathbf{Y})}{P(S_{MRCA})} P(I_2, S_{MRCA})}{\sum_{S_{MRCA}} \frac{P(S_{MRCA}|\mathbf{Y})}{P(S_{MRCA})} P(I_1, S_{MRCA})} \end{aligned}$$

As we did when computing the Bayes Factors above, we assume that all ancestral haplotypes are *a priori* equally probable, leading to

$$\frac{P(I_2, \boldsymbol{Y})}{P(I_1, \boldsymbol{Y})} = \frac{\sum_{S_{MRCA}} P(S_{MRCA}|\boldsymbol{Y})P(I_2, S_{MRCA})}{\sum_{S_{MRCA}} P(S_{MRCA}|\boldsymbol{Y})P(I_1, S_{MRCA})} \tag{1}$$

The Bayesian phylogenetic inference provides P(S_{MRCA} | **Y**), the probability of the ancestral haplotype of all human sequences given the sequence data.

We can then marginalize the joint probability P(S_{MRCA}, I_n) over the root-shape vector and apply conditional probability:

$$
\begin{aligned}
P(S_{MRCA}, I_n) &= \sum_{\boldsymbol{\tau}} P(\boldsymbol{\tau}, S_{MRCA}, I_n) \\
&= \sum_{\boldsymbol{\tau}} P(S_{MRCA}|\boldsymbol{\tau}, I_n) P(\boldsymbol{\tau}, I_n)
\end{aligned}
$$

Since the length of $\boldsymbol{\tau}$ depends on the number of introductions, I_n does not provide any additional information regarding the conditional probability of S_{MRCA}. Therefore,

$$
\begin{aligned}
P(S_{MRCA}, I_n) &= \sum_{\boldsymbol{\tau}} P(S_{MRCA}|\boldsymbol{\tau}) P(\boldsymbol{\tau}, I_n) \\
&= \sum_{\boldsymbol{\tau}} P(S_{MRCA}|\boldsymbol{\tau}) P(\boldsymbol{\tau}|I_n) P(I_n)
\end{aligned}
$$

The maximum likelihood tree (Fig. 1), likely ancestral haplotypes (Table 1), and forward epidemic simulations (Fig. 2) allow for the following compatibility statements:

- $\boldsymbol{\tau} = \tau_{2\mathrm{C}}$ is compatible with $S_{MRCA} \in \{S_{C/C}, \mathrm{S}_{T/T}\}$
- $\boldsymbol{\tau} = \tau_{1\mathrm{C}}$ is compatible with $S_{MRCA} \in \{S_A, \mathrm{S}_B\}$
- $\boldsymbol{\tau} = (\tau_{\mathrm{P}}, \tau_{\mathrm{P}})$ is compatible with $S_{MRCA} \in \{S_A, \mathrm{S}_B, S_{C/C}, \mathrm{S}_{T/T}\}$
- $\boldsymbol{\tau} = (\tau_{\mathrm{P}}, \tau_{1\mathrm{C}})$ is compatible with $S_{MRCA} \in \{S_A, \mathrm{S}_B, S_{C/C}, \mathrm{S}_{T/T}\}$
- $\boldsymbol{\tau} = (\tau_{1\mathrm{C}}, \tau_{\mathrm{P}})$ is compatible with $S_{MRCA} \in \{S_A, \mathrm{S}_B, S_{C/C}, \mathrm{S}_{T/T}\}$
- $\boldsymbol{\tau} = (\tau_{1\mathrm{C}}, \tau_{1\mathrm{C}})$ is compatible with $S_{MRCA} \in \{S_A, \mathrm{S}_B, S_{C/C}, \mathrm{S}_{T/T}\}$

We can then define P(S_{MRCA} | $\boldsymbol{\tau}$) to be proportional to the vector of compatibilities:

$$
P(S_{MRCA}|\boldsymbol{\tau}) \propto \begin{cases} 1 & \text{if } \boldsymbol{\tau} \text{ and } S_{MRCA} \text{ are compatible} \\ 0 & \text{otherwise} \end{cases}
$$

In the case where multiple root-shapes are compatible with an ancestral haplotype, we must renormalize the compatibility vector such that it sums to 1 to describe probabilities. However, note that some root-shapes are not compatible with any ancestral haplotypes. In this case, P(S_{MRCA} | $\boldsymbol{\tau}$) equals 0 for each ancestral haplotype.

The simulations provide P($\boldsymbol{\tau}$ | I_1), the probability of the root-shape(s) given one introduction. We assume each introduction is independent, allowing us to generalize this probability to P($\boldsymbol{\tau}$ | I_n). For example, P($\boldsymbol{\tau} = \tau_{\mathrm{P}}$ | $I_n = I_1$) = 0.607 (Fig. 2, Table S5), and P($\boldsymbol{\tau} = (\tau_{\mathrm{P}}, \tau_{\mathrm{P}})$ | $I_n = I_2$) = P($\boldsymbol{\tau} = \tau_{\mathrm{P}}$ | $I_n = I_1)^2$ = 0.368.

Lastly, we assume equal prior probabilities for one and two introductions of SARS-CoV-2, allowing us to cancel out P(I_1) and P(I_2) when calculating equation (1). We can then use P(S_{MRCA} | **Y**), P(S_{MRCA} | $\boldsymbol{\tau}$), and P($\boldsymbol{\tau}$ | I_1) to calculate the posterior odds of equation (1). Although we solved for the posterior odds in support of two introductions, the prior odds comparing two introductions to a single

introduction is 1. Therefore, the posterior odds are equivalent to a Bayes factor in support of two introductions. The code used to calculate the Bayes factor comparing two introductions to a single introduction is available at (*67*).

Combining epidemic simulations and BEAST via rejection sampling. We estimated the timing of the lineage B and lineage A primary cases. Our previous analysis (*23*) inferring the timing of the SARS-CoV-2 primary case incorporated the date of index case ascertainment; however, uncertainty regarding the true index case persists (*8*, *34, 91*) (supplementary text). To overcome this uncertainty, we extend our previously published approach, which combines the epidemic simulations and phylodynamics tMRCA inference (described in the above two sections), to condition the timing of the primary case on both the index case symptom onset date and earliest documented COVID-19 hospitalization date. We included hospitalization dates because they are less susceptible to recall bias than date of symptom onset.

Our aim is to obtain a posterior distribution for the date X of the primary case (the first case resulting from a SARS-CoV-2 cross-species transmission) in Wuhan, conditioned on the available sequencing data D_S, the date of the first reported COVID-19 case D_C, and the date of the first hospitalization D_H due to COVID-19. We do this in a Bayesian framework by marginalizing over the date T of the tMRCA as follows:

$$P(X|D_S, D_C, D_H) = \int_T P(X|T, D_S, D_C, D_H)P(T|D_S, D_C, D_H)dT \quad (2)$$

We assume that the sequencing data are informative only for the tMRCA; *i.e.*, given T, X does not depend on D_S: $P(X|T, D_S, D_C, D_H) = P(X|T, D_C, D_H)$. We also assume that the first reported COVID-19 case and hospitalization data are not informative for the tMRCA: $P(T|D_s, D_c, D_H) = P(T|D_s)$. This gives:

$$P(X|D_S, D_C, D_H) = \int_T P(X|T, D_C, D_H)P(T|D_S)dT \quad (3)$$

We further note that

$$P(X|T, D_C, D_H) = \int_{Y_H}\int_{Y_C} P(X, Y_C, Y_H|T, D_C, D_H)dY_C dY_H$$

where Y_C and Y_H are the first simulated COVID-19 ascertained case and hospitalization, respectively. We model $P(X, Y_C, Y_H|T, D_C, D_H)$ as proportional to $I(Y_C \leq D_C, Y_H \leq D_H)P(X, Y_C, Y_H|T)$, where $I(Y_C \leq D_C, Y_H \leq D_H)$ is an indicator function with a value of 1 when Y_C and Y_H are consistent with D_C and D_H, respectively, and 0 otherwise. This approach allows us to sample from the posterior

distribution of equation (3). The BEAST analysis provides values of T sampled from the distribution $P(T|D_S)$, which we can use in conjunction with FAVITES-COVID-Lite to sample corresponding values of X, Y_C, and Y_H from the distribution $P(X, Y_C, Y_H|T)$. We use a simple rejection sampling approach to continue sampling from $P(X, Y_C, Y_H|T)$ until a sample is obtained for which $I(Y_C \leq D_C\ , Y_H \leq D_H) = 1$. The resulting set of sample values for X then follow the posterior distribution $P(X|D_S, D_C, D_H)$.

We require the first simulated case to be ascertained (SAPHIRE stage: I) and assign D_C as 10 December 2019. However, we note that this first ascertained case can be the primary case, unless a secondary or tertiary case progresses faster through the course of infection. We assign D_H as 16 December 2019. Importantly, the rate at which cases were ascertained in the SAPHIRE model is based on real-time patterns in COVID-19 diagnosis from 1 through 22 January 2020 and may not reflect the actions that led to the retrospective diagnosis of earliest cases of COVID-19. Further, stable coalescence (*i.e.*, the MRCA) can happen any time after the primary case is infected, and there is no requirement for stable coalescence to occur after the first ascertained and unascertained individuals. Justifications for dates used here and in the sensitivity analyses is discussed in the supplementary text.

Sensitivity analysis–earliest case date of 8 December. We also condition single-introduction analyses on an 8 December case date, which was previously discounted by the WHO and 16 December hospitalization date (see supplementary text for full discussion).

Sensitivity analysis–rejection sampling with hospitalization only. We remove the requirement for the first simulated case to be ascertained by a given date, and then condition analyses only on the tMRCA and date of the first hospitalization.

Sensitivity analysis–recCA and constrained roots. We explored the sensitivity of the timing of the primary case to the phylodynamic model choice. We applied rejection sampling to the inferred phylogenies constrained by the recCA and SARS-CoV-2 ancestral haplotypes (*e.g.*, lineage A.1) and the primary forward epidemic simulations, conditioning on the same dates as above.

Rejection sampling for lineages A and B. We apply the above method to the tMRCA, first ascertained case date, and first hospitalized case date for each lineage to infer the timing of the primary case for each lineage. For lineage B, the earliest case and hospitalization dates are 13 December and 16 December, respectively. For lineage A, the earliest case date is 15 December and the earliest hospitalization date is 25 December (see supplementary text for full discussion). After performing rejection sampling for both lineages, we combine the number of individuals in each compartment for each day in the dated simulations.

Sensitivity analysis—earlier lineage B COVID-19 index case dates. We performed rejection sampling for lineage B using a case date and hospitalization date of 10 December and 16 December, matching the SARS-CoV-2 index case, which does not have an associated genome. We also performed rejection sampling for lineage B using a case date and hospitalization date of 8 December and 18 December,

respectively, under the alternate assumption that the earliest lineage B case occurred on 8 December (see supplementary text for full discussion).

Simulating cross-species transmissions to achieve two successful introductions. Our epidemic simulations had a success rate of approximately 22.2% (1100 successful introductions; 3857 failed introductions). To simulate the number of cross-species transmissions needed to achieve two successful introductions, we treated successful introductions as Bernoulli trials, with a success rate of 22.2% and simulated trials until there were two successful trials.

Supplementary Text

Reduced genomic diversity in the early pandemic. Although the root of the SARS-CoV-2 phylogeny has often been inferred with Bayesian and maximum likelihood methods to fall on the branch leading to IPBCAMS-WH-01 (Lineage B) or other early genomes (*18*, *23*, *35*, *92*, *93*), reanalysis of sequence data from the earliest sampled viruses found that three previously reported mutations in IPBCAMS-WH-01 were spurious, and the genome was, in fact, identical to the Hu-1 reference genome (*34*). Upon reexamination by WHO investigators, other early genomes were also found to have spurious mutations (*34*), thereby decreasing the overall genetic diversity of early SARS-CoV-2 sequence data. This decreased diversity suggests that prior studies, including our own (*23*), may have incorrectly rooted the SARS-CoV-2 phylogeny.

C/C and T/T genomes through 14 February 2020. Of the 16 C/C genomes in our data set, we found four that share nucleotide substitutions—other than T28144C—found in some lineage A genomes (Fig. S1A). If the C/C intermediates actually existed, 11 of the 19 additional unique mutations in the C/C genomes would need to be homoplasies: identical mutations arising separately in the C/C and lineage A genomes. For example, a C/C genome from Anhui province (EPI_ISL_1069206) shares A11430G with 6 lineage A genomes sampled across China, and a genome from Sichuan province (EPI_ISL_451325) shares C1342T and C18060T with three lineage A genomes. The authors of the latter example confirmed that low sequencing depth at position 8782 led to the erroneous calling of the reference genome nucleotide at this position in this genome (L. Chen Personal Communication). Furthermore, these authors confirmed that incorrect base calls, often due to low sequencing depth, led to erroneous assignment of 11 additional C/C genomes sampled in Wuhan and Sichuan province (four of which share substitutions with lineage B genomes, see below).

A similar pattern was observed in the five C/C genomes sharing substitutions found within lineage B (Fig. S1B), including a South Korean genome (EPI_ISL_413017) sharing G26640T, G26144T, and T26677C with another lineage B genome from South Korea. In this instance, we confirmed that low sequencing depth at position 28144 (<10x) resulted in this erroneous assignment. Critically, therefore, we are able to explain all C/C genomes as artifactual, with the exception of two genomes sampled in Beijing in late January and early February, whose additional mutations were not observed in early lineage A or B genomes and whose underlying data was not available.

Unlike the C/C genomes, none of the four T/T genomes shared additional mutations with lineage A or B genomes that would clarify their veracity. However, we confirmed that the T/T genome sampled in Singapore on 14 February 2020 (EPI_ISL_462306) had low coverage at both 8782 and 28144 (≤10x). Moreover, the 3 T/T genomes sampled in Wuhan on 26 January (EPI_ISL_493179, EPI_ISL_493182, EPI_ISL_493180) had low sequencing depth and indeterminate C/T nucleotide assignment at position 8782 (Table S1). These findings suggest all T/T genomes sampled by 14 February 2020 are similarly artifactual.

T/T genomes aboard the Diamond Princess. Two high coverage T/T genomes sampled after 14 February 2020 were from the Diamond Princess cruise ship outbreak. These two T/T genomes

possessed T11083G, a mutation defining the Diamond Princess outbreak, as well as G11410T, and were therefore identifiably descendants of the lineage B virus that initiated this outbreak (*94, 95*) (Fig. S2).

T/T genomes in New York City. We found 3 SARS-CoV-2 genomes with both C8782T and T28144 (T/T) in the NYC Public Health Laboratory surveillance data set. We placed these genomes on a global tree of 3 million genomes (v2022-01-21) using UShER (*96*), and all 3 appear to be descendants of lineage B. Two of these genomes (EPI_ISL_8953704 and EPI_ISL_8953705) belong to the B.1.526 *Iota* lineage and have identical sequences, suggesting that a T/T genome may have been transmitted locally (Fig. S3). We note that although these genomes were sampled from different individuals in different parts of the city, their genomes were sequenced on the same sequencing plate. The third NYC T/T genome (EPI_ISL_1447116) belongs to the B.1.2 lineage and falls on a different part of the phylogeny, indicating an independent C8782T mutation. This third genome was sequenced on a separate run than the other two NYC T/T genomes. All three genomes at site 8782 and 28144 have read depth >4,000x, with coverage in both directions and 100% of the sequencing reads support the T allele.

T/T and C/C genomes in San Diego. We found 24 T/T genomes in the San Diego SEARCH data set, with collection dates between December 2020 and December 2021 and 1 C/C genome that was collected on 15 January 2021. We placed these genomes on a global tree of 3 million genomes using UShER. Eight of the T/T genomes were classified as Delta sublineages: AY.26, AY.40, and AY.44; the remaining T/T genomes were classified as B.1 and its other descendant lineages (Fig. S4). The C/C genome was classified as B.1.1.432 (Fig. S5). Therefore, these and other T/T and C/C haplotypes are the result of convergent evolution at 8782 and 28144.

The common occurrence of C/C and T/T genomes arising due to convergence provides further evidence that early intermediate genomes could have also been due to convergent evolution and do not represent transitional genomes.

The recombinant common ancestor (recCA). The recCA differed from Hu-1 by just 381 reversions, including C8782T and T28144C. In this manner, lineage A (exemplified by Wuhan/WH04/2020) has two reversions, and hence has two fewer substitutions separating it from the recCA than lineage B. (Note that although these mutations are nominally ‘reversions’, if the true MRCA of SARS-CoV-2 were a lineage A virus, those lineage B to lineage A reversions would not actually have occurred). Additional reversions, C18060T and C29095T, have been separately identified in USA/WA1/2020 and Guangdong/20SF012/2020, respectively, and it has been argued that these haplotypes are the ancestral form of SARS-CoV-2 (*19*, *21*). We find that repeated substitutions at sites 8782, 18060, and 28144, are common among closely related sarbecoviruses (Fig. S11-S13). In contrast, 29095 is strongly conserved among these sarbecoviruses but highly polymorphic in humans (Fig. S14). Absent from the recCA are two mutations, C2416T and C23929T, previously suggested to have been present in the immediate ancestor of SARS-CoV-2 (*21*); these mutations occur on the branch to the related bat sarbecovirus RaTG13 (Fig. S8, S10) (*97*).

To ensure our downstream analyses are robust to the breakpoint inference and subsequent construction of the recCA, we reconstructed a second recCA using breakpoints 3SEQ, a recombination detection algorithm which examines triplets (two parents and one offspring sequence) (*76*). We then constructed this second recCA with the 21 new breakpoints, and this recCA differed from Hu-1 at 371 sites. The two recCAs were identical at sites previously suggested to have mutations present in the immediate ancestor (*i.e.*, 2416, 8782, 18060, 19524, 23929, 28144, and 29095).

Justification for a non-reversible substitution model. We developed a random-effects non-reversible substitution model for our phylogenetic inference because of the substantial C-to-T transition bias (*15–17*) and frequent C-to-T reversions (described in Main Text). To compare the ancestral haplotype inference between the random-effects model and a standard reversible substitution model, we performed Bayesian phylodynamic inference with a GTR substitution model with both the unconstrained and recCA-rooted analyses. We find that C/C and T/T ancestral haplotypes were less common under the GTR model than the random-effects model (Data S3). Notably, the difference in posterior support for C/C and T/T ancestral haplotypes was negligible under the unconstrained GTR model, indicating the increased level of biological realism reflected in the random-effects substitution model inference. Importantly, ancestral haplotypes such as A.1 (BF>150) and A+C29095T (BF>50) were poorly supported under the GTR model, just as in the unconstrained and the recCA random-effects model.

Sensitivity analyses of ancestral haplotype inference with unconstrained rooting. We extended the analysis that excluded the 15 market-associated genomes to account for any potential ascertainment bias of early sampling, particularly of lineage B, by excluding all 96 genomes from Wuhan (59 lineage B, 37 lineage A). Upon excluding these genomes from Wuhan, the posterior support for a lineage B ancestral haplotype decreased, while the support for C/C and lineage A ancestral haplotypes increased, with all other lineages still unsupported (Data S3). Therefore, lineage A is only a plausible ancestral haplotype (BF<10) of SARS-CoV-2 under the unconstrained model if we do not include all the early genomes from Wuhan, which represent a substantial portion of the early genomic diversity.

To understand the impact of excluding the C/C and T/T "intermediate" genomes, we performed Bayesian phylogenetic inference of SARS-CoV-2 with the original 787 genomes (see Methods) and the 20 excluded intermediate genomes, with unconstrained rooting. When including all 20 "intermediate" genomes—16 C/C and 4 T/T genomes—the results were similar to the main unconstrained analysis (Data S3). These results were also consistent when we included only the C/C or the T/T genomes separately, indicating that the posterior support for a C/C or T/T ancestral haplotype does not increase when including intermediates, even if only including genomes with just one of the two intermediate ancestral haplotypes (Data S3).

When we included the previously masked 388 genomic sites for ancestral haplotype inference of the 787 genomes (*i.e.*, an unmasked alignment), our results were consistent with our primary masked unconstrained rooting approach (Data S3).

Ancestral haplotype inference with outgroup rooting. We used outgroup rooting with individual bat sarbecoviruses as a comparison for the recCA rooting inferences. We performed the Bayesian inference

with either RaTG13 or BANAL-20-52 as an outgroup. Relative to the recCA rooting results, posterior support for a lineage B and C/C ancestral haplotypes increased, whereas the support for the lineage A ancestral haplotype decreased (Data S3). All other previously suggested or inferred haplotypes, including lineage A.1, were rejected, consistent with the other rooting approaches (Data S3).

These results indicate that decreasing the branch length from the MRCA of SARS-CoV-2 and its ancestor (*i.e.*, the MRCA of SARS-CoV-2 and the outgroup, or the recCA) increases support for lineage A as the ancestral haplotype (when the recCA and the outgroup are identical at key sites—here, 8782 and 28144). These findings indicate rooting with the recCA as opposed to an outgroup can help account for the effects of rate variation along the long branch leading to SARS-CoV-2.

The tMCRA of SARS-CoV-2 is consistent across explored ancestral haplotypes. It has been suggested that a phylogenetic root in lineage A would produce older tMRCA estimates than a lineage B rooting (*21*). However, we find that SARS-CoV-2 tMRCA inference is generally robust to the rooting model and ancestral haplotype.

The unconstrained rooting (Fig. S29A), which favored a lineage B ancestral haplotype (Table S2), and produced a median tMRCA of 11 December 2019 [95% highest posterior density (HPD): 25 November to 20 December] and a mean substitution rate of 9.2×10^{-4} substitutions/site/year (95% HPD: 8.1×10^{-4} to 1.0×10^{-3}). These tMRCA estimates are similar to our previous inference (*23*), although the substitution is slightly faster. This elevated rate is expected, given that shorter sampling windows are associated with the inference of a more rapid substitution rate in SARS-CoV-2 (*23*, *98*, *99*). The recCA-constrained rooting (Fig. S18C), which favored a lineage A ancestral haplotype (Table 1), produced a median tMRCA of 6 December 2019 (95% HPD: 15 November to 19 December) and a mean substitution rate of 9.2×10^{-4} substitutions/site/year (95% HPD: 8.0×10^{-4} to 1.0×10^{-3}).

To explicitly explore the effect of ancestral haplotype on the SARS-CoV-2 tMRCA, we employed our novel phylodynamic framework that fixes the MRCA of the SARS-CoV-2 phylogeny to ancestral haplotypes (Fig. S18D, see methods), rather than using sampled taxon (*e.g.*, Hu-1), an outgroup (*e.g.*, RaTG13), or their inferred ancestor (*e.g.*, recCA). We explored the plausible ancestral haplotypes (lineage A, lineage B, and C/C), as well A.1 (WA1) and A.1 + C29095T (20SF012) (see methods). The resulting tMRCAs were consistent across the ancestral haplotypes (Table S2), indicating the ancestral haplotype has minimal impact on tMRCA inference.

The tMRCA of lineage B predates the tMRCA of lineage A when excluding market-associated genomes. We considered the possibility that the predominance of lineage B viruses in the beginning of the pandemic, particularly at the Huanan market, was biasing the earlier inference of the lineage B tMRCA. However, when we excluded all market-associated genomes, the median tMRCA of lineage B was 17 December (95% HPD: 29 November to 26 December), still earlier than the median tMRCA of lineage A: 25 December (95% HPD: 15 December to 4 January). Therefore, the predominance of lineage B at the Huanan market is not biasing its tMRCA to predate the tMRCA of lineage A (Table S2).

Doubling time of the early pandemic. To properly parameterize the epidemic simulations, we characterized the epidemic growth dynamics of the early pandemic. We first extracted the daily case counts from the WHO report for December 2019 (*34*) and then adjusted the case counts reported in Li et al. (*80*) to properly reflect the earliest cases identified (Fig. S21A; see Methods). We used EpiNow2 (*81*) to calculate the daily doubling time of SARS-CoV-2 through mid-January 2020, which resulted in a median doubling time of SARS-CoV-2 and 50% HPD consistently below 5 days through the end of December 2019 (Fig. S21B). On 15 December 2019, five days after the earliest known COVID-19 case, the median doubling time was 3.74 days (50% HPD: 3.37–4.24; 95% HPD: 2.80–5.43). However, we are interested in the epidemic dynamics prior to the discovery of the earliest COVID-19 cases and the identification of SARS-CoV-2 as the etiological agent of what was then a pneumonia of unknown etiology, when viral spread was least inhibited by human behavior.

The inferred doubling time is stochastic and dependent on which day it is estimated (Fig. S21B); it is therefore imprecise to report a single doubling time when describing general epidemic dynamics. In our simulations, we measure doubling time in four ways (Fig. S22): (i) cumulative doubling time since the start of the simulation, (ii) 14-day doubling time from day 14 until the end of the simulation, with cumulative doubling time reported prior to day 14, (iii) cumulative doubling time once a certain number of individuals are infected (*e.g.*, the cumulative doubling time at the 100th infection), and (iv) 14-day doubling time once a certain number of individuals are infected, with cumulative doubling time reported if that number of infections occurred before day 14 in the simulation. The 50% and 95% highest density intervals (HDIs) of the doubling time across simulations at cumulative infection counts [(iii) and (iv) above] narrows as more individuals are infected in the simulations (Fig. S22, bottom panels), although HDIs of the doubling time reported daily [(i) and (ii) above] in the simulation (Fig. S22, top panels) remain wide.

These results suggest doubling time based on the number of individuals infected [(iii) and (iv) above] is a better metric for understanding simulation growth dynamics than daily doubling times [(i) and (ii) above], because the simulations will typically be at a similar "point" in the epidemic once they reach a similar number of infections. For example, the 14-day doubling time at the thousandth case in each simulation has a narrower HDI than the 14-day doubling time reported on the same day across each simulation, regardless of the day. We therefore focus on the doubling time once a specific number of infections has been reached, and as doubling time is proportional to the epidemic speed (*100*) at a specific time point, we report the 14-day doubling time. Specifically, we report the 14-day doubling time once there have been one thousand infections, as the epidemic network has not yet been saturated, but the doubling time has a narrower HDI than earlier in the simulations.

We note that although the doubling time of SARS-CoV-2 was initially reported to be longer than 5 days (*101*, *102*), the case counts of the early pandemic have become more thorough (*80*), particularly with the release of the WHO report (*34*). A subsequent analysis of the doubling time in Wuhan used high resolution travel data and early case reports outside of Hubei province to avoid potential biases in reporting and case confirmation in Wuhan; their inferred doubling time was below 3.5 days (*26*). Additionally, doubling times reported for well-characterized early outbreaks outside of China, before

widespread non-pharmaceutical interventions were implemented, were often below four days (*27*, *103*, *104*).

Following from the empirical doubling times, we parameterize our primary simulations to have a median doubling time of 3.47 days (50% HDI: 2.71–4.15; 95% HDI: 1.35–5.44), slightly shorter than the inferred doubling time on 15 December 2019. We additionally perform sensitivity analyses with simulation parameterization resulting in a median doubling time of 2.65 days (50% HDI: 2.04–2.96; 95% HDI: 1.50–4.10) and 4.45 days (50% HDI: 3.30–5.35; 95% HDI: 1.50–7.44).

Importantly, the doubling times of the simulated data indicate that it is possible to infer a doubling time of greater than 5 days, even when the underlying parameters typically produce a shorter doubling time (Fig. S22). Similarly, our empirical doubling time estimates (Fig. S21) and the results from the literature suggest that it is possible to infer a doubling time of greater than 5 days (*101*, *105*) even when the underlying biology would typically produce a shorter doubling time (*26*) (Fig. S22), especially if cases are particularly undersampled. Coupled with our results indicating the doubling time was likely below four days in the early pandemic in Wuhan, these analyses of epidemic doubling times both in and outside of Wuhan suggest our simulations likely capture the doubling time of SARS-CoV-2 before its identification as the etiological agent of COVID-19.

Ascertainment rate of the early pandemic and parameterizing the simulations. Our epidemic simulations use an ascertainment rate of 15% (every 15/100 infections are ascertained) based on Hao et al. (*64*). Their ascertainment rate was informed by confirmed cases exported from Wuhan to Singapore. This ascertainment rate falls in the interquartile range (12.7–35.8%) reported in Chinazzi et al. (*106*) and is close to the 14% reported in Li et al. (*107*).

However, some of the earliest inferred ascertainment rates were lower: Wu et al. (*101*) calculate an ascertainment rate of 1.8% (0.9–3.3%). This value was likely reflective of the data available at the time: Wu et al. only reported 44 confirmed cases by 3 January 2020. However, more complete data from Li et al. (*80*) reports 392 confirmed infections by 3 January 2020. With 392 confirmed infections, a crude calculation leads to an ascertainment rate approximately 10-fold higher: 392/(44/0.018) = 16.0%. Therefore, some of the earliest estimations of ascertainment rates were likely too low for our simulation parameterization because we base our simulations on inferences of more recent and complete data (*34*, *80*).

Nonetheless, as there are lower and higher ascertainment rates reported in the literature describing the early pandemic, we performed sensitivity analyses of our epidemic simulations using ascertainment rates of 5% and 25%. When doing so, we slightly adjusted the transmission factor for the simulations to keep the doubling time centered on 3.5 days. Specifically, we performed one set of simulations with an ascertainment rate of 5% and median doubling time of 3.52 days (50% HDI: 2.71–4.22; 95% HDI: 1.38–5.64), and another set of simulations with an ascertainment rate of 25% and median doubling time of 3.50 days (50% HDI: 2.78–4.16; 95% HDI: 1.51–5.65).

Synthesizing evidence for multiple introductions of SARS-CoV-2. Both lineages A and B are characterized by large polytomies: many sampled lineages descending from a single node on the

phylogenetic tree. There are 108 and 231 lineages (including basal taxa) descendent from the base of lineages A and B, respectively (Fig. 1). To match the empirical data, we first examined the simulations for polytomies with at least 100 descendant lineages, and these were present in 47.5% of the simulated epidemics when the doubling time is 3.47 days (95% HDI: 1.35–5.44). (Fig. 2B). However, this is, in fact, a conservative estimate of the polytomy size in our simulated phylogenies, because 98.4% of these simulated phylogenies had more than 1,000 taxa.

If C/C is the ancestral haplotype, then SARS-CoV-2 is characterized by two clades: lineages A and B, each one mutation from the root with no transitional genomes (Fig. 2A). This topology, where there are only two clades of any size, each one mutation from the root, was present in 10.5% of phylogenies from our simulated epidemics. However, both lineages A and B are large clades, comprising 35.2% and 64.8% of the early SARS-CoV-2 genomes, respectively, and the smaller clade in these simulations was rarely this large. If we require our simulated clades to more realistically comprise at least 1% of the taxa, only 6.7% of the simulations match the C/C topology. If we require both clades to comprise ≥30% of the taxa—better reflecting empirical genomic diversity—only 1.5% of the simulations match the C/C topology. Finally, both lineages A and B comprise large polyomties. When we require each of these clades to have a basal polytomy of at least 100 descendant lineages—a conservative reflection of the 108- and 231-lineage polytomies characterizing lineages A and B, respectively—none of the simulations still match the C/C topology. These results indicate that a single introduction of C/C virus would not be expected to give rise to lineages A and B with no surviving ancestral C/C lineages.

If lineage A or B is the ancestral haplotype, then SARS-CoV-2 is characterized by a large basal polytomy with the largest clade in the tree separated by two mutations from the root (lineage B is the descendant clade if lineage A is the root, and vice-versa) (Fig. 2C). Importantly, our simulations permit these two mutations to occur either within a single individual or during successive infected hosts (*108*), reflective of multiple mutations of SARS-CoV-2 occurring within the serial interval between transmission partners (*109*). We see a large clade comprising a substantial fraction of the sampled taxa (*i.e.*, between 30% and 70%, reflecting either lineage A or B prevalence) in 10.8% of the epidemic simulations. When we require the large clade separated by at least two mutations from the basal polytomy of at least 100 descendant lineages, we observe this topology in 4.1% of epidemic simulations (Fig. 2C). However, if we also require the large clade to have at least a 100-lineage polytomy at its base, only 0.5% of the simulations match the topology if there were a single introduction of lineage A or B without any surviving transitional C/C lineages.

We then quantified the support for two introductions versus a single introduction resulting in the observed phylogeny: two large polytomies separated by two mutations. There was strong support for two introductions with our primary analysis (BF=61.6 and BF=60.0 with the recCA and unconstrained rooting, respectively; see Methods), as well as with sensitivity analyses with varying transmission and ascertainment rates (Table S5). We observe that sensitivity analyses with longer doubling times increase the support for multiple introductions (Table S5). Although multiple mutations in a short transmission chain (and therefore between internal nodes in a phylogeny) are more likely to occur with a longer doubling time, there is a reduction in the probability of observing large polytomies occurring

in rapid succession as the length of the doubling time increases. Therefore, an even longer doubling time would be less likely to produce the empirical topology.

Importantly, since 96.7% of the simulated phylogenies had more than 5,000 taxa, our primary analysis requiring polytomies to have at least 100 descendant lineages is quite conservative. We therefore performed sensitivity analyses requiring the polytomies to have either 200 or 500 descendant lineages. These increased polytomy sizes are more realistic when considering the size of the lineage A polytomy (108 descending lineages) relative to the empirical phylogeny (787 taxa). However, both polytomy sizes are still proportionally less than the 108 descendant lineages of lineage A from our empirical 787-taxa phylogeny and are therefore still conservative. Regardless, the support for two introductions increased with both of these analyses (Table S5), again indicating the rarity of two polytomies occurring in rapid succession.

Additionally, since 1.5% of the simulated phylogenies had fewer than 787 taxa, we also performed sensitivity analyses requiring the polytomies to have either 20 or 50 descendant lineages. Regardless of the polytomy size we conditioned on, there was still support for two introductions (Table S5).

In sum, there is consistent support for two introductions of SARS-CoV-2 across multiple rooting constraints, longer and shorter doubling times, higher and lower ascertainment rates, and increased and decreased minimum polytomy sizes. Therefore, two introductions, rather than a single introduction, of SARS-CoV-2 are more likely to have produced the two polytomies near the base of the SARS-CoV-2 phylogeny.

Index case dates of SARS-CoV-2. The date of the index case informs our understanding of when the pandemic began, and we use this date when inferring the time of the primary case. An early report suggested an index case (*i.e.*, first identified case) dating to 17 November 2019, with at least one case being reported each day thereafter (*110*). A different report suggested an index case date of 1 December 2019 (*102*). However, the World Health Organization (WHO)-China report did not find evidence to support the veracity of these cases and identified the earliest case as having an illness onset of 8 December (case S01 from Table 6 in the WHO report) (*34*). A subsequent review of the earliest COVID-19 cases suggested that the '8 December' patient actually became ill on 16 December and concluded that the index case was a vendor from the Huanan Seafood Market who became ill on 10 December and was hospitalized on 16 December (*8*). This shift in index case dates necessitates reexamining the timing of the primary case (*i.e.*, the first human infected with a pathogen in an outbreak) of SARS-CoV-2, as case data is crucial to timing the first SARS-CoV-2 infection (*23*).

When we perform rejection sampling to infer the date of the primary cases of lineages A and B separately, we use case and hospitalization dates associated with each lineage. The SARS-CoV-2 index case from 10 December does not have an associated published genome. However, every genome associated with the Huanan market and collected before 30 December was lineage B. Additionally, an environmental sample from the stall this vendor operated (EPI_ISL_408512) was also lineage B. We

therefore assume this individual likely had a lineage B virus, and we used their illness onset and hospitalization dates to time the primary case of lineage B.

The earliest case and hospitalization dates for lineage A belong to 'Cluster 1' from the WHO report (Annex E2) (*34*). The lineage A genome belongs to the individual in the cluster with illness onset on 26 December (case S13 from Table 6 in the WHO report; IME-WH01; GISAID accession EPI_ISL_529213). However, the spouse of this individual was infected as well, becoming symptomatic on 15 December and hospitalized on 25 December. We can therefore reasonably assume the spouse was also infected with a lineage A virus and subsequently infected the earliest confirmed lineage A case. We thus use the dates belonging to the spouse when timing the primary case of lineage A.

Robustness of lineage B primary case inference to date of index case. The earliest lineage B case with a published genome (IPBCAMS-WH-01; GISAID accession EPI_ISL_402123; case S02 from Table 6 in the WHO report) became symptomatic and was hospitalized on 13 and 18 December, respectively (*2*). Although this individual was initially reported to have an illness onset date of 15 December, the WHO report subsequently determined he had an earlier onset of 13 December (*34*). The timing of the lineage B primary case is robust to these later symptom onset and hospitalization dates, occurring on 21 November (95% HPD: 25 October–11 December) when rooting with recCA.

Because the '8 December' patient was lineage B (*34*), we examined the effect of conditioning on 8 December as an index case date. The timing of the lineage B primary case was robust to this earlier index case date (Table S7), occurring on 17 November (95% HPD: 22 October to 6 December) when rooting with recCA. Therefore, even if the 8 December case were the index case, the first lineage B SARS-CoV-2 virus still likely jumped into humans at a time similar to our inference with the 10 December index case date.

Epidemiological dynamics of a single SARS-CoV-2 introduction scenario. As a counterfactual scenario, we examined the timing of the primary case if SARS-CoV-2 were the result of only a single introduction. Here, we condition on the tMRCA of all SARS-CoV-2 (Table S2) and the ascertainment (10 December) and hospitalization (16 December) dates belonging to the SARS-CoV-2 index case (the seafood vendor). The timing of the primary case in a single introduction scenario is similar to that of lineage B (Table S7, S14), even when using lineage A.1 or lineage A + C29095T as the ancestral haplotype, and is also robust to higher and lower ascertainment rates (Table S15). The number of infections and hospitalizations at specific dates resulting from a single introduction are similar to the combined infections and hospitalizations from lineages A and B (Fig. 4A, S24A, S29; Table S11, S12), even when using the ancestral haplotypes producing the oldest tMRCA estimates (lineage A.1 and lineage A + C29095T). These results indicate that there was likely not substantial cryptic spread even if lineage A.1 or A + C29095T were the sole introduction of SARS-CoV-2, and that standard epidemic modeling that does not account for phylogenetics will not be able to distinguish the number of introductions based on case-counts or hospitalizations alone.

Consistent timing of the primary case when conditioning only on hospitalization. Due to the controversy that remains about the true SARS-CoV-2 index case (*8*), we performed sensitivity analyses on our primary case timing by conditioning just on the tMRCA and the date of the earliest hospitalization. The timing of the primary case was robust to the exclusion of the index case date in our rejection sampling approach for both single- and multi-introduction scenarios, indicating that index case dates did not bias our results toward earlier dates (Table S7, S9, S14). Had there been extensive cryptic spread of SARS-CoV-2 in Wuhan, it would be reflected in earlier hospitalization dates. The consistency of our results when conditioning on (i) both the index case ascertainment and earliest hospitalization, versus (ii) only the earliest hospitalization, suggests there was a very limited period of cryptic spread before people began to be hospitalized for COVID-19 in late-2019.

Minimal cryptic circulation before December 2019. Although we do not see any evidence for substantial cryptic circulation before December 2019 with the epidemic simulations (Fig. 4), we can quantify the expected number of infections before the tMRCA. We calculate the cumulative number of infections in the epidemic simulations once they reach stable coalescence, the point in time at which basal lineages cease to be lost. Importantly, the time to stable coalescence is the equivalent to the tMRCA for the epidemic simulations. We observed, at most, 63 cumulative infections by the time of stable coalescence in the primary simulated epidemics, and 99% of simulated epidemics reached stable coalescence by 19 cumulative infections. With the tMRCA of lineage B, likely the first lineage of SARS-CoV-2 introduced into humans, estimated to 13 December (95% HPD: 29 November to 23 December), we would not expect more than a few dozen infections before 10 December, the date of the SARS-CoV-2 index case.

Similarities to WA1 and WA outbreak clades. To understand the phylogenetic signal of a hypothetical singular introduction of SARS-CoV-2 into humans, it is helpful to seek an analogy with the earliest introductions of SARS-CoV-2 into a new location, such as Washington State, Louisiana, or Lombardy, each of which had a polytomy (*11*, *28*, *29*). Here, we consider the earliest introductions of SARS-CoV-2 into North America. The first confirmed case of SARS-CoV-2 in the U.S. was associated with a virus strain ('WA1') isolated in Washington State from a traveler who returned from Wuhan, China, on 15 January 2020. There was subsequently an outbreak (henceforth, 'WA outbreak clade') in Washington State, with cases confirmed starting in February 2020 (*27*). As we have previously shown, although the MRCA of the WA outbreak clade differed from WA1 by only two substitutions, WA1 and the WA outbreak clade were, in fact, separate introductions into Washington State (*11*). The WA outbreak clade showcases a basal polytomy, and although the WA1 introduction was contained, onward transmission would have likely led to a basal polytomy as well, as shown by the hypothetical polytomy in Fig. S23. Therefore, this pattern is remarkably similar to that seen with lineages A and B, with the exception of a successful prevention of onward transmission from the WA1 case: the MRCA of WA1 and the WA outbreak clades was in China, and as we have shown here, the MRCA of lineages A and B was likely in the intermediate host reservoir (Fig. S30). Both scenarios show introductions of SARS-CoV-2 from a prior location: China in the case of WA1 and the WA outbreak clades, and the intermediate host reservoir in the case of lineages A and B. Similarly, both scenarios lead to (or would lead to, in the case of WA1) basal polytomies from the onward transmission. Therefore, lineages A and B look like

separate introductions because introductions with sustained onward transmission result in large basal polytomies.

MRCA of multiple introductions and the possibility of intermediate genomes. Although the unconstrained and recCA-rooted phylogenetic inferences most strongly support a lineage B and lineage A ancestral haplotype, respectively, the moderate support for a C/C ancestral haplotype from both inferences, the repeated observation of C8782T convergent evolution, and the C-to-T mutational bias suggest a C/C ancestral haplotype is also plausible. However, we acknowledge that if the MRCA were in an animal where we have no evidence of a C-to-T mutational bias, the T/T ancestral haplotype would also be possible.

If one of the "intermediate" genomes that shares additional mutations with lineage A or B (*i.e.*, at sites besides 8782 and 28144) is resequenced and validated as an intermediate C/C or T/T genome, shared synapomorphies should allow us to identify whether this intermediate haplotype is the result of convergent evolution. Phylogenetic placement can confirm whether the intermediate genome potentially represents the transition from lineage A to B (or B to A). Additionally, finding a true transitional intermediate genome would not resolve the conundrum with the molecular clock: (i) the unconstrained rooting model disfavors a lineage A root, whereas the recCA- and outgroup-rooted models favor a lineage A root, and (ii) lineage A exhibits less divergence from the root than would be expected if it were the sole ancestral virus in humans.

Lineages of SARS-CoV-2 introductions. The lineages introduced into humans are dependent on the viral diversity in the intermediate host, and the inferred ancestral haplotypes do not necessarily need to match the genomes of the introduced viruses. For example, lineages A and B are observed in humans, but the introduced viruses could have been of lineage A and C/C, with C/C quickly mutating into lineage B before leaving behind any descendant lineages. However, the simplest explanation would be lineage A and B progenitors circulating in animals and then these two lineages are separately introduced into humans, but other combinations of lineage introductions are plausible.

Importantly, these scenarios do not preclude an intermediate C/C or T/T haplotype from being introduced. If lineages A and B were present in the animal reservoir, a C/C or T/T haplotype could have circulated among the animals and then been introduced into humans as well. Considering the genomic data and high frequency of failed introductions in the epidemic simulations, the intermediate haplotype could have spread briefly among humans and then gone extinct. Therefore, it is not unreasonable to assume that an intermediate genome could appear in an environmental sample or an additional cross-species transmission.

Lastly, if a virus identical to or descendant from lineage B (or A) jumped into humans after the initial jump of B (or A), we would likely not have the phylogenetic resolution to detect this event as a separate introduction. Therefore, although the data are consistent with two introductions, it is possible that even more introductions into humans occurred at the Huanan market.

Table S1. Nucleotide variant calls at positions 8782 and 28144 for three SARS-CoV-2 genomes with intermediate T/T haplotypes[1].

GISAID accession	8782									28144								
	Depth	Count				Proportion				Depth	Count				Proportion			
		A	C	G	T	A	C	G	T		A	C	G	T	A	C	G	T
EPI_ISL_493179	64	0	39	1	24	0.000	0.609	0.016	0.375	61361	121	3784	195	57261	0.002	0.062	0.003	0.933
EPI_ISL_493180	40	0	24	1	15	0.000	0.600	0.025	0.375	95374	226	5709	293	89146	0.002	0.060	0.003	0.935
EPI_ISL_493182	29	0	10	0	19	0.000	0.345	0.000	0.655	69369	153	4051	232	64933	0.002	0.058	0.003	0.936

[1]Variant calls and depths provided by Di Liu and Yi Yan.

Table S2. Inferred tMRCAs for SARS-CoV-2, lineage B, and lineage A under different rooting strategies.

Phylodynamic analysis	SARS-CoV-2[1]	Lineage B[1]	Lineage A[1]
Unconstrained	12-11 (11-25 to 12-20)	12-13 (11-29 to 12-23)	12-25 (12-17 to 12-30)
recCA	12-06 (11-15 to 12-19)	12-15 (12-05 to 12-23)	12-20 (12-05 to 12-29)
Lineage B	12-12 (11-27 to 12-19)	12-13 (11-29 to 12-21)	12-25 (12-18 to 12-29)
C/C	12-08 (11-19 to 12-19)	12-16 (12-06 to 12-23)	12-21 (12-12 to 12-29)
T/T	12-08 (11-19 to 12-19)	12-15 (12-06 to 12-23)	12-21 (12-12 to 12-29)
Lineage A	12-07 (11-18 to 12-19)	12-16 (12-08 to 12-23)	12-18 (12-04 to 12-28)
Lineage A + C29095T	12-05 (11-17 to 12-19)	12-17 (12-10 to 12-23)	12-05 (11-17 to 12-19)
Lineage A.1	12-04 (11-16 to 12-18)	12-16 (12-10 to 12-23)	12-04 (11-16 to 12-19)
No markets	12-13 (11-25 to 12-26)	12-17 (11-29 to 12-26)	12-25 (12-15 to 01-04)

[1]Median and 95% HPD in parentheses.

Table S3. Simulation parameters, with all parameters except *b* and *h* based on Hao et al. (*64*). The *b* value listed is for the main analysis. The *h* parameter is not included in the Hao et al. model.

**Parameter**	**Definition**	**Value**
b	Transmission rate of ascertained cases	0.38
r	Ascertainment rate	0.15
a	Ratio of transmission for unascertained	0.55
h	Hospitalization rate	0.5
D_e	Latent period (days)	2.9
D_p	Presymptomatic infectious period (days)	2.3
D_i	Symptomatic infectious period (days)	2.9
D_q	Duration from illness onset to isolation (hospitalization) (days)	11
D_h	Isolation (hospitalization) period (days)	30

Table S4. The inferred doubling time for each transmission rate (*b*) and ascertainment rate (*r*) combination used in the simulations.

b	***r***	**Doubling time**[2]
0.22	0.15	4.45 (1.50-7.44)
0.28[1]	0.15	3.47 (1.35-5.44)
0.38	0.15	2.65 (1.50-4.10)
0.295	0.05	3.52 (1.38-5.64)
0.255	0.25	3.50 (1.51-5.65)

[1]Primary analysis.
[2]Median and 95% HDI in parentheses

Table S5. Frequencies of observed topologies in epidemic simulations and corresponding Bayes factor in favor of multiple introductions versus a single introduction across varying doubling times, varying ascertainment rate, minimum polytomy size, and phylogenetic rooting method.

Analysis			Topology			Bayes factor	
DT	Asc	Min. polytomy size	C/C	A/B	Polytomy	Unconstrained	recCA
2.65	0.15	100	0.0	1.2	58.6	28.8	29.5
3.47[1]	0.15	100	0.0	0.5	47.5	60.0	61.6
4.45	0.15	100	0.1	0.3	43.1	86.2	87.7
3.50	0.05	100	0.0	0.5	45.7	57.7	59.2
3.52	0.25	100	0.2	1.0	47.3	26.7	27.2
3.47	0.15	20	0.1	1.6	60.7	21.5	22.0
3.47	0.15	50	0.1	0.8	53.6	37.2	38.0
3.47	0.15	200	0.0	0.3	40.7	85.4	87.7
3.47	0.15	500	0.0	0.2	31.7	99.7	102.3

DT, Median doubling time
Asc, Ascertainment rate
Min., Minimum
[1]Primary analysis

Table S6. Number of days the timing of the primary case of lineage A occurs after the timing of the primary case of lineage B.

Phylodynamic analysis	Primary analysis[1,2]	Robustness analysis[1]			
	**DT: 3.47 Asc: 15%**	**DT: 2.65 Asc: 15%**	**DT: 4.45 Asc: 15%**	**DT: 3.50 Asc: 5%**	**DT: 3.52 Asc: 25%**
Unconstrained	6.7 (-28.9 to 44.4)	7.2 (-45.3 to 59.0)	7.0 (-20.5 to 35.1)	6.5 (-53.1 to 61.3)	7.0 (-36.4 to 48.1)
recCA	6.6 (-30.4 to 43.5)	6.6 (-44.9 to 58.9)	5.9 (-22.8 to 33.7)	6.2 (-48.7 to 63.4)	6.6 (-34.5 to 48.8)
Lineage B	6.6 (-28.4 to 44.4)	7.2 (-43.6 to 60.3)	7.2 (-20.3 to 34.9)	6.6 (-52.9 to 61.5)	6.9 (-35.9 to 48.9)
C/C	6.9 (-30.3 to 43.0)	7.2 (-45.5 to 59.4)	6.3 (-21.6 to 34.3)	6.5 (-47.5 to 65.8)	7.2 (-33.4 to 50.6)
Lineage A	6.9 (-30.2 to 43.5)	7.1 (-44.2 to 59.3)	5.7 (-22.7 to 33.8)	6.1 (-50.6 to 62.3)	6.9 (-35.7 to 49.2)

DT, Median doubling time
Asc, Ascertainment rate
[1]Median and 95% HPD in parentheses.
[2]See Fig. 3D for graphical representation of the full distribution.

Table S7. Time of the lineage B primary case under different robustness analyses for different lineage B index case and hospitalization dates and conditioning only on hospitalization dates.

Phylodynamic analysis	Primary analysis[1]	Robustness analysis[1]		
	Case: Dec 10 Hosp: Dec 16	Case: Dec 8 Hosp: Dec 16	Case: Dec 13 Hosp: Dec 18	Hosp: Dec 16
Unconstrained	11-18 (10-22 to 12-09)	11-18 (10-22 to 12-06)	11-21 (10-24 to 12-11)	11-20 (10-22 to 12-11)
recCA	11-18 (10-23 to 12-08)	11-17 (10-22 to 12-06)	11-21 (10-25 to 12-11)	11-20 (10-22 to 12-10)
Lineage B	11-19 (10-22 to 12-08)	11-17 (10-21 to 12-06)	11-21 (10-24 to 12-11)	11-20 (10-22 to 12-11)
C/C	11-18 (10-22 to 12-07)	11-17 (10-22 to 12-06)	11-21 (10-25 to 12-11)	11-20 (10-23 to 12-11)
Lineage A	11-18 (10-23 to 12-08)	11-17 (10-23 to 12-06)	11-21 (10-24 to 12-11)	11-20 (10-23 to 12-10)

Hosp, Hospitalization
[1]Median and 95% HPD in parentheses.

Table S8. Time of the lineage B primary case under different robustness analyses for different doubling times and ascertainment rates.

**Phylodynamic analysis**	**Primary analysis[1]**	**Robustness analysis[1]**			
	**DT: 3.47 Asc: 15%**	**DT: 2.65 Asc: 15%**	**DT: 4.45 Asc: 15%**	**DT: 3.5 Asc: 5%**	**DT: 3.52 Asc: 25%**
Unconstrained	11-18 (10-22 to 12-09)	11-25 (11-04 to 12-08)	11-14 (10-06 to 12-08)	11-17 (10-07 to 12-07)	11-21 (10-21 to 12-09)
recCA	11-18 (10-23 to 12-08)	11-26 (11-05 to 12-08)	11-14 (10-05 to 12-07)	11-17 (10-06 to 12-08)	11-21 (10-21 to 12-09)
Lineage B	11-19 (10-22 to 12-08)	11-25 (11-04 to 12-08)	11-14 (10-06 to 12-08)	11-17 (10-08 to 12-08)	11-21 (10-21 to 12-09)
C/C	11-18 (10-22 to 12-07)	11-26 (11-05 to 12-08)	11-14 (10-05 to 12-07)	11-17 (10-06 to 12-08)	11-21 (10-21 to 12-09)
Lineage A	11-18 (10-23 to 12-08)	11-26 (11-05 to 12-08)	11-14 (10-06 to 12-07)	11-17 (10-06 to 12-09)	11-21 (10-21 to 12-09)

DT, Median doubling time

Asc, Ascertainment rate

[1]Median and 95% HPD in parentheses.

Table S9. Time of the lineage A primary case results when conditioning with either both the index and hospitalization dates or just the hospitalization date.

**Phylodynamic analysis**	**Primary analysis[1]**	**Robustness analysis[1]**
	**Case: Dec 15<br>Hosp: Dec 25**	**Hosp: Dec 25**
Unconstrained	11-25<br>(10-31 to 12-13)	11-28<br>(10-31 to 12-19)
recCA	11-25<br>(10-29 to 12-14)	11-29<br>(10-30 to 12-19)
Lineage B	11-25<br>(11-01 to 12-13)	11-28<br>(11-01 to 12-19)
C/C	11-25<br>(10-30 to 12-13)	11-29<br>(10-31 to 12-20)
Lineage A	11-25<br>(10-29 to 12-14)	11-29<br>(10-30 to 12-19)

Hosp, Hospitalization
[1]Median and 95% HPD in parentheses.

Table S10. Time of the lineage A primary case under different doubling times and ascertainment rates.

Phylodynamic analysis	Primary analysis[1]	Robustness analysis[1]			
	**DT: 3.47 Asc: 15%**	**DT: 2.65 Asc: 15%**	**DT: 4.45 Asc: 15%**	**DT: 3.5 Asc: 5%**	**DT: 3.52 Asc: 25%**
Unconstrained	11-25 (10-31 to 12-13)	12-02 (11-12 to 12-13)	11-21 (10-13 to 12-13)	11-24 (10-12 to 12-14)	11-28 (10-29 to 12-14)
recCA	11-25 (10-29 to 12-14)	12-02 (11-10 to 12-14)	11-21 (10-12 to 12-13)	11-23 (10-13 to 12-13)	11-28 (10-29 to 12-14)
Lineage B	11-25 (11-01 to 12-13)	12-02 (11-13 to 12-14)	11-21 (10-14 to 12-13)	11-24 (10-11 to 12-14)	11-28 (10-29 to 12-14)
C/C	11-25 (10-30 to 12-13)	12-02 (11-11 to 12-14)	11-21 (10-13 to 12-13)	11-24 (10-13 to 12-12)	11-28 (10-29 to 12-14)
Lineage A	11-25 (10-29 to 12-14)	12-01 (11-09 to 12-14)	11-21 (10-13 to 12-13)	11-23 (10-14 to 12-12)	11-28 (10-29 to 12-14)

DT, Median doubling time

Asc, Ascertainment rate

[1]Median and 95% HPD in parentheses.

Table S11. Number of estimated infections on 1 December 2019 for lineage A, lineage B, lineages A and B combined, and single introduction simulations.

Phylodynamic analysis	Introductions[1]			
	Lineage A	Lineage B	Lineages A and B combined	Single introduction
**Unconstrained**	2 (0, 23)	4 (0, 27)	8 (0, 38)	5 (0, 31)
**recCA**	2 (0, 24)	4 (0, 25)	8 (0, 36)	6 (0, 85)
**Lineage B**	2 (0, 23)	4 (0, 27)	8 (0, 38)	5 (0, 29)
**C/C**	2 (0, 23)	4 (0, 25)	8 (0, 35)	5 (0, 51)
**Lineage A**	2 (0, 23)	4 (0, 25)	8 (0, 35)	6 (0, 58)

[1]Median and 95% HPD in parentheses.

Table S12. Number of estimated infections on 15 December 2019 for lineage A, lineage B, lineages A and B combined, and single introduction simulations.

Phylodynamic analysis	Introductions[1]			
	Lineage A	Lineage B	Lineages A and B combined	Single introduction
**Unconstrained**	8 (1, 33)	16 (2, 251)	28 (3, 273)	17 (2, 517)
**recCA**	9 (1, 57)	14 (2, 63)	26 (3, 153)	31 (2, 3094)
**Lineage B**	8 (1, 33)	16 (2, 246)	28 (3, 259)	16 (2, 303)
**C/C**	8 (1, 35)	14 (2, 62)	25 (3, 81)	24 (2, 1640)
**Lineage A**	9 (1, 59)	13 (2, 52)	26 (3, 117)	26 (2, 2095)

[1]Median and 95% HPD in parentheses.

Table S13. Number of infections at the tMRCA and hospitalizations on 1 December 2019.

	Time	Primary analysis[1]	Robustness analysis[1]			
		**DT: 3.47 Asc: 15%**	**DT: 2.65 Asc: 15%**	**DT: 4.45 Asc: 15%**	**DT: 3.50 Asc: 5%**	**DT: 3.52 Asc: 25%**
**Infections**	**tMRCA**	3 (1-18)	3 (1-12)	5 (1-39)	5 (1-29)	3 (1-18)
**Hospitalizations**	**1 Dec 2019**	0 (0–2)	0 (0–1)	1 (0–3)	0 (0–2)	0 (0–3)

DT, Median doubling time

Asc, Ascertainment rate

[1]Median and 95% HPD in parentheses.

Table S14. Time of the primary case of a hypothetical single-introduction scenario under different robustness analyses for different index case dates and conditioning on only hospitalization dates.

**Phylodynamic analysis**	**Primary analysis**[1]	**Robustness analysis**[1]	
	**Case: Dec 10 Hosp: Dec 16**	**Case: Dec 8 Hosp: Dec 16**	**Hosp: Dec 16**
Unconstrained	11-18 (10-22 to 12-08)	11-18 (10-21 to 12-06)	11-20 (10-21 to 12-11)
recCA	11-18 (10-20 to 12-08)	11-17 (10-19 to 12-06)	11-19 (10-19 to 12-11)
Hu-1	11-19 (10-23 to 12-09)	11-18 (10-22 to 12-06)	11-20 (10-22 to 12-11)
C/C	11-18 (10-20 to 12-08)	11-17 (10-20 to 12-06)	11-20 (10-20 to 12-11)
T/T	11-18 (10-20 to 12-08)	11-17 (10-20 to 12-07)	11-20 (10-20 to 12-11)
Lineage A	11-18 (10-20 to 12-08)	11-17 (10-19 to 12-06)	11-19 (10-20 to 12-11)
Lineage A + C29095T	11-18 (10-19 to 12-08)	11-17 (10-19 to 12-07)	11-19 (10-19 to 12-10)
Lineage A.1	11-18 (10-19 to 12-08)	11-17 (10-19 to 12-07)	11-19 (10-18 to 12-09)

Hosp, Hospitalization
[1]Median and 95% HPD in parentheses.

Table S15. Time of the primary case of a hypothetical single-introduction scenario under different doubling times and ascertainment rates.

**Phylodynamic analysis**	**Primary analysis[1]**	**Robustness analysis[1]**			
	**DT: 3.47 Asc: 15%**	**DT: 2.65 Asc: 15%**	**DT: 4.45 Asc: 15%**	**DT: 3.5 Asc: 5%**	**DT: 3.52 Asc: 25%**
Unconstrained	11-18 (10-22 to 12-08)	11-25 (11-03 to 12-08)	11-14 (10-05 to 12-08)	11-17 (10-08 to 12-07)	11-21 (10-21 to 12-09)
recCA	11-18 (10-20 to 12-08)	11-23 (10-30 to 12-08)	11-13 (10-03 to 12-06)	11-15 (10-09 to 12-07)	11-19 (10-20 to 12-08)
Lineage B	11-19 (10-23 to 12-09)	11-25 (11-03 to 12-08)	11-14 (10-05 to 12-08)	11-17 (10-08 to 12-08)	11-21 (10-21 to 12-09)
C/C	11-18 (10-20 to 12-08)	11-24 (11-01 to 12-08)	11-13 (10-04 to 12-07)	11-16 (10-09 to 12-07)	11-20 (10-20 to 12-09)
T/T	11-18 (10-20 to 12-08)	11-24 (11-01 to 12-08)	11-13 (10-03 to 12-07)	11-16 (10-10 to 12-07)	11-20 (10-20 to 12-09)
Lineage A	11-18 (10-20 to 12-08)	11-24 (10-31 to 12-08)	11-13 (10-04 to 12-07)	11-16 (10-11 to 12-07)	11-20 (10-20 to 12-08)
Lineage A + C29095T	11-18 (10-19 to 12-08)	11-23 (10-31 to 12-08)	11-13 (10-03 to 12-06)	11-16 (10-10 to 12-07)	11-19 (10-20 to 12-08)
Lineage A.1	11-18 (10-19 to 12-08)	11-23 (10-30 to 12-08)	11-13 (10-04 to 12-07)	11-16 (10-10 to 12-07)	11-19 (10-20 to 12-08)

DT, Median doubling time

Asc, Ascertainment rate

[1]Median and 95% HPD in parentheses.

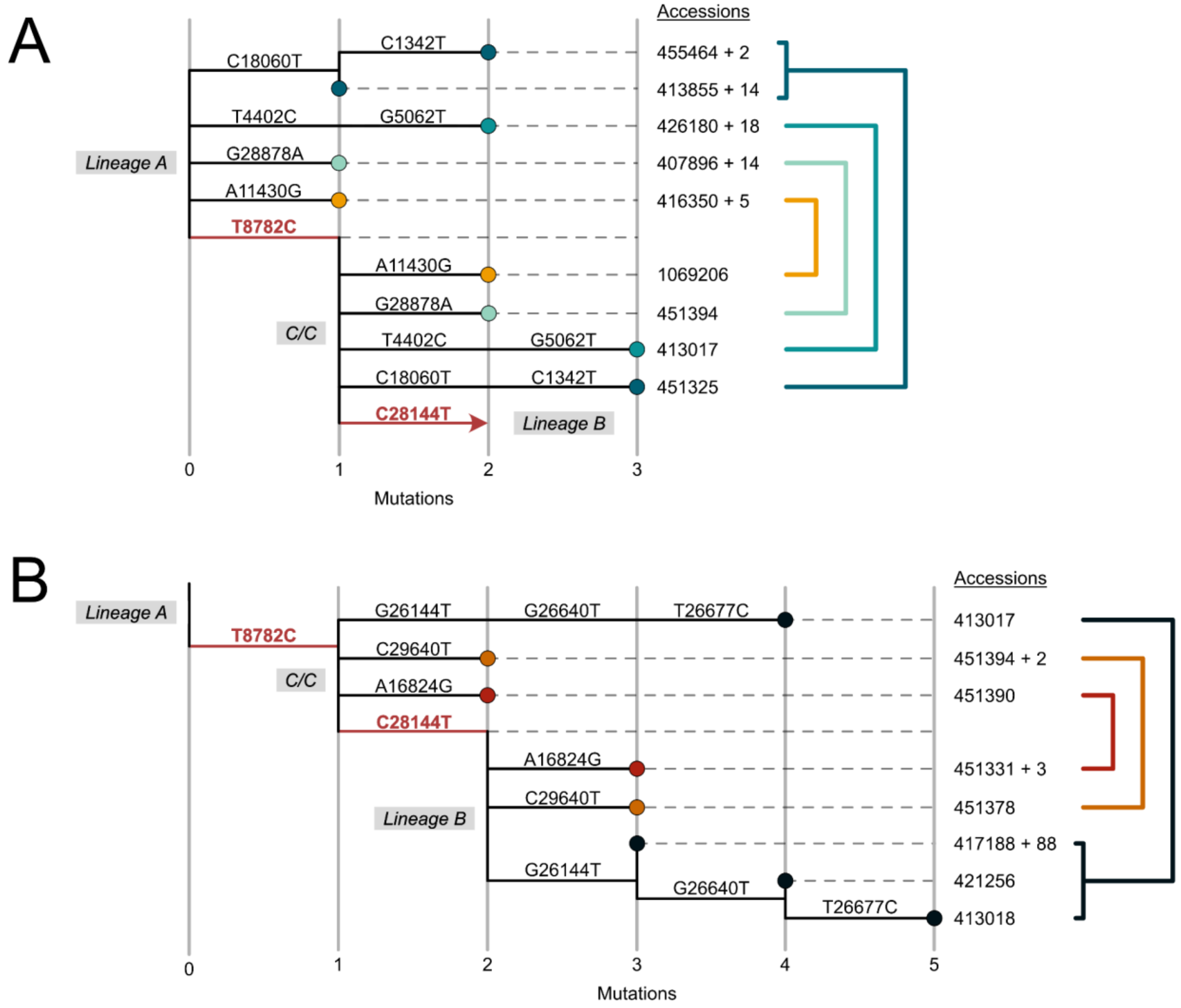

Figure S1. Mutation map of SARS-CoV-2 intermediate C/C genomes and their shared mutations within lineages A and B. (**A**) Shared mutations across lineage A and C/C. (**B**) Shared mutations across lineage B and C/C. Mutations relative to the Hu-1 reference genome are shown above each branch. Lineage-defining mutations (8782 and 28144) are colored in red. Derived mutations not shared by both lineages are excluded. The taxon names are GISAID accession numbers, and the total number of additional matching homoplastic sequences are indicated. Sequences that share derived mutations are connected by the lines on the right, and brackets indicate that a group of sequences share the derived mutations that cannot be individually resolved.

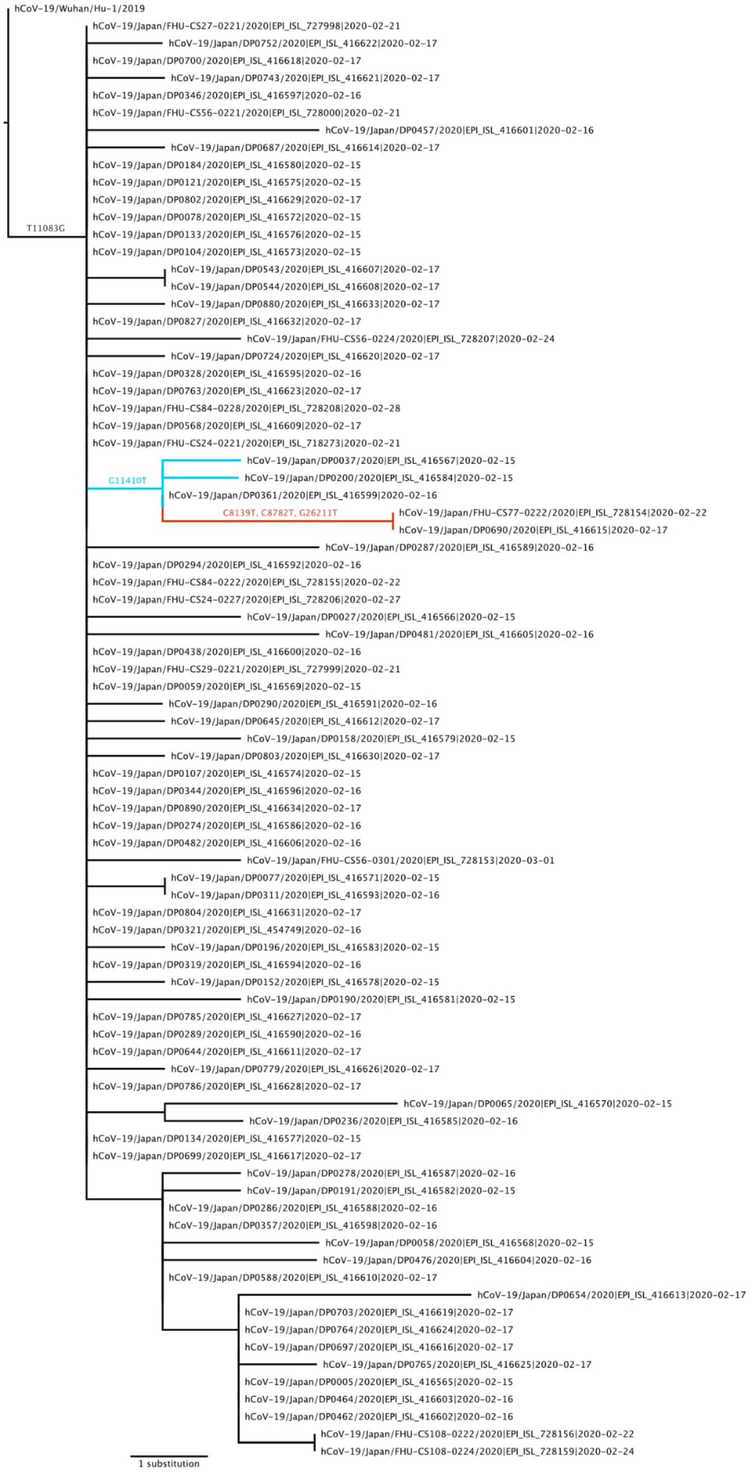

Figure S2. Maximum likelihood phylogeny of SARS-CoV-2 genomes from the Diamond Princess outbreak. The tree is rooted on Hu-1. Substitutions found in T/T genomes relative to Hu-1 annotated on branches. The G11410T clade is colored blue, with the branch leading to the T/T genomes colored red.

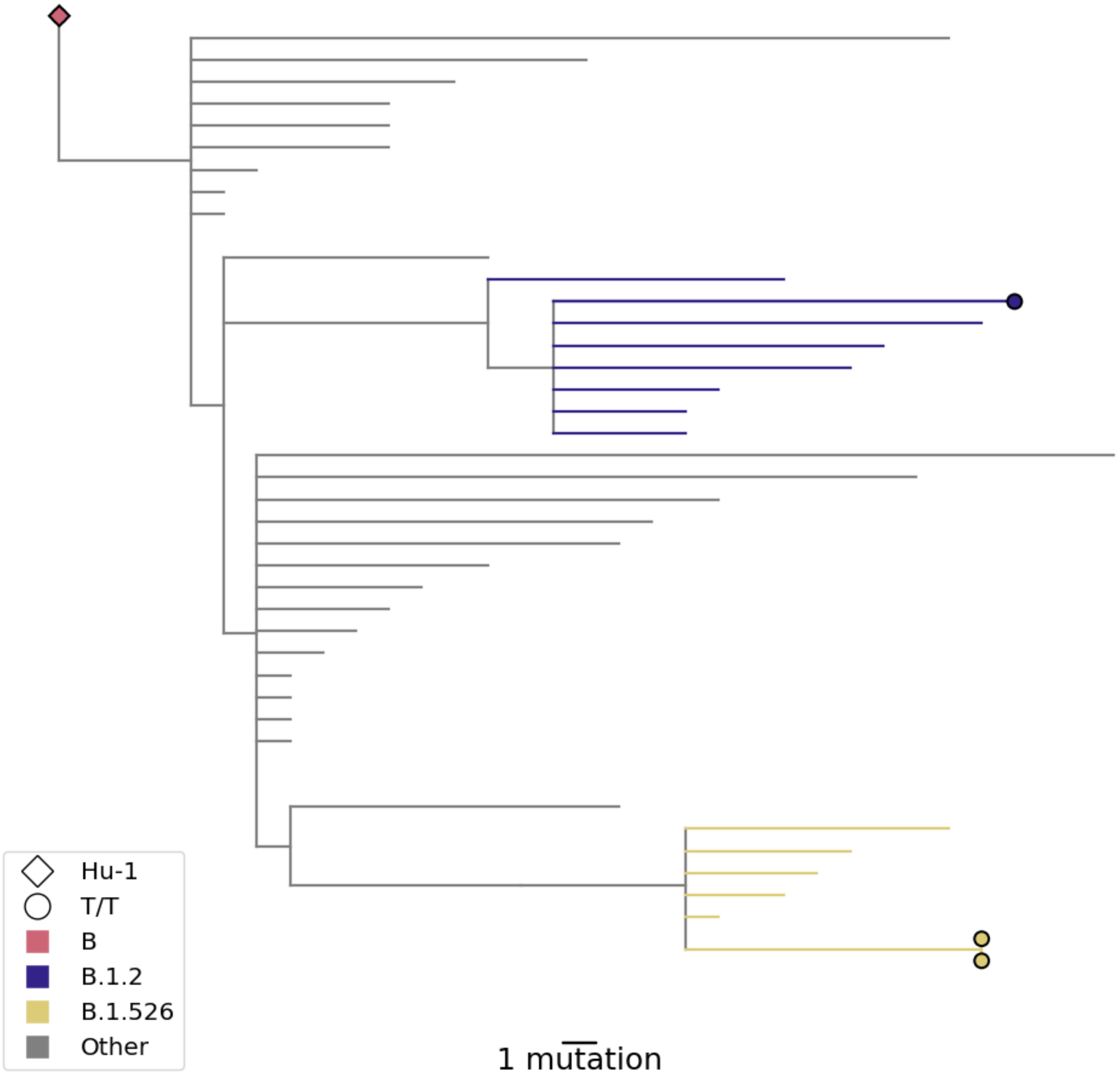

Figure S3. Subtree showing the placement of T/T genomes in the NYC Public Health Laboratories dataset. 3 T/T genomes were placed on a global tree of 3 million genomes (v2022-01-21) using UShER. The node branches are colored by the assigned PANGO lineage. The T/T genomes are highlighted using circles.

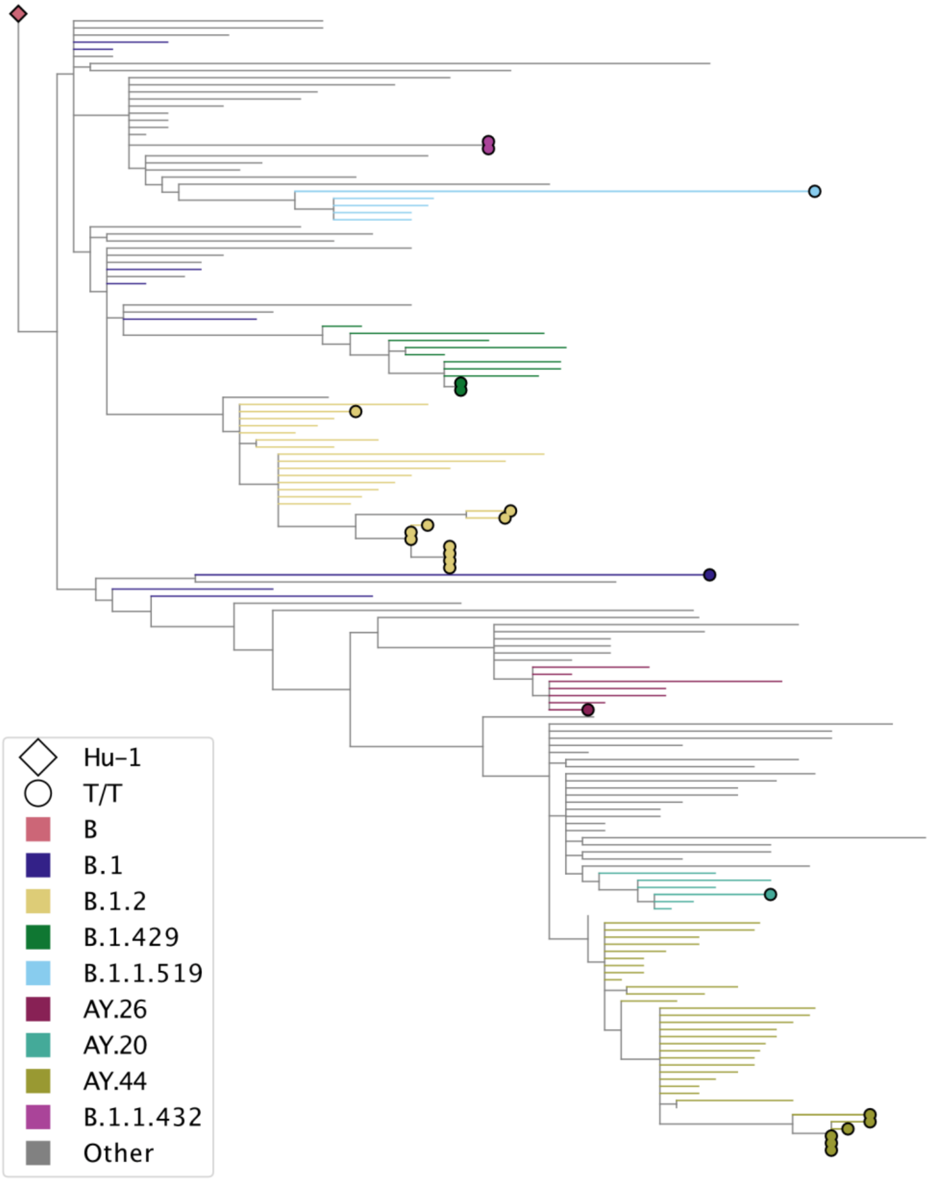

Figure S4. Subtree showing the placement of T/T genomes in the San Diego SEARCH dataset. 24 T/T genomes were placed on a global tree of 3 million genomes (v2022-01-21) using UShER. The node branches are colored by the assigned PANGO lineage. The T/T genomes are highlighted using circles.

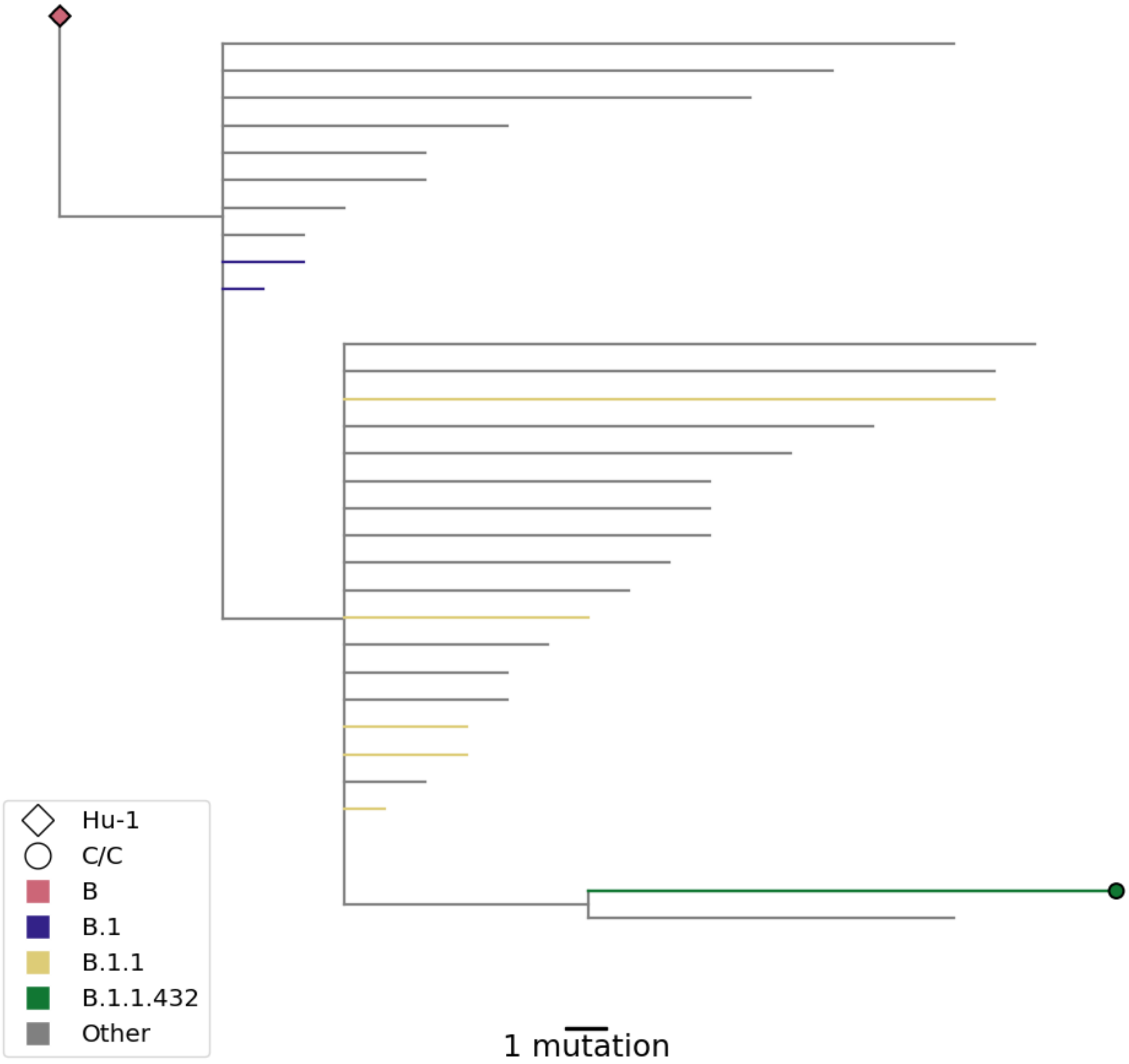

Figure S5. Subtree showing the placement of C/C genome in the San Diego SEARCH dataset. 1 C/C genome was placed on a global tree of 3 million genomes (v2022-01-21) using UShER. The node branches are colored by the assigned PANGO lineage. The C/C genome is highlighted using a circle.

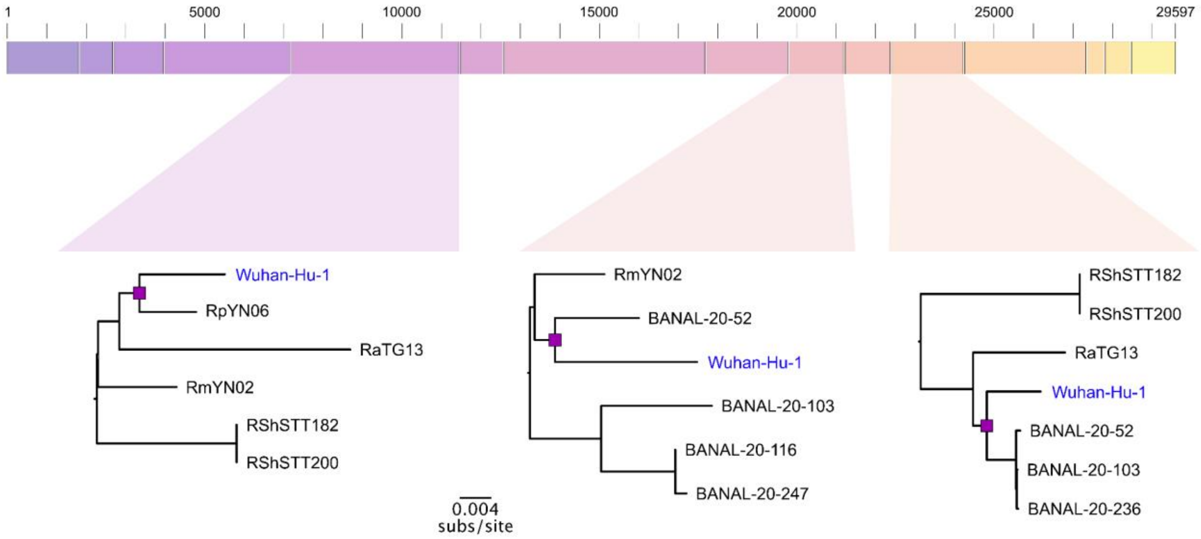

Figure S6. Reconstructing the recombinant common ancestor (recCA) of SARS-CoV-2 infecting a non-human animal. The figure identifies 15 non-recombinant regions of SARS-CoV-2-like sarbecovirus genomes. Subtrees from sarbecovirus maximum likelihood phylogenies of example regions 5, 9, and 11 show the genomes most closely related to SARS-CoV-2. Ancestral state reconstruction at the MRCA (purple square) of SARS-CoV-2 (Wuhan-Hu-1) and the most closely related sarbecovirus for each of the 15 fragments is concatenated to construct the recCA.

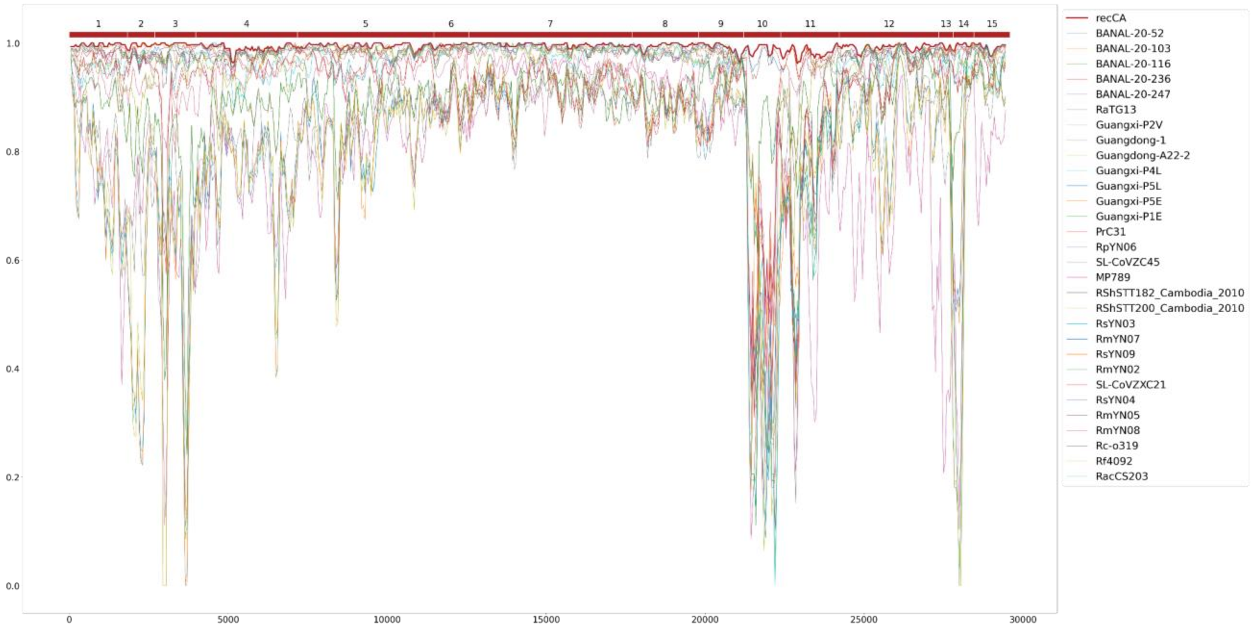

Figure S7. Simplot of closely related sarbecoviruses and recCA with Hu-1 as the reference.

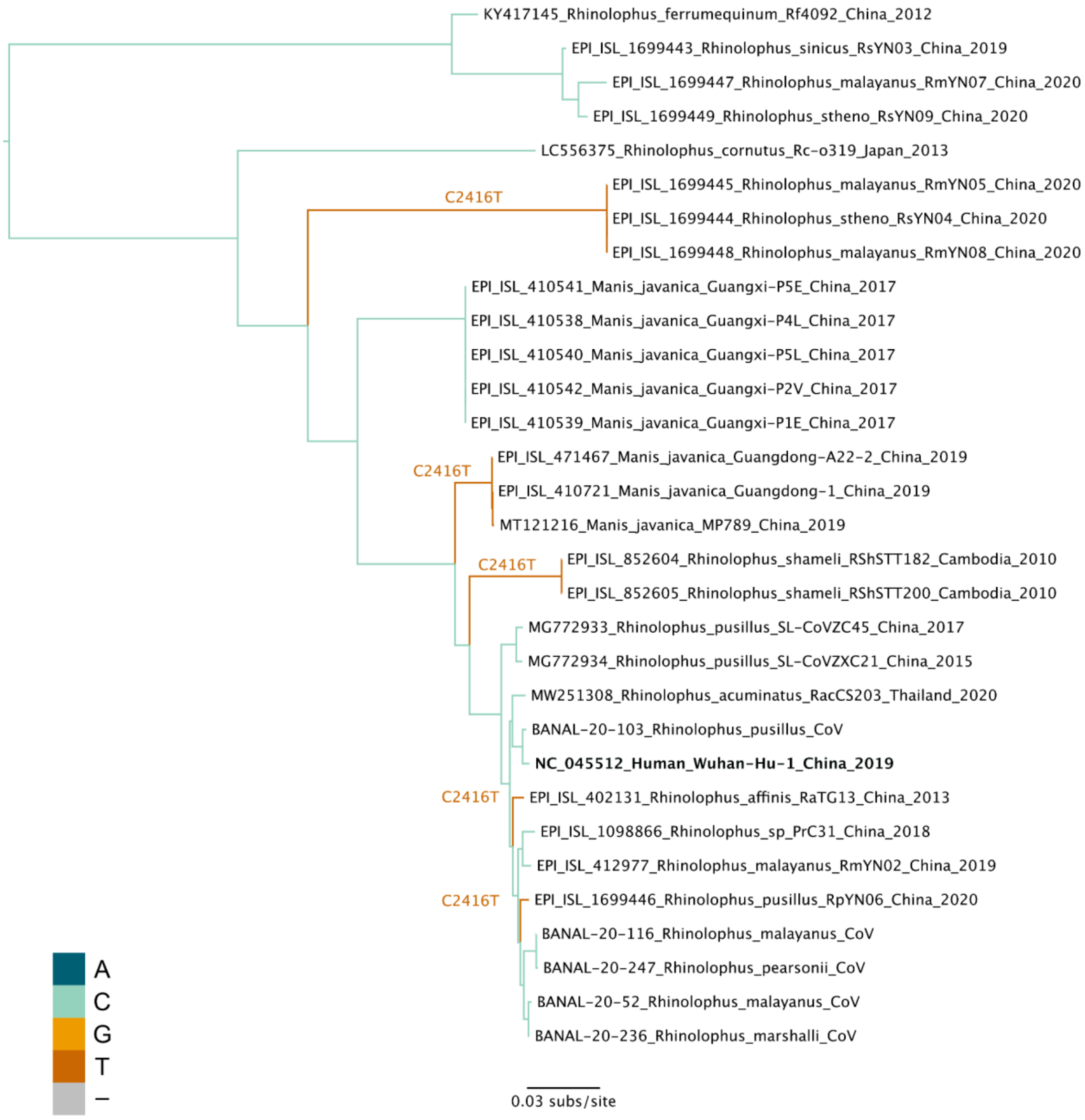

Figure S8. Maximum likelihood tree of non-recombinant region 2 with branches colored based on the nucleotide at position 2416. Some substitution labels shifted for clarity.

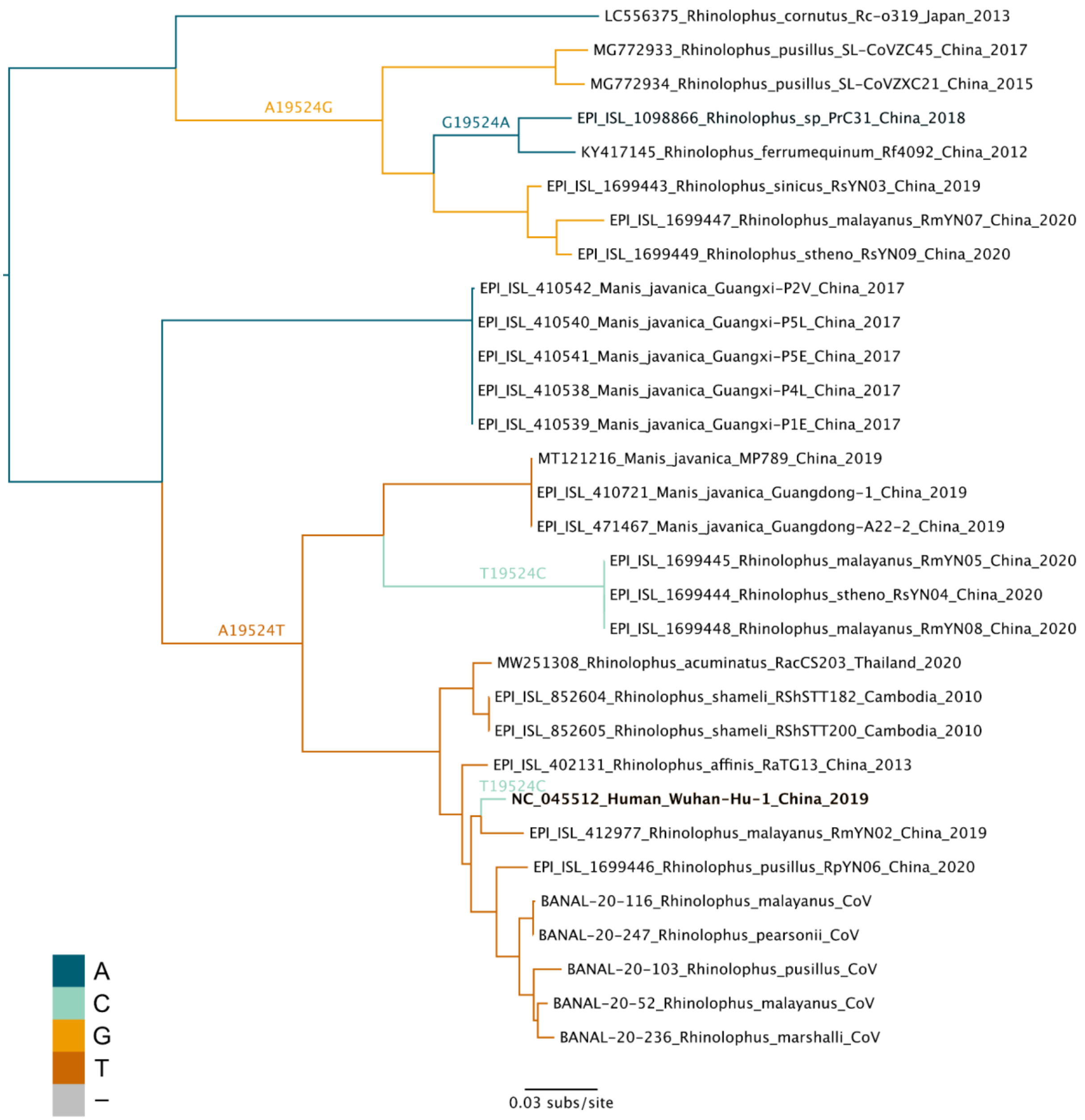

Figure S9. Maximum likelihood tree of non-recombinant region 8 with branches colored based on the nucleotide at position 19524. Some substitution labels shifted for clarity.

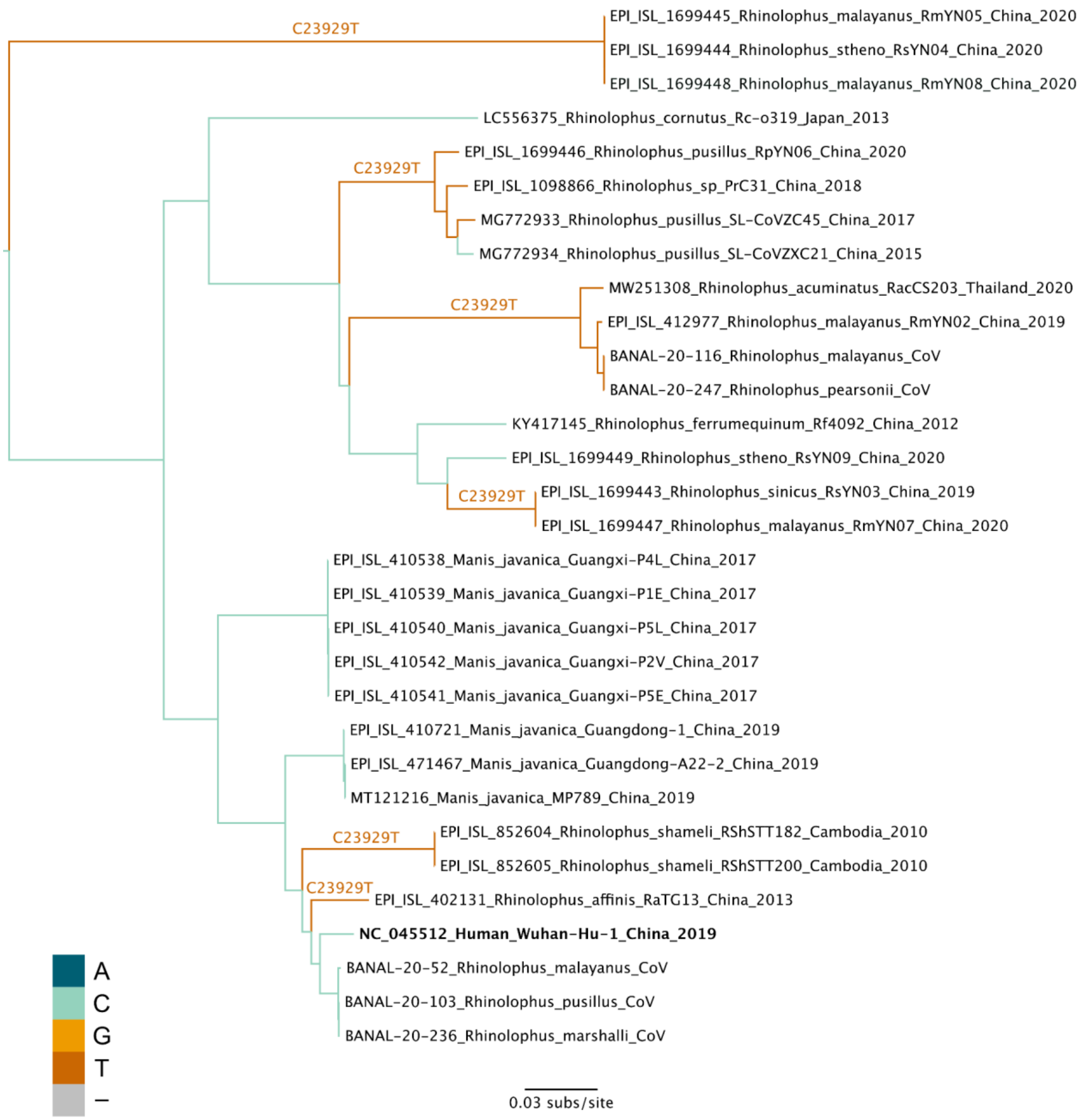

Figure S10. Maximum likelihood tree of non-recombinant region 11 with branches colored based on the nucleotide at position 23929. Some substitution labels shifted for clarity.

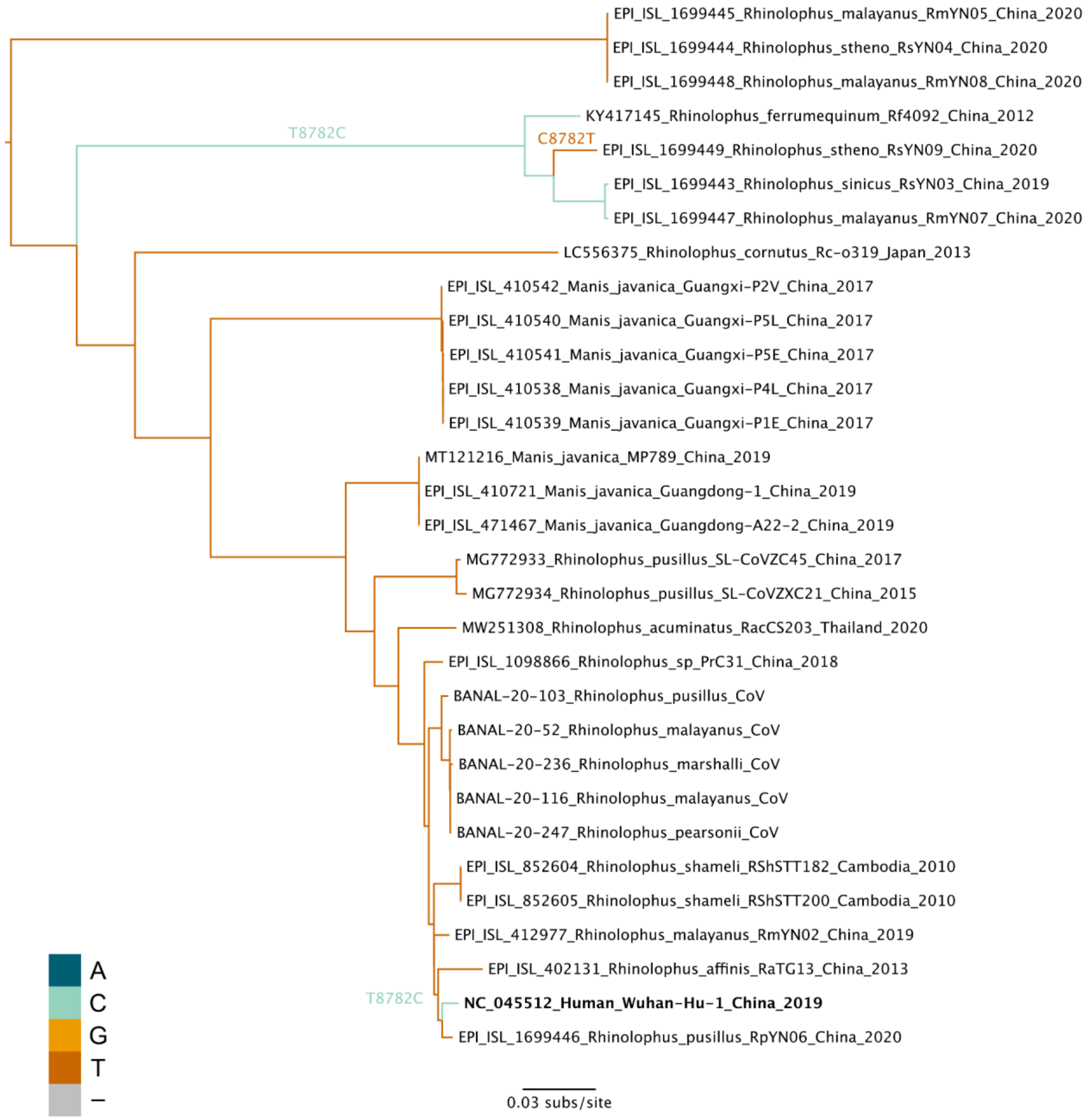

Figure S11. Maximum likelihood tree of non-recombinant region 5 with branches colored based on nucleotide at position 8782. Some substitution labels shifted for clarity.

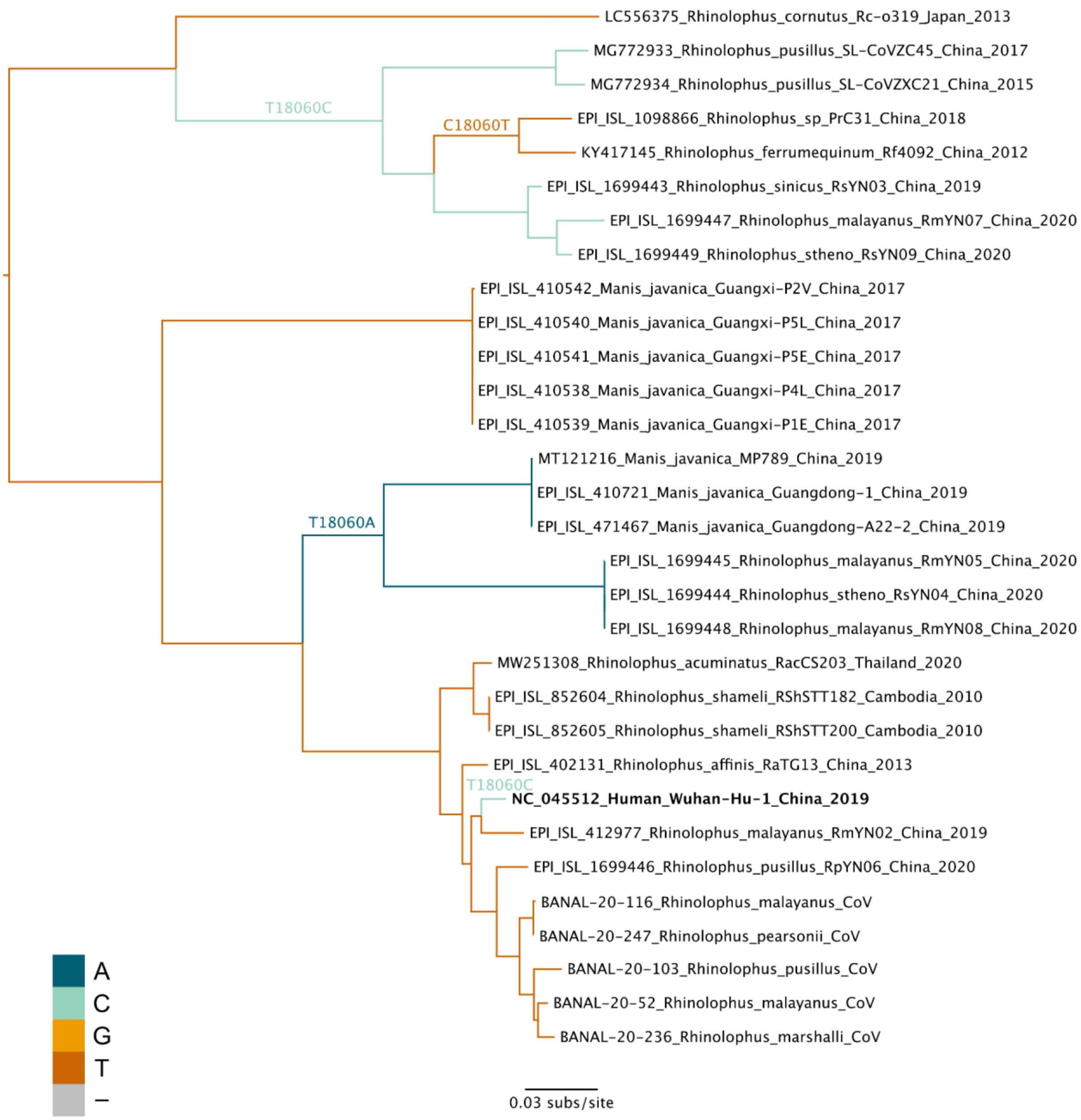

Figure S12. Maximum likelihood tree of non-recombinant region 8 with branches colored based on the nucleotide at position 18060.

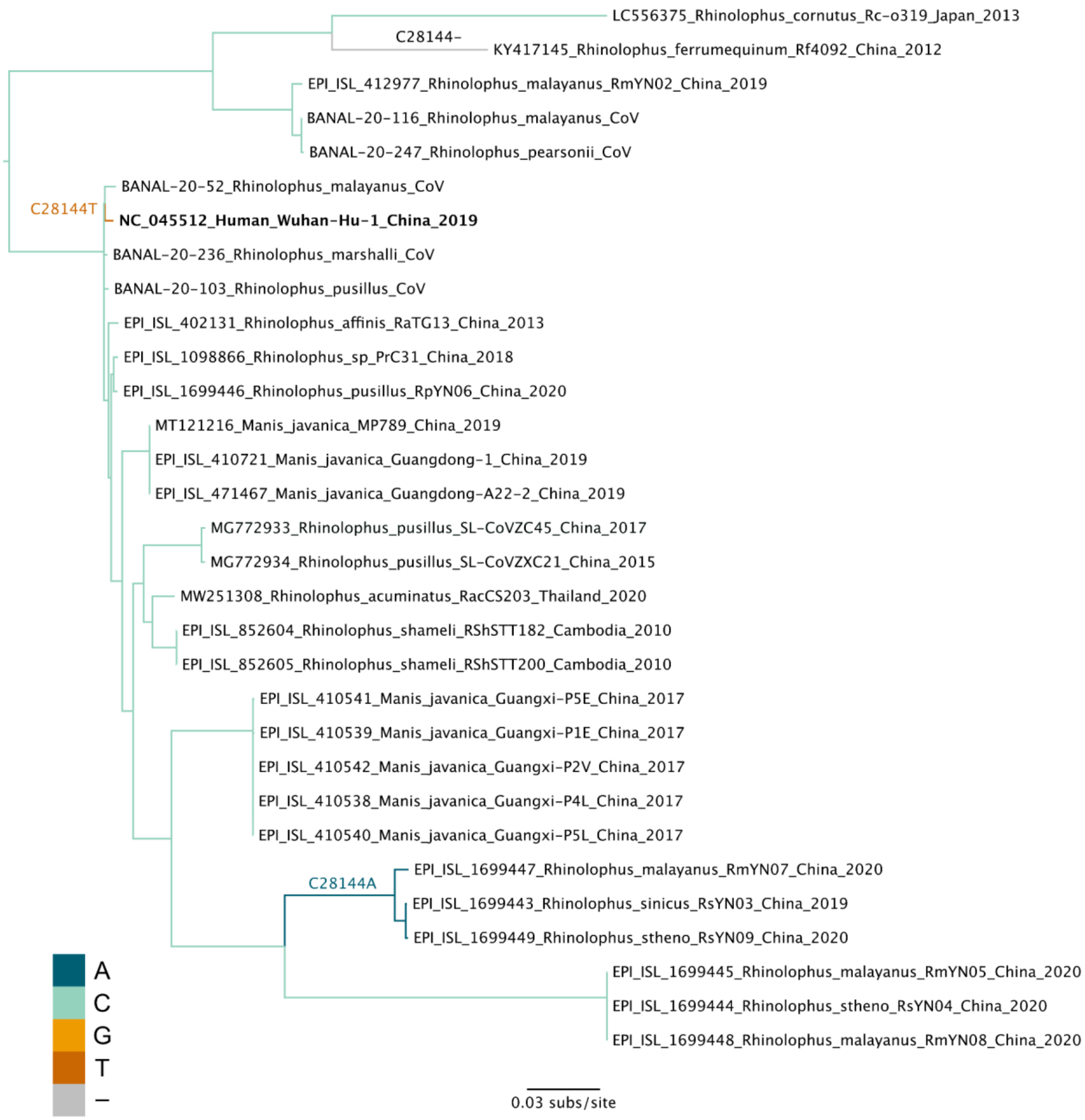

Figure S13. Maximum likelihood tree of non-recombinant region 14 with branches colored based on the nucleotide at position 28144. Some substitution labels shifted for clarity.

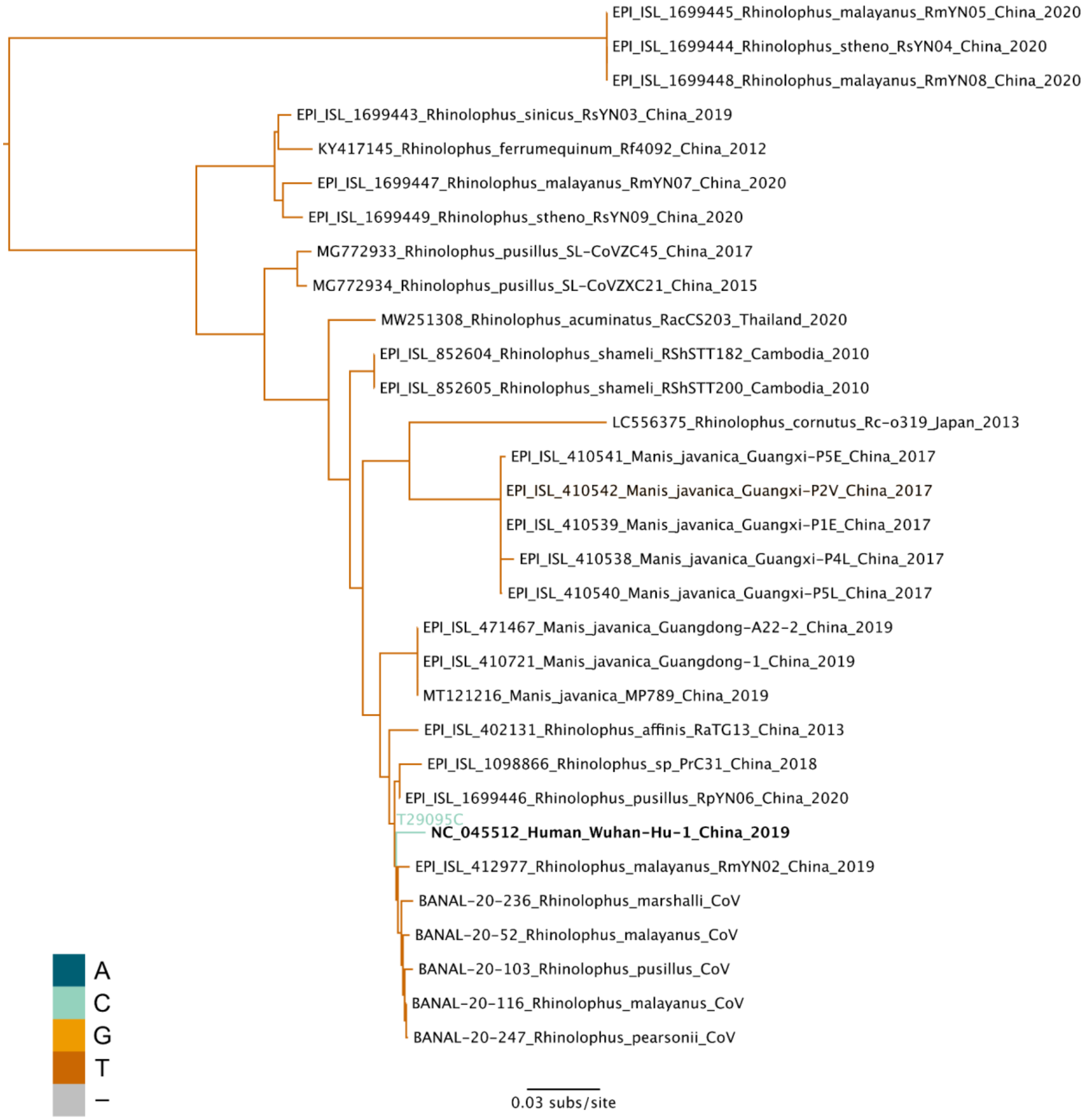

Figure S14. Maximum likelihood tree of non-recombinant region 15 with branches colored based on the nucleotide at position 29095.

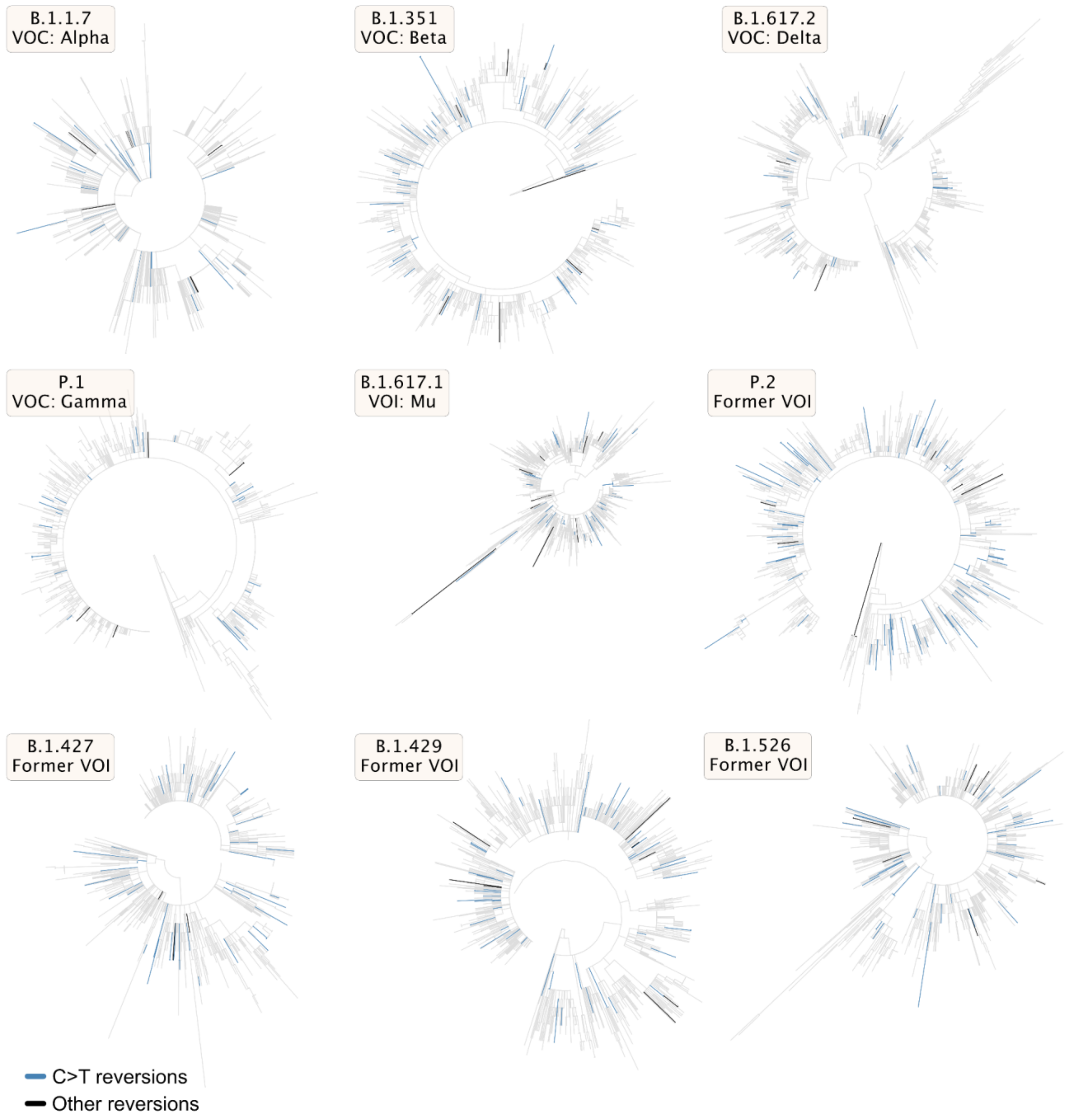

Figure S15. Maximum likelihood phylogenies of variants of concern (VOC) and variants of interest (VOI) with branches containing reversions colored.

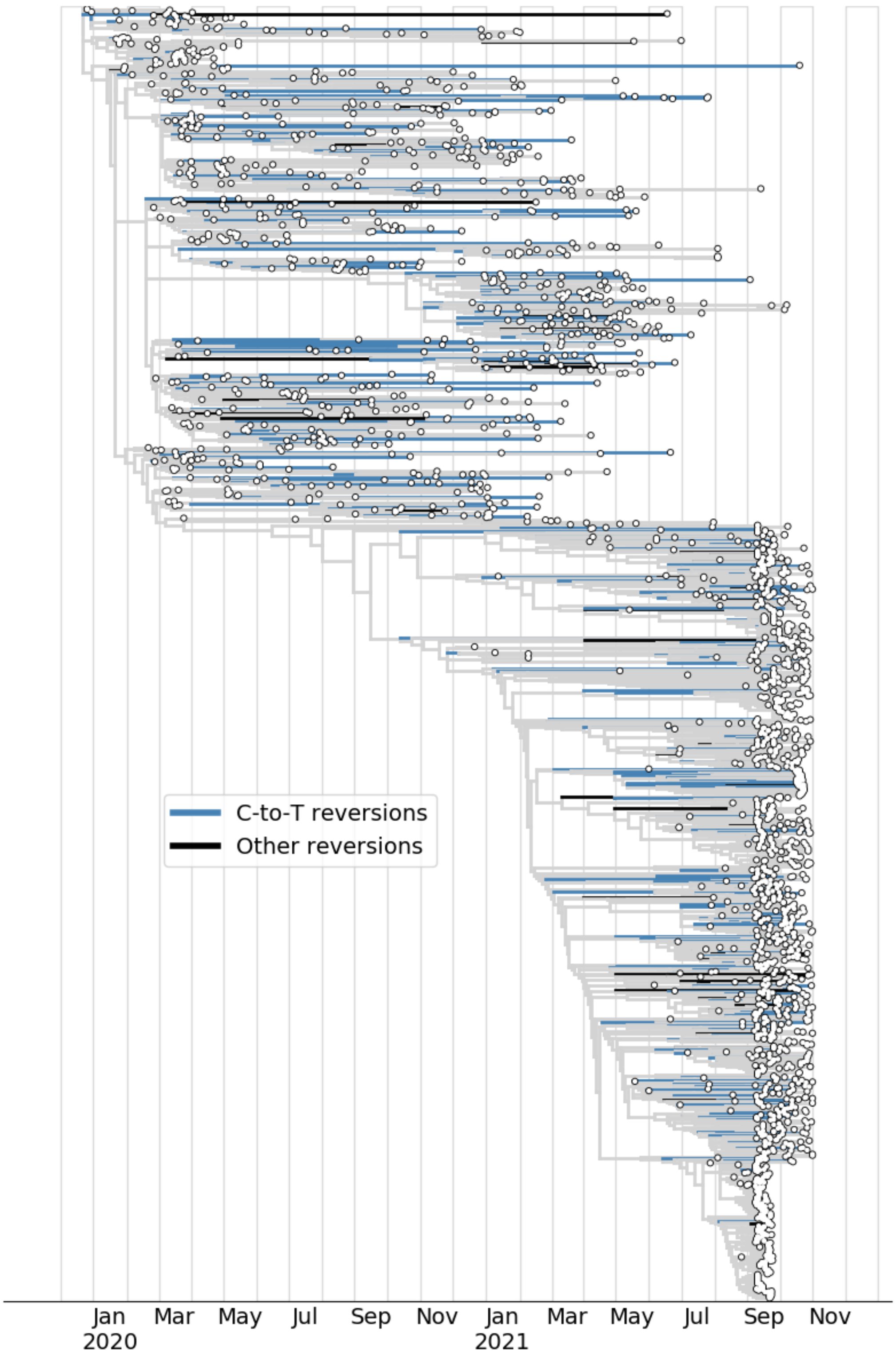

Figure S16. Subsampled global phylogeny showing reversions. Subsampled SARS-CoV-2 time-resolved phylogeny from Nextstrain, with reversions colored blue if a C-to-T reversion and black otherwise.

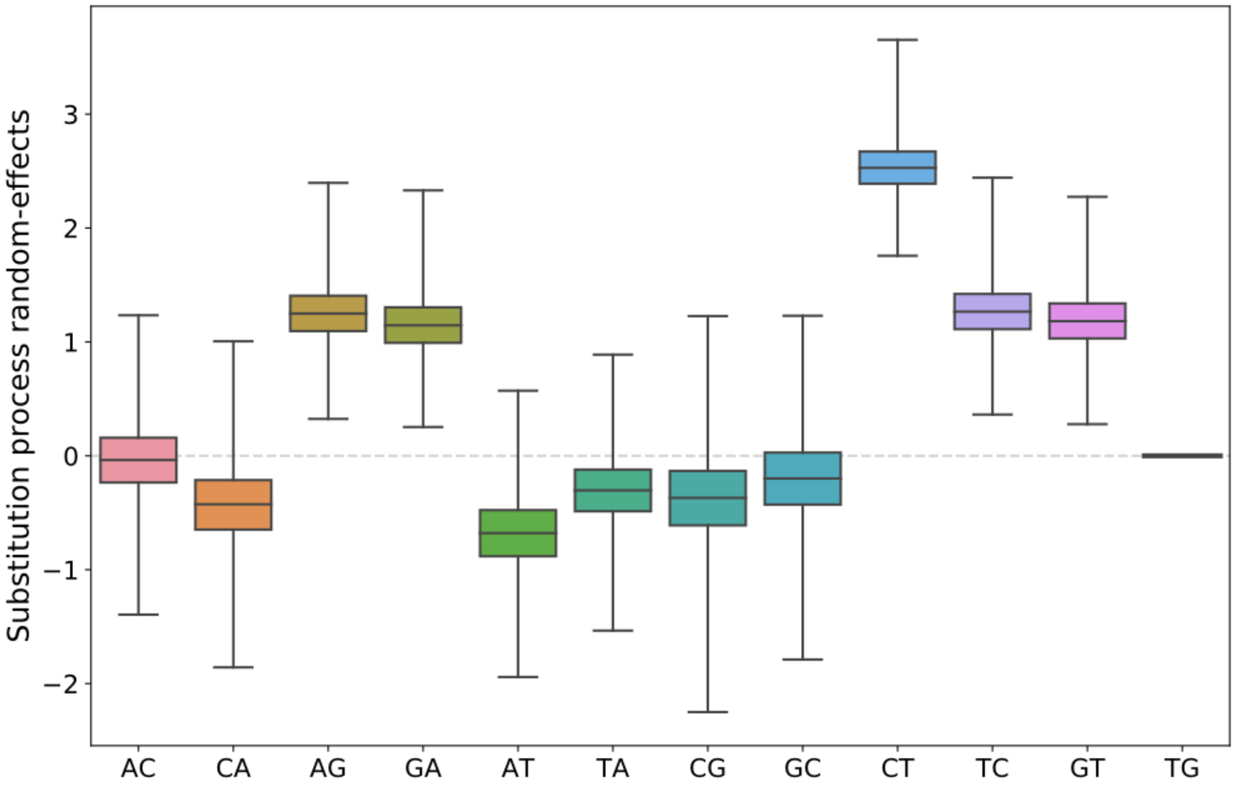

Figure S17. Substitution process random-effects for the unconstrained rooting model. The random effects for transitions were rescaled with κ, and then all random effects were made relative to T-to-G (fixed to 0). The posterior probabilities that $e^{C\text{-}to\text{-}T} > e^{T\text{-}to\text{-}C}$ and $e^{G\text{-}to\text{-}T} > e^{T\text{-}to\text{-}G}$ is 1.00 for both, indicating the C-to-T transition and G-to-T transversion biases were present in every sample in the posterior.

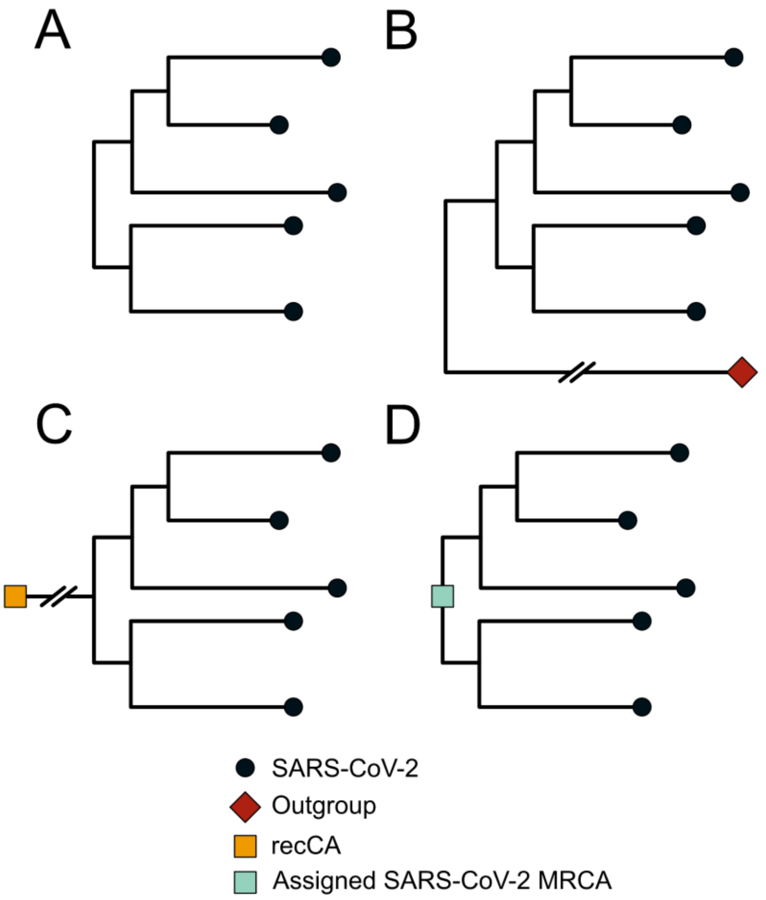

Figure S18. Schematic depicting the rooting strategies used in different phylodynamic models. (**A**) An unconstrained rooting model with only SARS-CoV-2 where the root is inferred from the molecular clock calibrated using SARS-CoV-2 sampling dates. (**B**) An unconstrained rooting with SARS-CoV-2 and a sarbecovirus outgroup. (**C**) A constrained model where the ancestor of SARS-CoV-2 is constrained to be the recombinant common ancestor (recCA). (**D**) A constrained model with only SARS-CoV-2, but the MRCA forced to be a pre-specified haplotype.

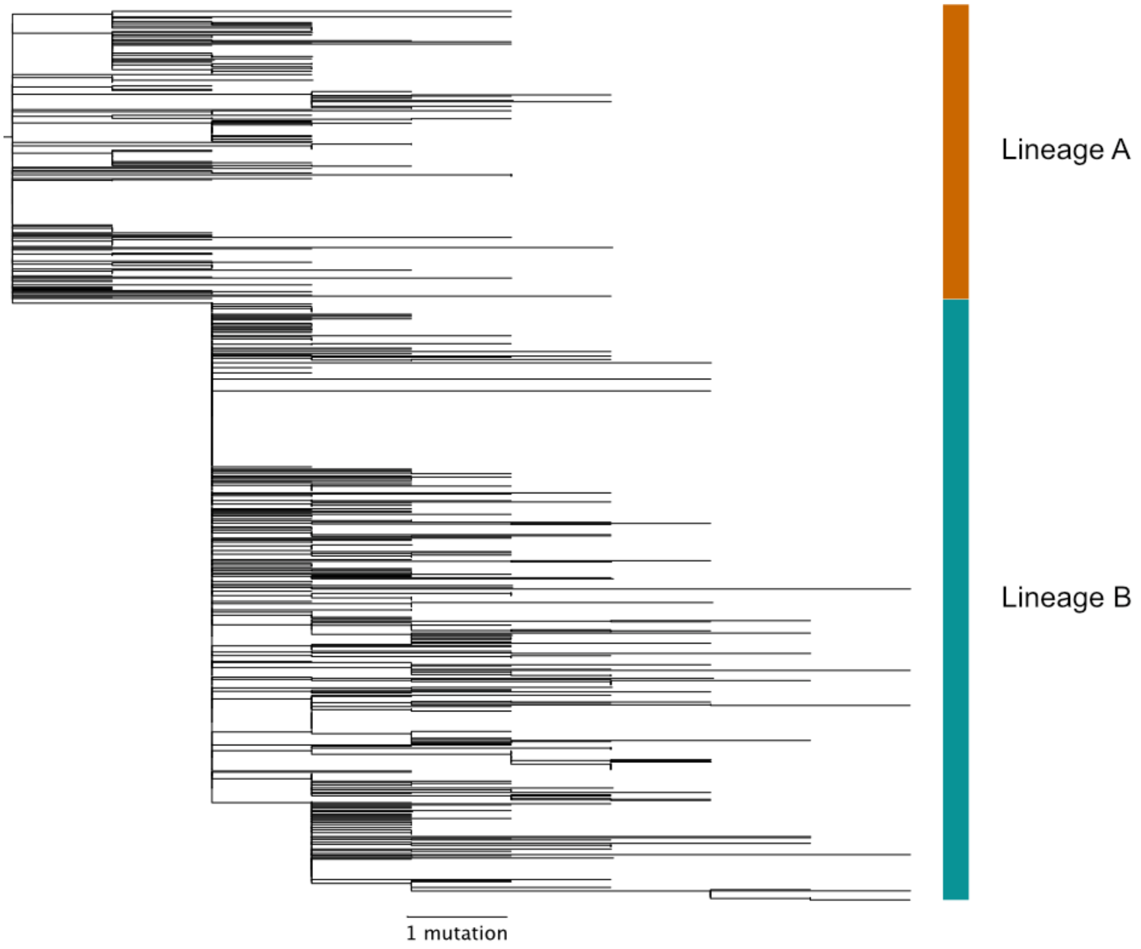

Figure S19. SARS-CoV-2 maximum likelihood tree rooted on lineage A (n=787 taxa, through 14 February 2020).

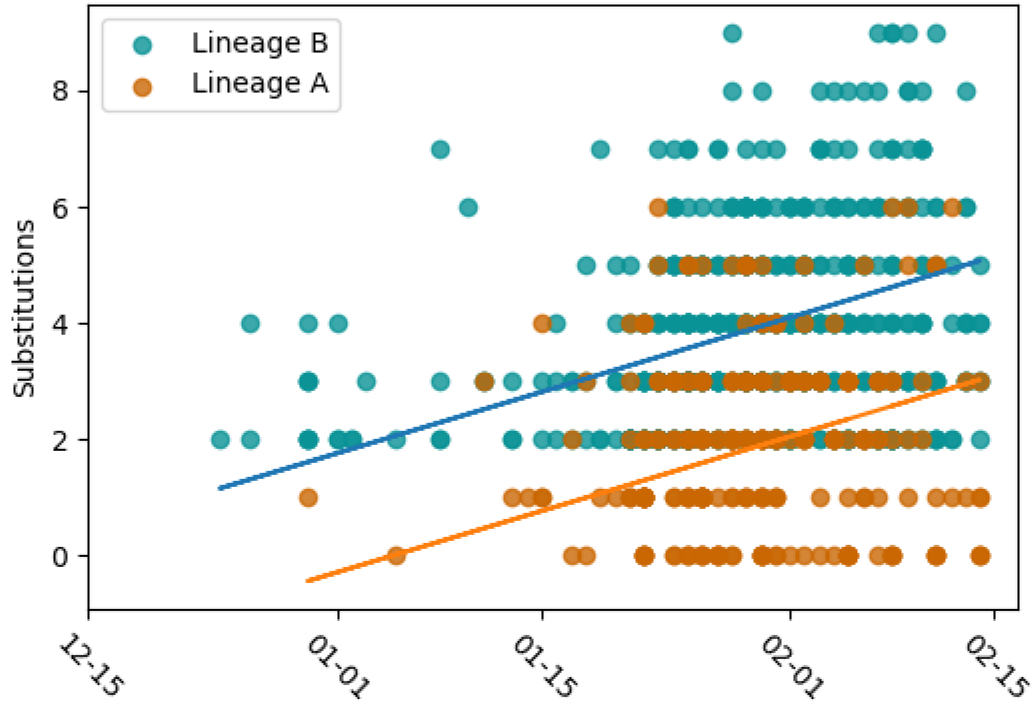

Figure S20. Substitution counts of SARS-CoV-2 genomes through 14 February 2020 from the root of the maximum likelihood tree when rooted on lineage A (Fig. S19). The plotted lines have a slope of 27.51 substitutions/year, are fit to their respective lineages, and are separated by 2.04 substitutions, showcasing the greater divergence of lineage B than lineage A when the tree is rooted on lineage A.

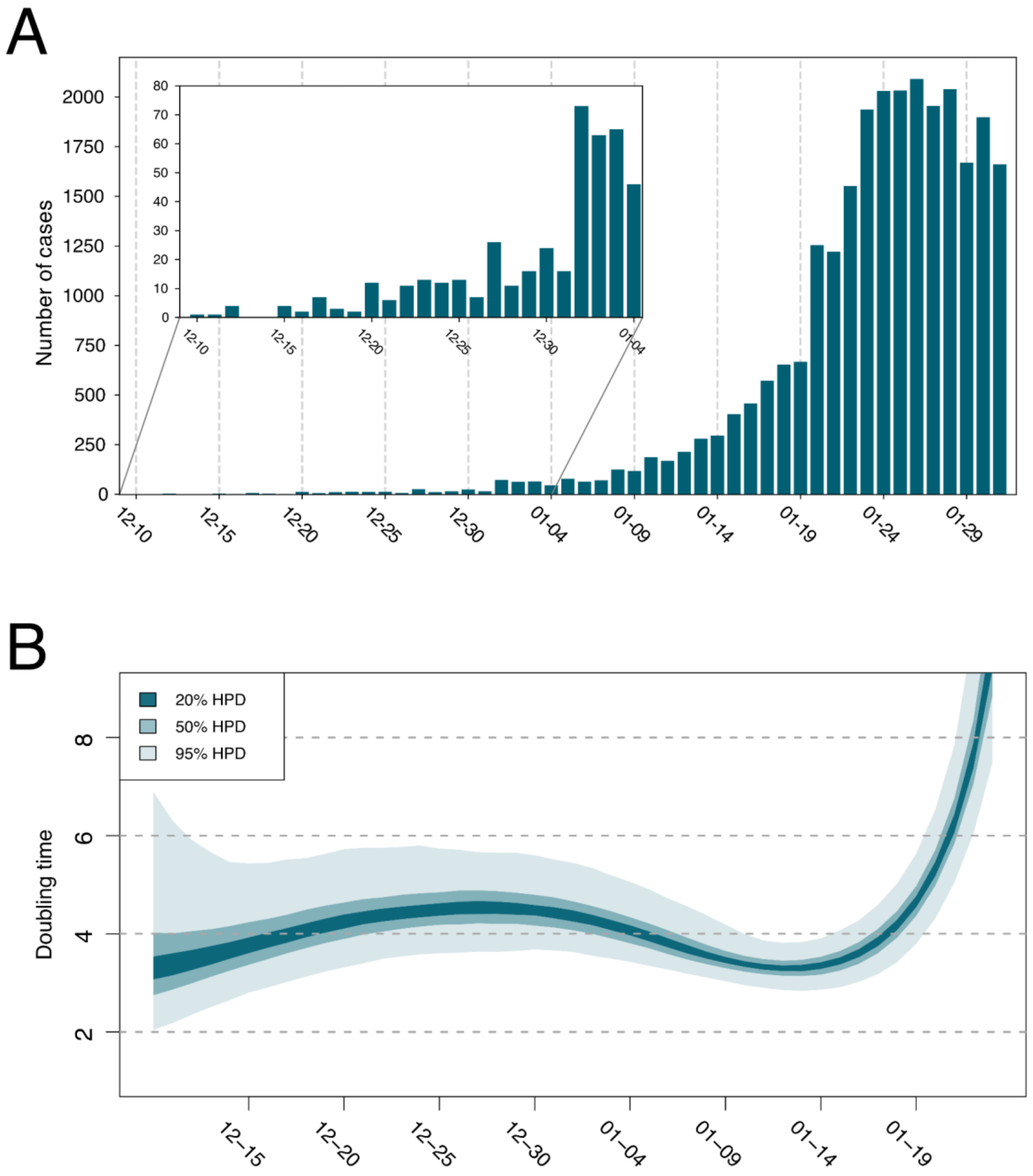

Figure S21. Growth of the early pandemic. (A) Daily case count after combining the data from the WHO report (*34*) and Li et al. (*80*) through January 2020. Inset shows daily case count through 4 January 2020. See Methods for how data were combined. **(B)** Inferred doubling times of the pandemic in December 2019 and January 2020.

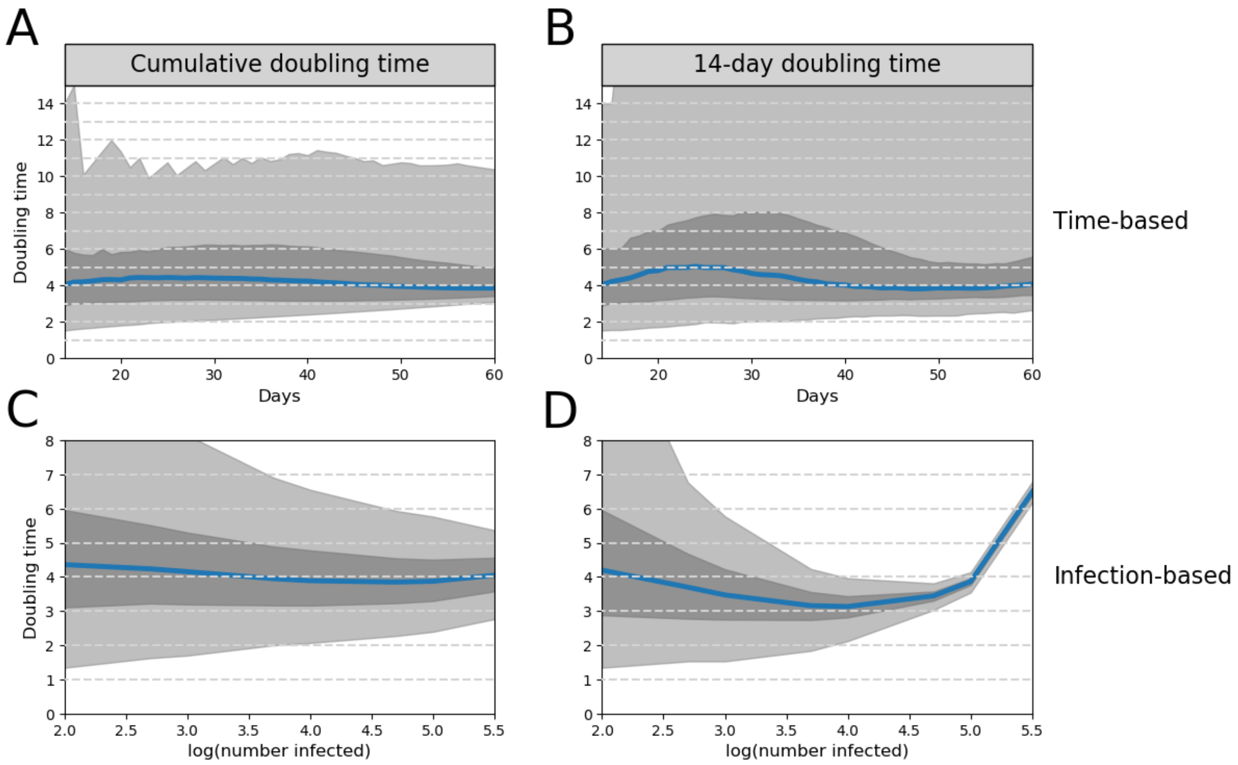

Figure S22. Inferred doubling times of simulated epidemics. Inferred doubling times of the 1100 primary simulations. (**A**) Cumulative doubling time since the start of the simulation. (**B**) 14-day doubling time from day 14 until the end of the simulation, with cumulative doubling time reported prior to day 14. (**C**) cumulative doubling time once a certain number of individuals are infected (*e.g.*, the cumulative doubling time at the 100th infection). (**D**) 14-day doubling time once a certain number of individuals are infected, with cumulative doubling time reported if that number of infections occurred before day 14 in the simulation. The center blue line represents the median doubling time across the simulations. Darker and lighter shading represent the 50% and 95% HDI, respectively.

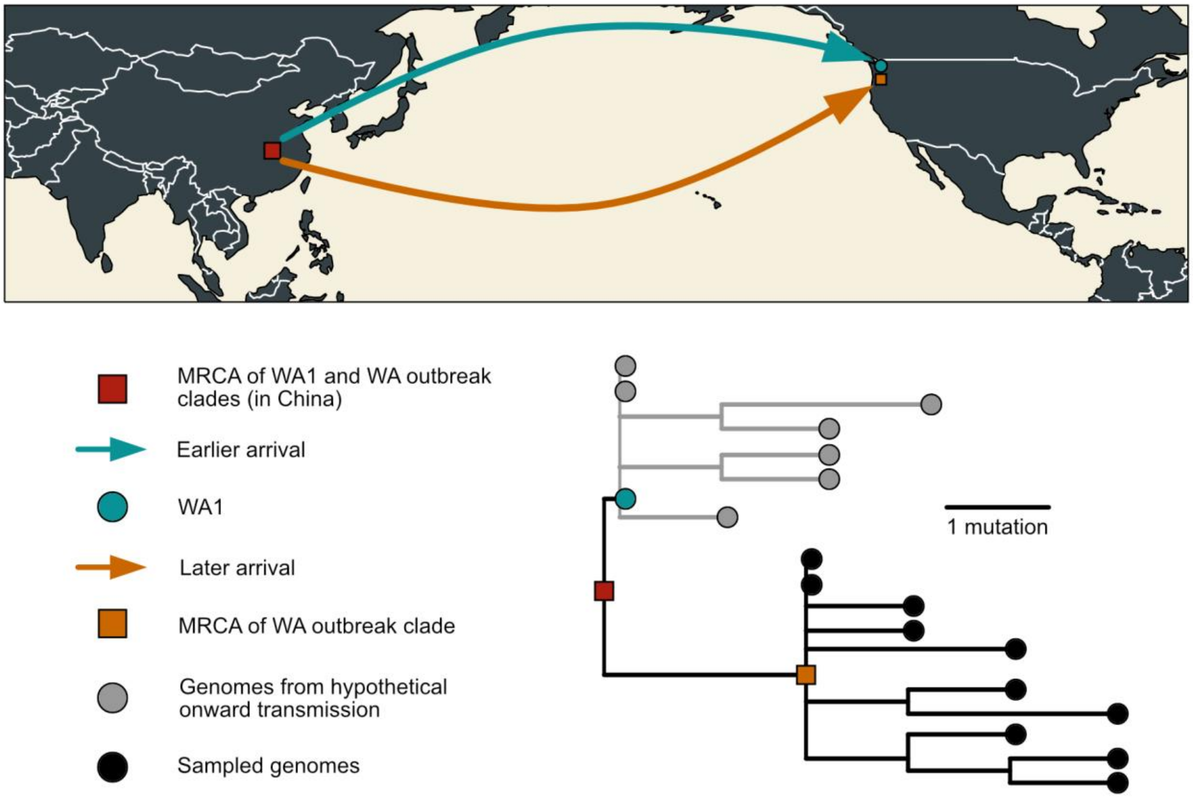

Figure S23. Early SARS-CoV-2 introductions into Washington state. Similar phylogenetic structure to the origins of SARS-CoV-2 in Wuhan is observed in Washington state, with two separate introductions of SARS-CoV-2 from China differing by two mutations (with no intermediate genomes). Refer to the supplementary text for a discussion comparing the introductions to Washington State with the origins of SARS-CoV-2.

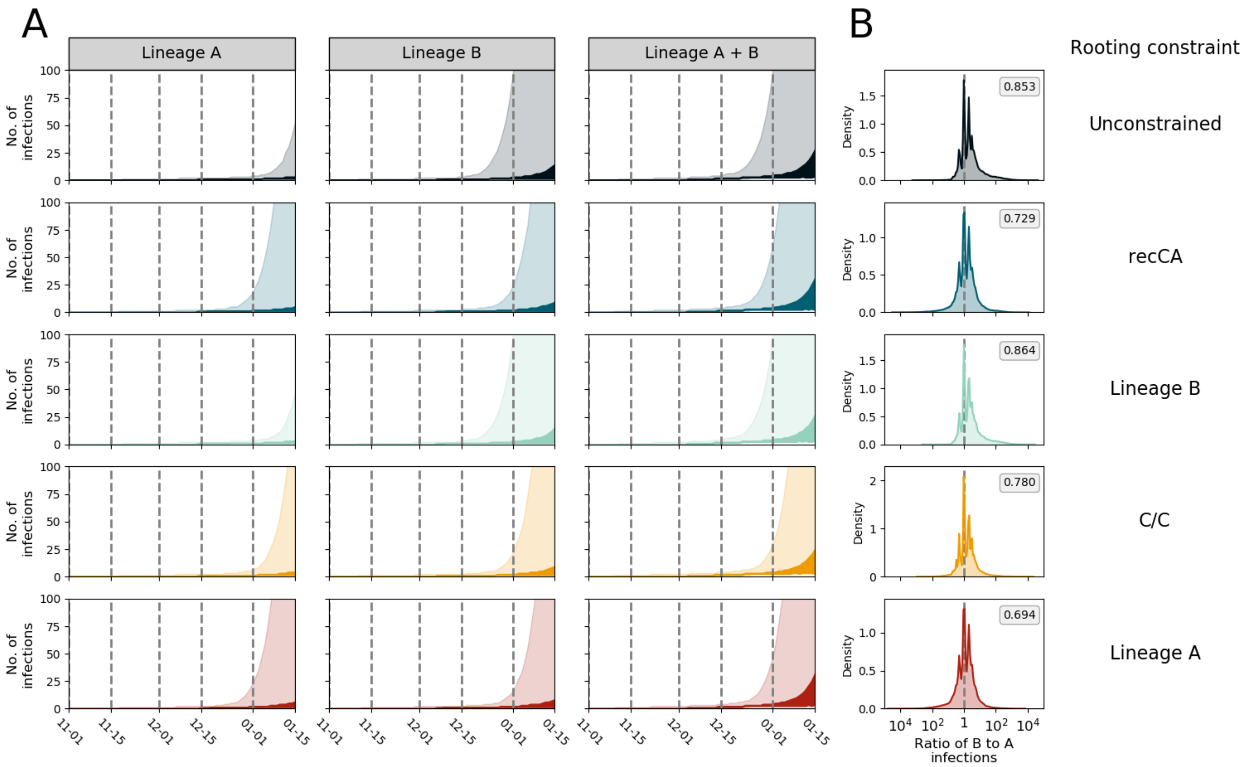

Figure S24. Dynamics of COVID-19 hospitalizations resulting from separate introductions of lineages A and B. Each row represents a different rooting constraint in phylodynamic analysis, with lineage B, C/C, and lineage A representing a fixed ancestral haplotype. (**A**) Estimated number of hospitalizations. The header of each column indicates whether the number of infections are caused by lineage A, lineage B, or the two lineages combined. Darker and lighter shading represent the 50% and 95% HPD, respectively. (**B**) The log ratio of lineage B to lineage A infections on 1 January 2020. Posterior probability of having more lineage B hospitalizations than lineage A reported in the grey box.

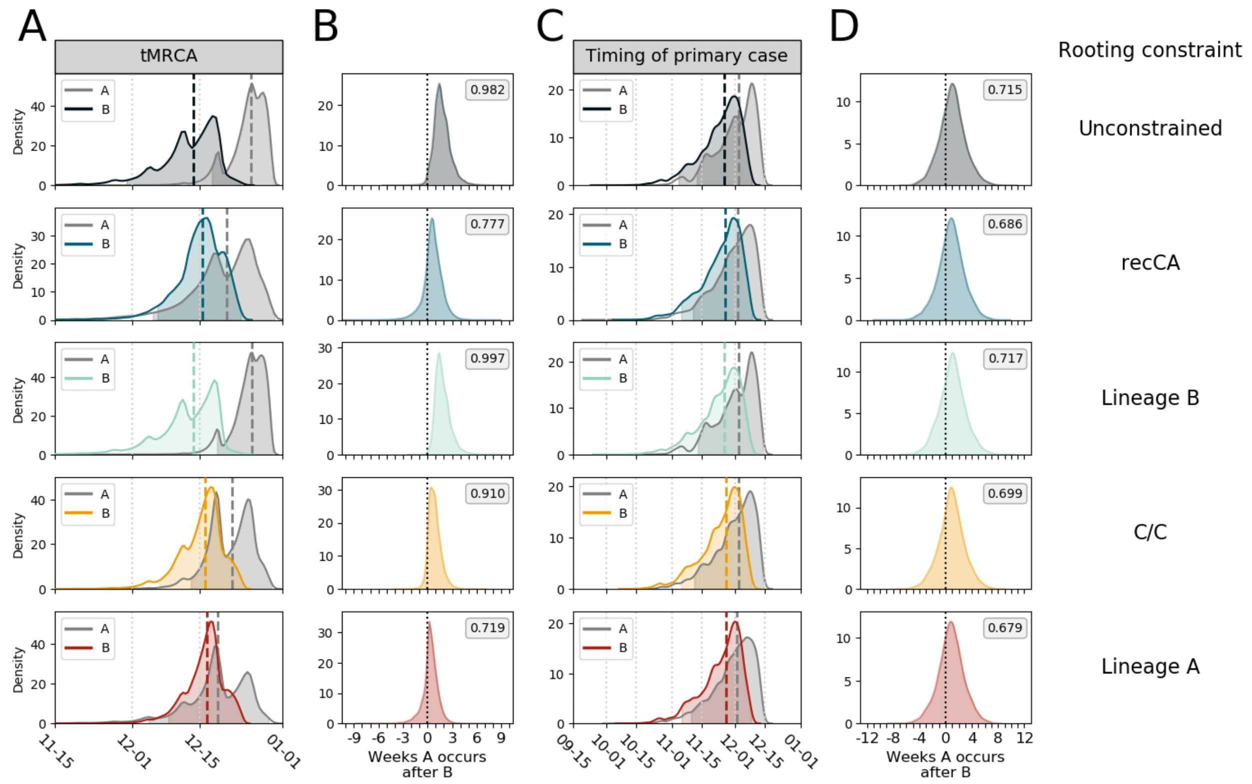

Figure S25. The timing of the MRCA and primary case for lineage A and lineage B with a shorter doubling time. The simulations used here have a doubling time of 2.65 days (95% HDI: 1.50-4.10). Each row represents a different rooting constraint in phylodynamic analysis, with lineage B, C/C, and lineage A representing a fixed ancestral haplotype. (**A**) The tMRCA for lineages A and B. (**B**) The number of weeks the tMRCA of lineage A occurs after the tMRCA of lineage B. (**C**) The timing of the primary case for lineages A and B. (**D**) The number of weeks the time of the primary case of lineage A occurs after the time of the primary case of lineage B. Long dashed lines indicate the median and shading represents the 95% HPD for each distribution. Short dashed lines indicate 0 weeks difference between lineages A and B. Posterior probability that lineage A originated after lineage B is reported in the grey box.

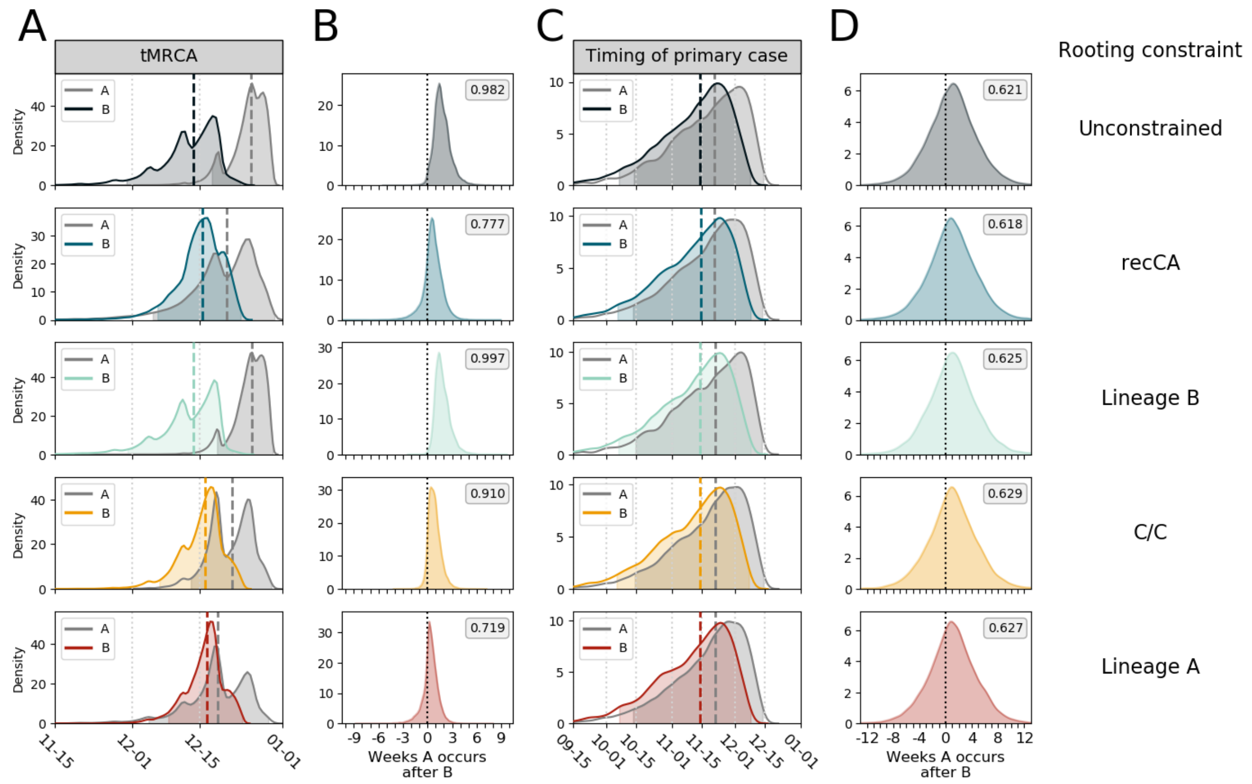

Figure S26. The timing of the MRCA and primary case for lineage A and lineage B with a longer doubling time. The simulations used here have a doubling time of 4.45 days (95%: HDI: 1.50-7.44). Each row represents a different rooting constraint in phylodynamic analysis, with lineage B, C/C, and lineage A representing a fixed ancestral haplotype. (**A**) The tMRCA for lineages A and B. (**B**) The number of weeks the tMRCA of lineage A occurs after the tMRCA of lineage B. (**C**) The timing of the primary case for lineages A and B. (**D**) The number of weeks the time of the primary case of lineage A occurs after the time of the primary case of lineage B. Long dashed lines indicate the median and shading represents the 95% HPD for each distribution. Short dashed lines indicate 0 weeks difference between lineages A and B. Posterior probability that lineage A originated after lineage B is reported in the grey box.

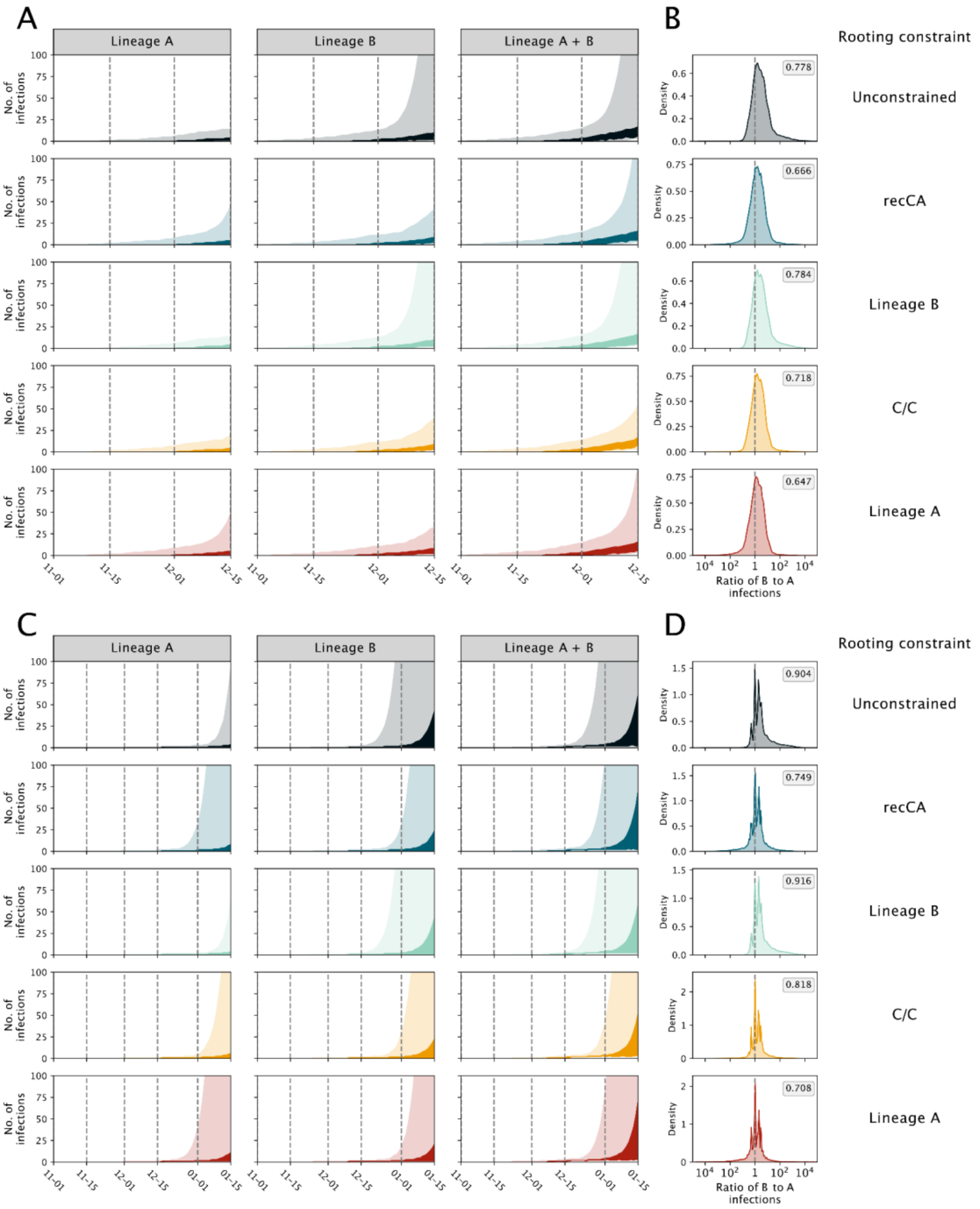

Figure S27. Dynamics of SARS-CoV-2 resulting from separate introductions of lineages A and B and a shorter doubling time. The simulations used here have a doubling time of 2.65 days (95% HDI: 1.50-4.10). Each row represents a different phylodynamic analysis, with lineage B, C/C, and lineage A representing an enforced ancestral haplotype. (**A**) Estimated number of infections. The header of each column indicates whether the infections are caused by lineage A, lineage B, or the two lineages combined. Darker and lighter shading represent the 50% and 95% HPD, respectively. (**B**) The log ratio of lineage B to lineage A infections on 15 December 2019. (**C**) Estimated number of hospitalizations, with column headers and shading identical to (**A**). (**D**) The log ratio of lineage B to lineage A hospitalizations on 1 January 2020. The proportion of the posterior with more lineage B infections or hospitalizations than lineage A in (**B**, **D**) is reported in the grey box.

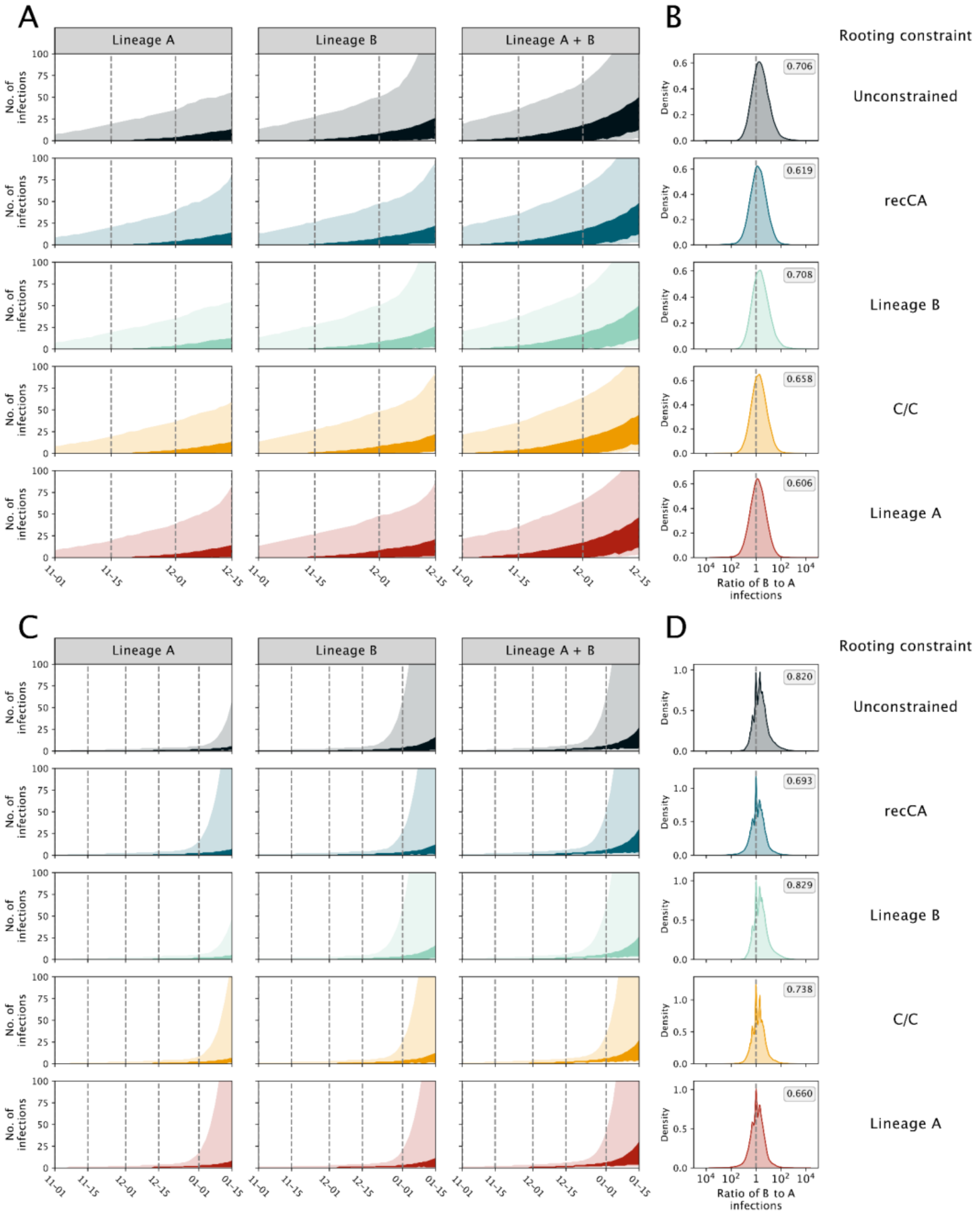

Figure S28. Dynamics of SARS-CoV-2 resulting from separate introductions of lineages A and B and a longer doubling time. The simulations used here have a doubling time of 4.45 days (95%: HDI: 1.50-7.44). Each row represents a different phylodynamic analysis, with lineage B, C/C, and lineage A representing an enforced ancestral haplotype. (**A**) Estimated number of infections. The header of each column indicates whether the infections are caused by lineage A, lineage B, or the two lineages combined. Darker and lighter shading represent the 50% and 95% HPD, respectively. (**B**) The log ratio of lineage B to lineage A infections on 15 December 2019. (**C**) Estimated number of hospitalizations, with column headers and shading identical to (**A**). (**D**) The log ratio of lineage B to lineage A hospitalizations on 1 January 2020. The proportion of the posterior with more lineage B infections or hospitalizations than lineage A in (**B**, **D**) is reported in the grey box.

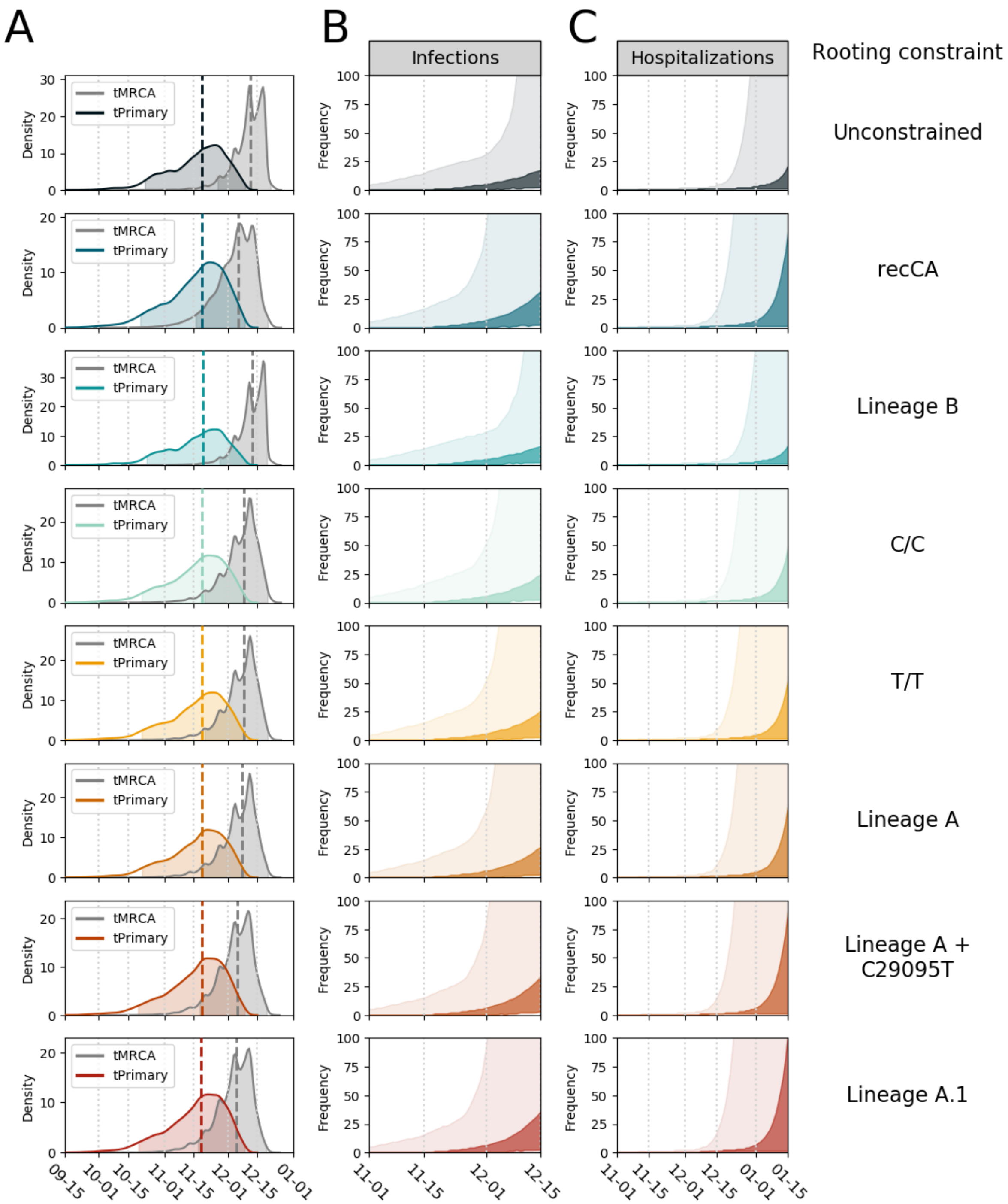

Figure S29. Single-introduction timing of the MRCA and primary case and subsequent epidemic growth. (**A**) Posterior distributions of the timing of the MRCA (tMRCA) and primary case (tPrimary), with dashed lines indicating the median and shading representing the 95% HPD for each distribution. (**B**) Estimated number of infections in late 2019. Darker shading represents 50% HPD; lighter shading represents 95% HPD. (**C**) Estimated number of hospitalizations in late 2019. The legend indicates the phylodynamic model used: the unconstrained model uses just the SARS-CoV-2 genomes; the recCA-constrained model constrains the ancestor of the MRCA of SARS-CoV-2 as the recCA; the remaining models constrain the MRCA of SARS-CoV-2 as a particular sequence (Fig. S20; see methods).

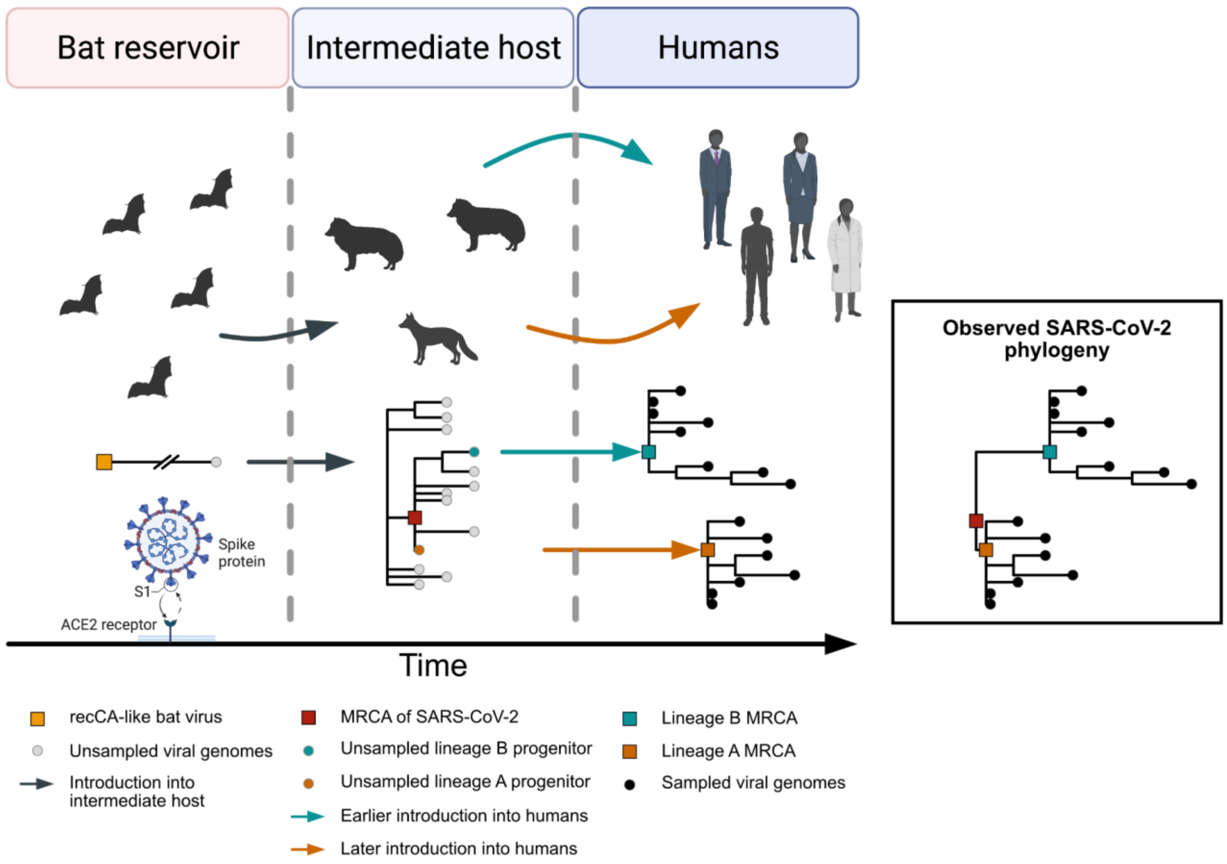

Figure S30. Schematic depicting the multiple zoonotic origin of SARS-CoV-2. A recCA-like virus was circulating in bats, and likely after gaining the ability to bind ACE2, jumped into an intermediate host. Therein, lineages A and B appeared and were separately introduced into humans shortly thereafter. An example phylogeny of viruses in the intermediate host is depicted, leading to separate phylogenies for lineages A and B. The resulting SARS-CoV-2 phylogeny from the combined lineage A and B viruses is presented in the black box. This scenario depicts a lineage A ancestral haplotype. See Figure S31 for intermediate and lineage B ancestral haplotypes.

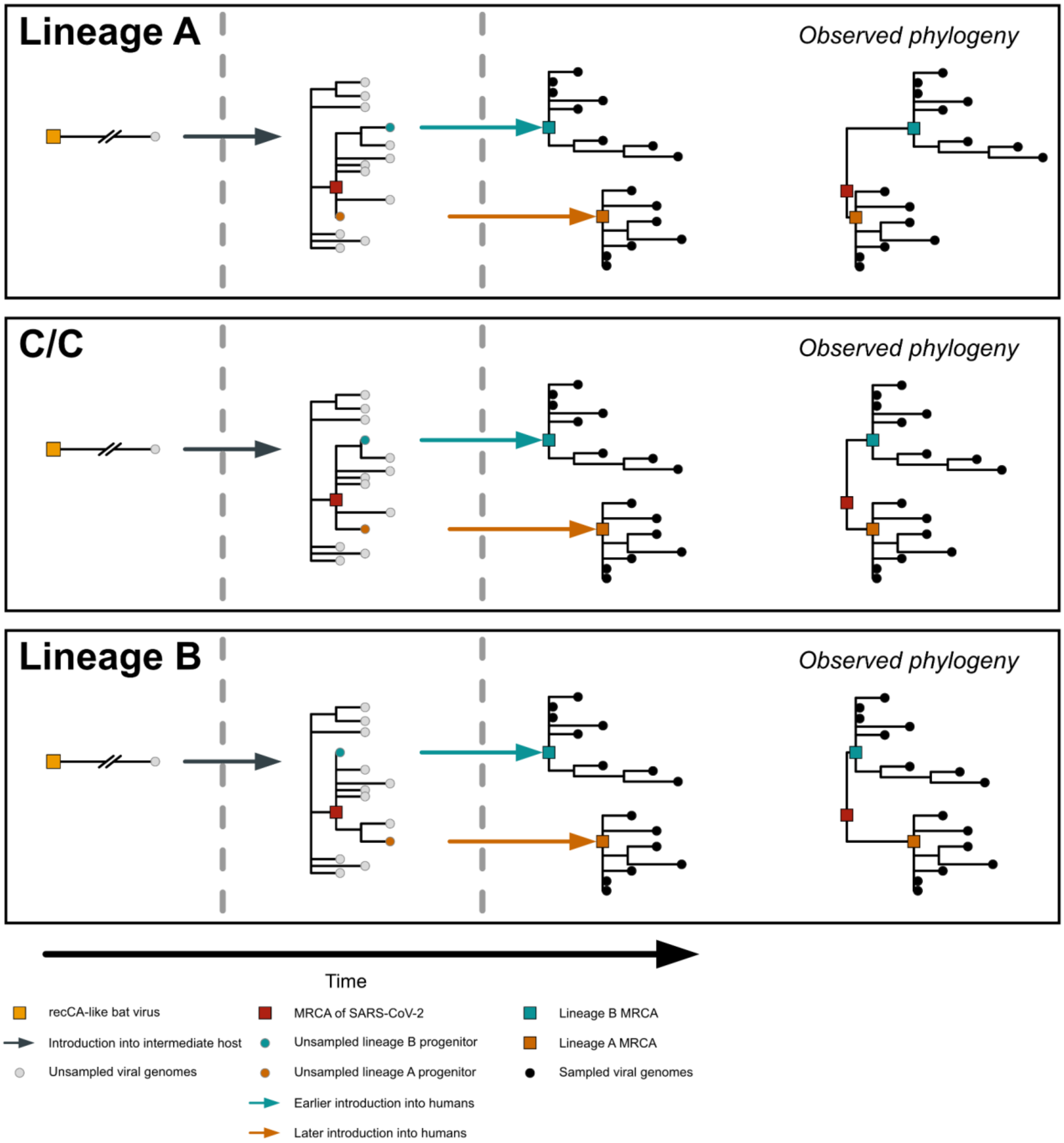

Figure S31. Rooting orientations of observed SARS-CoV-2 phylogenies resulting from different MRCAs and multiple introductions from the intermediate host. See Figure S30 for host depictions. The haplotype of the MRCA (red square in the left-center panel) is depicted in the upper left of each box.

References and Notes

1. E. Dong, H. Du, L. Gardner, An interactive web-based dashboard to track COVID-19 in real time. *Lancet Infect. Dis.* **20**, 533–534 (2020). doi:10.1016/S1473-3099(20)30120-1 Medline

2. L.-L. Ren, Y.-M. Wang, Z.-Q. Wu, Z.-C. Xiang, L. Guo, T. Xu, Y.-Z. Jiang, Y. Xiong, Y.-J. Li, X.-W. Li, H. Li, G.-H. Fan, X.-Y. Gu, Y. Xiao, H. Gao, J.-Y. Xu, F. Yang, X.-M. Wang, C. Wu, L. Chen, Y.-W. Liu, B. Liu, J. Yang, X.-R. Wang, J. Dong, L. Li, C.-L. Huang, J.-P. Zhao, Y. Hu, Z.-S. Cheng, L.-L. Liu, Z.-H. Qian, C. Qin, Q. Jin, B. Cao, J.-W. Wang, Identification of a novel coronavirus causing severe pneumonia in human: A descriptive study. *Chin. Med. J. (Engl.)* **133**, 1015–1024 (2020). doi:10.1097/CM9.0000000000000722 Medline

3. H. Ritchie, E. Mathieu, L. Rodés-Guirao, C. Appel, C. Giattino, E. Ortiz-Ospina, J. Hasell, B. Macdonald, S. Beltekian, X. Roser, Coronavirus Pandemic (COVID-19). *Our World in Data* (2022); https://ourworldindata.org/covid-deaths.

4. A. Rambaut, E. C. Holmes, Á. O'Toole, V. Hill, J. T. McCrone, C. Ruis, L. du Plessis, O. G. Pybus, A dynamic nomenclature proposal for SARS-CoV-2 lineages to assist genomic epidemiology. *Nat. Microbiol.* **5**, 1403–1407 (2020). doi:10.1038/s41564-020-0770-5 Medline

5. F. Wu, S. Zhao, B. Yu, Y.-M. Chen, W. Wang, Z.-G. Song, Y. Hu, Z.-W. Tao, J.-H. Tian, Y.-Y. Pei, M.-L. Yuan, Y.-L. Zhang, F.-H. Dai, Y. Liu, Q.-M. Wang, J.-J. Zheng, L. Xu, E. C. Holmes, Y.-Z. Zhang, A new coronavirus associated with human respiratory disease in China. *Nature* **579**, 265–269 (2020). doi:10.1038/s41586-020-2008-3 Medline

6. R. Lu, X. Zhao, J. Li, P. Niu, B. Yang, H. Wu, W. Wang, H. Song, B. Huang, N. Zhu, Y. Bi, X. Ma, F. Zhan, L. Wang, T. Hu, H. Zhou, Z. Hu, W. Zhou, L. Zhao, J. Chen, Y. Meng, J. Wang, Y. Lin, J. Yuan, Z. Xie, J. Ma, W. J. Liu, D. Wang, W. Xu, E. C. Holmes, G. F. Gao, G. Wu, W. Chen, W. Shi, W. Tan, Genomic characterisation and epidemiology of 2019 novel coronavirus: Implications for virus origins and receptor binding. *Lancet* **395**, 565–574 (2020). doi:10.1016/S0140-6736(20)30251-8 Medline

7. S. Lytras, J. Hughes, D. Martin, P. Swanepoel, A. de Klerk, R. Lourens, S. L. Kosakovsky Pond, W. Xia, X. Jiang, D. L. Robertson, Exploring the natural origins of SARS-CoV-2 in the light of recombination. *Genome Biol. Evol.* **14**, evac018 (2022). doi:10.1093/gbe/evac018 Medline

8. M. Worobey, Dissecting the early COVID-19 cases in Wuhan. *Science* **374**, 1202–1204 (2021). doi:10.1126/science.abm4454 Medline

9. R. F. Garry, Early appearance of two distinct genomic lineages of SARS-CoV-2 in different Wuhan wildlife markets suggests SARS-CoV-2 has a natural origin. *Virological* (2021); https://virological.org/t/early-appearance-of-two-distinct-genomic-lineages-of-sars-cov-2-in-different-wuhan-wildlife-markets-suggests-sars-cov-2-has-a-natural-origin/691.

10. N. De Maio, C. Walker, R. Borges, L. Weilguny, G. Slodkowicz, N. Goldman, Issues with SARS-CoV-2 sequencing data. *Virological* (2020); https://virological.org/t/issues-with-sars-cov-2-sequencing-data/473.

11. M. Worobey, J. Pekar, B. B. Larsen, M. I. Nelson, V. Hill, J. B. Joy, A. Rambaut, M. A. Suchard, J. O. Wertheim, P. Lemey, The emergence of SARS-CoV-2 in Europe and North America. *Science* **370**, 564–570 (2020). doi:10.1126/science.abc8169 Medline

12. J. O. Wertheim, M. Steel, M. J. Sanderson, Accuracy in Near-Perfect Virus Phylogenies. *Syst. Biol.* **71**, 426–438 (2022). doi:10.1093/sysbio/syab069 Medline

13. S. Temmam, K. Vongphayloth, E. Baquero, S. Munier, M. Bonomi, B. Regnault, B. Douangboubpha, Y. Karami, D. Chrétien, D. Sanamxay, V. Xayaphet, P. Paphaphanh, V. Lacoste, S. Somlor, K. Lakeomany, N. Phommavanh, P. Pérot, O. Dehan, F. Amara, F. Donati, T. Bigot, M. Nilges, F. A. Rey, S. van der Werf, P. T. Brey, M. Eloit, Bat coronaviruses related to SARS-CoV-2 and infectious for human cells. *Nature* **604**, 330–336 (2022). doi:10.1038/s41586-022-04532-4 Medline

14. J. B. Pease, M. W. Hahn, More accurate phylogenies inferred from low-recombination regions in the presence of incomplete lineage sorting. *Evolution* **67**, 2376–2384 (2013). doi:10.1111/evo.12118 Medline

15. J. Ratcliff, P. Simmonds, Potential APOBEC-mediated RNA editing of the genomes of SARS-CoV-2 and other coronaviruses and its impact on their longer term evolution. *Virology* **556**, 62–72 (2021). doi:10.1016/j.virol.2020.12.018 Medline

16. P. Simmonds, Rampant C→U Hypermutation in the Genomes of SARS-CoV-2 and Other Coronaviruses: Causes and Consequences for Their Short- and Long-Term Evolutionary Trajectories. *MSphere* **5**, e00408-20 (2020). doi:10.1128/mSphere.00408-20 Medline

17. P. Simmonds, M. A. Ansari, Extensive C->U transition biases in the genomes of a wide range of mammalian RNA viruses; potential associations with transcriptional mutations, damage- or host-mediated editing of viral RNA. *PLOS Pathog.* **17**, e1009596 (2021). doi:10.1371/journal.ppat.1009596 Medline

18. P. Forster, L. Forster, C. Renfrew, M. Forster, Phylogenetic network analysis of SARS-CoV-2 genomes. *Proc. Natl. Acad. Sci. U.S.A.* **117**, 9241–9243 (2020). doi:10.1073/pnas.2004999117 Medline

19. J. D. Bloom, Recovery of Deleted Deep Sequencing Data Sheds More Light on the Early Wuhan SARS-CoV-2 Epidemic. *Mol. Biol. Evol.* **38**, 5211–5224 (2021). doi:10.1093/molbev/msab246 Medline

20. M. A. Caraballo-Ortiz, S. Miura, M. Sanderford, T. Dolker, Q. Tao, S. Weaver, S. L. K. Pond, S. Kumar, TopHap: Rapid inference of key phylogenetic structures from common haplotypes in large genome collections with limited diversity. *Bioinformatics* **38**, 2719–2726 (2022). doi:10.1093/bioinformatics/btac186 Medline

21. S. Kumar, Q. Tao, S. Weaver, M. Sanderford, M. A. Caraballo-Ortiz, S. Sharma, S. L. K. Pond, S. Miura, An Evolutionary Portrait of the Progenitor SARS-CoV-2 and Its Dominant Offshoots in COVID-19 Pandemic. *Mol. Biol. Evol.* **38**, 3046–3059 (2021). doi:10.1093/molbev/msab118 Medline

22. N. Moshiri, M. Ragonnet-Cronin, J. O. Wertheim, S. Mirarab, FAVITES: Simultaneous simulation of transmission networks, phylogenetic trees and sequences. *Bioinformatics* **35**, 1852–1861 (2019). doi:10.1093/bioinformatics/bty921 Medline

23. J. Pekar, M. Worobey, N. Moshiri, K. Scheffler, J. O. Wertheim, Timing the SARS-CoV-2 index case in Hubei province. *Science* **372**, 412–417 (2021). doi:10.1126/science.abf8003 Medline

24. S. Hsiang, D. Allen, S. Annan-Phan, K. Bell, I. Bolliger, T. Chong, H. Druckenmiller, L. Y. Huang, A. Hultgren, E. Krasovich, P. Lau, J. Lee, E. Rolf, J. Tseng, T. Wu, The effect of large-scale anti-contagion policies on the COVID-19 pandemic. *Nature* **584**, 262–267 (2020). doi:10.1038/s41586-020-2404-8 Medline

25. A. L. Bertozzi, E. Franco, G. Mohler, M. B. Short, D. Sledge, The challenges of modeling and forecasting the spread of COVID-19. *Proc. Natl. Acad. Sci. U.S.A.* **117**, 16732–16738 (2020). doi:10.1073/pnas.2006520117 Medline

26. S. Sanche, Y. T. Lin, C. Xu, E. Romero-Severson, N. Hengartner, R. Ke, High Contagiousness and Rapid Spread of Severe Acute Respiratory Syndrome Coronavirus 2. *Emerg. Infect. Dis.* **26**, 1470–1477 (2020). doi:10.3201/eid2607.200282 Medline

27. T. Bedford, A. L. Greninger, P. Roychoudhury, L. M. Starita, M. Famulare, M.-L. Huang, A. Nalla, G. Pepper, A. Reinhardt, H. Xie, L. Shrestha, T. N. Nguyen, A. Adler, E. Brandstetter, S. Cho, D. Giroux, P. D. Han, K. Fay, C. D. Frazar, M. Ilcisin, K. Lacombe, J. Lee, A. Kiavand, M. Richardson, T. R. Sibley, M. Truong, C. R. Wolf, D. A. Nickerson, M. J. Rieder, J. A. Englund, J. Hadfield, E. B. Hodcroft, J. Huddleston, L. H. Moncla, N. F. Müller, R. A. Neher, X. Deng, W. Gu, S. Federman, C. Chiu, J. S. Duchin, R. Gautom, G. Melly, B. Hiatt, P. Dykema, S. Lindquist, K. Queen, Y. Tao, A. Uehara, S. Tong, D. MacCannell, G. L. Armstrong, G. S. Baird, H. Y. Chu, J. Shendure, K. R. Jerome, H. Y. Chu, M. Boeckh, J. A. Englund, M. Famulare, B. R. Lutz, D. A. Nickerson, M. J. Rieder, L. M. Starita, M. Thompson, J. Shendure, T. Bedford, A. Adler, E. Brandstetter, S. Cho, C. D. Frazar, D. Giroux, P. D. Han, J. Hadfield, S. Huang, M. L. Jackson, A. Kiavand, L. E. Kimball, K. Lacombe, J. Logue, V. Lyon, K. L. Newman, M. Richardson, T. R. Sibley, M. L. Zigman Suchsland, M. Truong, C. R. Wolf, Seattle Flu Study Investigators, Cryptic transmission of SARS-CoV-2 in Washington state. *Science* **370**, 571–575 (2020). doi:10.1126/science.abc0523 Medline

28. M. Zeller, K. Gangavarapu, C. Anderson, A. R. Smither, J. A. Vanchiere, R. Rose, D. J. Snyder, G. Dudas, A. Watts, N. L. Matteson, R. Robles-Sikisaka, M. Marshall, A. K. Feehan, G. Sabino-Santos Jr., A. R. Bell-Kareem, L. D. Hughes, M. Alkuzweny, P. Snarski, J. Garcia-Diaz, R. S. Scott, L. I. Melnik, R. Klitting, M. McGraw, P. Belda-Ferre, P. DeHoff, S. Sathe, C. Marotz, N. D. Grubaugh, D. J. Nolan, A. C. Drouin, K. J. Genemaras, K. Chao, S. Topol, E. Spencer, L. Nicholson, S. Aigner, G. W. Yeo, L. Farnaes, C. A. Hobbs, L. C. Laurent, R. Knight, E. B. Hodcroft, K. Khan, D. N. Fusco, V. S. Cooper, P. Lemey, L. Gardner, S. L. Lamers, J. P. Kamil, R. F. Garry, M. A. Suchard, K. G. Andersen, Emergence of an early SARS-CoV-2 epidemic in the United States. *Cell* **184**, 4939–4952.e15 (2021). doi:10.1016/j.cell.2021.07.030 Medline

29. C. Alteri, V. Cento, A. Piralla, V. Costabile, M. Tallarita, L. Colagrossi, S. Renica, F. Giardina, F. Novazzi, S. Gaiarsa, E. Matarazzo, M. Antonello, C. Vismara, R. Fumagalli, O. M. Epis, M. Puoti, C. F. Perno, F. Baldanti, Genomic epidemiology of SARS-CoV-2 reveals multiple lineages and early spread of SARS-CoV-2 infections in Lombardy, Italy. *Nat. Commun.* **12**, 434 (2021). doi:10.1038/s41467-020-20688-x Medline

30. L. du Plessis, O. Pybus, Further musings on the tMRCA. *Virological* (2020); https://virological.org/t/further-musings-on-the-tmrca/340.

31. J. Giesecke, Primary and index cases. *Lancet* **384**, 2024 (2014). doi:10.1016/S0140-6736(14)62331-X Medline

32. Centers for Disease Control and Prevention (CDC), Prevalence of IgG antibody to SARS-associated coronavirus in animal traders—Guangdong Province, China, 2003. *MMWR Morb. Mortal. Wkly. Rep.* **52**, 986–987 (2003). Medline

33. A. Marí Saéz, S. Weiss, K. Nowak, V. Lapeyre, F. Zimmermann, A. Düx, H. S. Kühl, M. Kaba, S. Regnaut, K. Merkel, A. Sachse, U. Thiesen, L. Villányi, C. Boesch, P. W. Dabrowski, A. Radonić, A. Nitsche, S. A. J. Leendertz, S. Petterson, S. Becker, V. Krähling, E. Couacy-Hymann, C. Akoua-Koffi, N. Weber, L. Schaade, J. Fahr, M. Borchert, J. F. Gogarten, S. Calvignac-Spencer, F. H. Leendertz, Investigating the zoonotic origin of the West African Ebola epidemic. *EMBO Mol. Med.* **7**, 17–23 (2015). doi:10.15252/emmm.201404792 Medline

34. WHO Headquarters, WHO-convened global study of origins of SARS-CoV-2: China Part (2021); https://www.who.int/publications/i/item/who-convened-global-study-of-origins-of-sars-cov-2-china-part.

35. X. Zhang, Y. Tan, Y. Ling, G. Lu, F. Liu, Z. Yi, X. Jia, M. Wu, B. Shi, S. Xu, J. Chen, W. Wang, B. Chen, L. Jiang, S. Yu, J. Lu, J. Wang, M. Xu, Z. Yuan, Q. Zhang, X. Zhang, G. Zhao, S. Wang, S. Chen, H. Lu, Viral and host factors related to the clinical outcome of COVID-19. *Nature* **583**, 437–440 (2020). doi:10.1038/s41586-020-2355-0 Medline

36. E. O. Nsoesie, B. Rader, Y. L. Barnoon, L. Goodwin, J. Brownstein, Analysis of hospital traffic and search engine data in Wuhan China indicates early disease activity in the Fall of 2019. *Dig. Acc. Scholar. Harv.* **2**, 019 (2020).

37. L. Chang, L. Zhao, Y. Xiao, T. Xu, L. Chen, Y. Cai, X. Dong, C. Wang, X. Xiao, L. Ren, L. Wang, Serosurvey for SARS-CoV-2 among blood donors in Wuhan, China from September to December 2019. *Protein Cell* 10.1093/procel/pwac013 (2022).

38. E. C. Holmes, S. A. Goldstein, A. L. Rasmussen, D. L. Robertson, A. Crits-Christoph, J. O. Wertheim, S. J. Anthony, W. S. Barclay, M. F. Boni, P. C. Doherty, J. Farrar, J. L. Geoghegan, X. Jiang, J. L. Leibowitz, S. J. D. Neil, T. Skern, S. R. Weiss, M. Worobey, K. G. Andersen, R. F. Garry, A. Rambaut, The origins of SARS-CoV-2: A critical review. *Cell* **184**, 4848–4856 (2021). doi:10.1016/j.cell.2021.08.017 Medline

39. M. Worobey, J. I. Levy, L. M. Malpica Serrano, A. Crits-Christoph, J. E. Pekar, S. A. Goldstein, A. L. Rasmussen, M. U. G. Kraemer, C. Newman, M. P. G. Koopmans, M. A. Suchard, J. O. Wertheim, P. Lemey, D. L. Robertson, R. F. Garry, E. C. Holmes, A. Rambaut, K. G. Andersen, The Huanan market was the epicenter of SARS-CoV-2 emergence. Zenodo (2022); https://zenodo.org/record/6299116.

40. X. Xiao, C. Newman, C. D. Buesching, D. W. Macdonald, Z.-M. Zhou, Animal sales from Wuhan wet markets immediately prior to the COVID-19 pandemic. *Sci. Rep.* **11**, 11898 (2021). doi:10.1038/s41598-021-91470-2 Medline

41. C. M. Freuling, A. Breithaupt, T. Müller, J. Sehl, A. Balkema-Buschmann, M. Rissmann, A. Klein, C. Wylezich, D. Höper, K. Wernike, A. Aebischer, D. Hoffmann, V. Friedrichs, A.

Dorhoi, M. H. Groschup, M. Beer, T. C. Mettenleiter, Susceptibility of Raccoon Dogs for Experimental SARS-CoV-2 Infection. *Emerg. Infect. Dis.* **26**, 2982–2985 (2020). doi:10.3201/eid2612.203733 Medline

42. S. M. Porter, A. E. Hartwig, H. Bielefeldt-Ohmann, A. M. Bosco-Lauth, J. Root, Susceptibility of wild canids to severe acute respiratory syndrome coronavirus 2 (SARS-CoV-2). bioRxiv 478082 [Preprint] (2022). https://doi.org/10.1101/2022.01.27.478082.

43. G. Gao, W. Liu, P. Liu, W. Lei, Z. Jia, X. He, L.-L. Liu, W. Shi, Y. Tan, S. Zou, X. Zhao, G. Wong, J. Wang, F. Wang, G. Wang, K. Qin, R. Gao, J. Zhang, M. Li, W. Xiao, Y. Guo, Z. Xu, Y. Zhao, J. Song, J. Zhang, W. Zhen, W. Zhou, B. Ye, J. Song, M. Yang, W. Zhou, Y. Bi, K. Cai, D. Wang, W. Tan, J. Han, W. Xu, G. Wu, Surveillance of SARS-CoV-2 in the environment and animal samples of the Huanan Seafood Market. Research Square (2022). https://doi.org/10.21203/rs.3.rs-1370392/v1.

44. L. du Plessis, J. T. McCrone, A. E. Zarebski, V. Hill, C. Ruis, B. Gutierrez, J. Raghwani, J. Ashworth, R. Colquhoun, T. R. Connor, N. R. Faria, B. Jackson, N. J. Loman, Á. O'Toole, S. M. Nicholls, K. V. Parag, E. Scher, T. I. Vasylyeva, E. M. Volz, A. Watts, I. I. Bogoch, K. Khan, D. M. Aanensen, M. U. G. Kraemer, A. Rambaut, O. G. Pybus; COVID-19 Genomics UK (COG-UK) Consortium, Establishment and lineage dynamics of the SARS-CoV-2 epidemic in the UK. *Science* **371**, 708–712 (2021). doi:10.1126/science.abf2946 Medline

45. Chinese SARS Molecular Epidemiology Consortium, Molecular evolution of the SARS coronavirus during the course of the SARS epidemic in China. *Science* **303**, 1666–1669 (2004). doi:10.1126/science.1092002 Medline

46. G. Dudas, L. M. Carvalho, A. Rambaut, T. Bedford, MERS-CoV spillover at the camel-human interface. *eLife* **7**, e31257 (2018). doi:10.7554/eLife.31257 Medline

47. J. A. Lednicky, M. S. Tagliamonte, S. K. White, M. A. Elbadry, M. M. Alam, C. J. Stephenson, T. S. Bonny, J. C. Loeb, T. Telisma, S. Chavannes, D. A. Ostrov, C. Mavian, V. M. Beau De Rochars, M. Salemi, J. G. Morris Jr., Independent infections of porcine deltacoronavirus among Haitian children. *Nature* **600**, 133–137 (2021). doi:10.1038/s41586-021-04111-z Medline

48. B. Kan, M. Wang, H. Jing, H. Xu, X. Jiang, M. Yan, W. Liang, H. Zheng, K. Wan, Q. Liu, B. Cui, Y. Xu, E. Zhang, H. Wang, J. Ye, G. Li, M. Li, Z. Cui, X. Qi, K. Chen, L. Du, K. Gao, Y.-T. Zhao, X.-Z. Zou, Y.-J. Feng, Y.-F. Gao, R. Hai, D. Yu, Y. Guan, J. Xu, Molecular evolution analysis and geographic investigation of severe acute respiratory syndrome coronavirus-like virus in palm civets at an animal market and on farms. *J. Virol.* **79**, 11892–11900 (2005). doi:10.1128/JVI.79.18.11892-11900.2005 Medline

49. K. G. Andersen, A. Rambaut, W. I. Lipkin, E. C. Holmes, R. F. Garry, The proximal origin of SARS-CoV-2. *Nat. Med.* **26**, 450–452 (2020). doi:10.1038/s41591-020-0820-9 Medline

50. V. L. Hale, P. M. Dennis, D. S. McBride, J. M. Nolting, C. Madden, D. Huey, M. Ehrlich, J. Grieser, J. Winston, D. Lombardi, S. Gibson, L. Saif, M. L. Killian, K. Lantz, R. M. Tell, M. Torchetti, S. Robbe-Austerman, M. I. Nelson, S. A. Faith, A. S. Bowman, SARS-CoV-2 infection in free-ranging white-tailed deer. *Nature* **602**, 481–486 (2022). doi:10.1038/s41586-021-04353-x Medline

51. J. C. Chandler, S. N. Bevins, J. W. Ellis, T. J. Linder, R. M. Tell, M. Jenkins-Moore, J. J. Root, J. B. Lenoch, S. Robbe-Austerman, T. J. DeLiberto, T. Gidlewski, M. Kim Torchetti, S. A. Shriner, SARS-CoV-2 exposure in wild white-tailed deer (*Odocoileus virginianus*). *Proc. Natl. Acad. Sci. U.S.A.* **118**, e2114828118 (2021). doi:10.1073/pnas.2114828118 Medline

52. L. Lu, R. S. Sikkema, F. C. Velkers, D. F. Nieuwenhuijse, E. A. J. Fischer, P. A. Meijer, N. Bouwmeester-Vincken, A. Rietveld, M. C. A. Wegdam-Blans, P. Tolsma, M. Koppelman, L. A. M. Smit, R. W. Hakze-van der Honing, W. H. M. van der Poel, A. N. van der Spek, M. A. H. Spierenburg, R. J. Molenaar, J. Rond, M. Augustijn, M. Woolhouse, J. A. Stegeman, S. Lycett, B. B. Oude Munnink, M. P. G. Koopmans, Adaptation, spread and transmission of SARS-CoV-2 in farmed minks and associated humans in the Netherlands. *Nat. Commun.* **12**, 6802 (2021). doi:10.1038/s41467-021-27096-9 Medline

53. B. B. Oude Munnink, R. S. Sikkema, D. F. Nieuwenhuijse, R. J. Molenaar, E. Munger, R. Molenkamp, A. van der Spek, P. Tolsma, A. Rietveld, M. Brouwer, N. Bouwmeester-Vincken, F. Harders, R. Hakze-van der Honing, M. C. A. Wegdam-Blans, R. J. Bouwstra, C. GeurtsvanKessel, A. A. van der Eijk, F. C. Velkers, L. A. M. Smit, A. Stegeman, W. H. M. van der Poel, M. P. G. Koopmans, Transmission of SARS-CoV-2 on mink farms between humans and mink and back to humans. *Science* **371**, 172–177 (2021). doi:10.1126/science.abe5901 Medline

54. S. V. Kuchipudi, M. Surendran-Nair, R. M. Ruden, M. Yon, R. H. Nissly, K. J. Vandegrift, R. K. Nelli, L. Li, B. M. Jayarao, C. D. Maranas, N. Levine, K. Willgert, A. J. K. Conlan, R. J. Olsen, J. J. Davis, J. M. Musser, P. J. Hudson, V. Kapur, Multiple spillovers from humans and onward transmission of SARS-CoV-2 in white-tailed deer. *Proc. Natl. Acad. Sci. U.S.A.* **119**, e2121644119 (2022). doi:10.1073/pnas.2121644119 Medline

55. H.-L. Yen, T. H. C. Sit, C. J. Brackman, S. S. Y. Chuk, S. M. S. Cheng, H. Gu, L. D. J. Chang, P. Krishnan, D. Y. M. Ng, G. Y. Z. Liu, M. M. Y. Hui, S. Y. Ho, K. W. S. Tam, P. Y. T. Law, W. Su, S. F. Sia, K.-T. Choy, S. S. Y. Cheuk, S. P. N. Lau, A. W. Y. Tang, J. C. T. Koo, L. Yung, G. Leung, J. S. M. Peiris, L. L. M. Poon, Transmission of SARS-CoV-2 delta variant (AY.127) from pet hamsters to humans, leading to onward human-to-human transmission: A case study. *Lancet* **399**, 1070–1078 (2022). doi:10.1016/S0140-6736(22)00326-9 Medline

56. H.-L. Yen, T. H. C. Sit, C. J. Brackman, S. S. Y. Chuk, H. Gu, K. W. S. Tam, P. Y. T. Law, G. M. Leung, M. Peiris, L. L. M. Poon, S. M. S. Cheng, L. D. J. Chang, P. Krishnan, D. Y. M. Ng, G. Y. Z. Liu, M. M. Y. Hui, S. Y. Ho, W. Su, S. F. Sia, K.-T. Choy, S. S. Y. Cheuk, S. P. N. Lau, A. W. Y. Tang, J. C. T. Koo, L. Yung; HKU-SPH study team, Transmission of SARS-CoV-2 delta variant (AY.127) from pet hamsters to humans, leading to onward human-to-human transmission: A case study. *Lancet* **399**, 1070–1078 (2022). doi:10.1016/S0140-6736(22)00326-9 Medline

57. Y. Shu, J. McCauley, GISAID: Global initiative on sharing all influenza data - from vision to reality. *Euro Surveill.* **22**, 30494 (2017). doi:10.2807/1560-7917.ES.2017.22.13.30494 Medline

58. K. Katoh, D. M. Standley, MAFFT multiple sequence alignment software version 7: Improvements in performance and usability. *Mol. Biol. Evol.* **30**, 772–780 (2013). doi:10.1093/molbev/mst010 Medline

59. N. De Maio, C. Walker, R. Borges, L. Weilguny, G. Slodkowicz, N. Goldman, Masking strategies for SARS-CoV-2 alignments. *Virological* (2020); https://virological.org/t/masking-strategies-for-sars-cov-2-alignments/480.

60. B. Q. Minh, H. A. Schmidt, O. Chernomor, D. Schrempf, M. D. Woodhams, A. von Haeseler, R. Lanfear, IQ-TREE 2: New Models and Efficient Methods for Phylogenetic Inference in the Genomic Era. *Mol. Biol. Evol.* **37**, 1530–1534 (2020). doi:10.1093/molbev/msaa015 Medline

61. P. Sagulenko, V. Puller, R. A. Neher, TreeTime: Maximum-likelihood phylodynamic analysis. *Virus Evol.* **4**, vex042 (2018). doi:10.1093/ve/vex042 Medline

62. M. A. Suchard, P. Lemey, G. Baele, D. L. Ayres, A. J. Drummond, A. Rambaut, Bayesian phylogenetic and phylodynamic data integration using BEAST 1.10. *Virus Evol.* **4**, vey016 (2018). doi:10.1093/ve/vey016 Medline

63. N. Moshiri, *FAVITES-COVID-Lite: A simplified (and much faster) simulation pipeline specifically for COVID-19 contact + transmission + phylogeny + sequence simulation* (Github, 2022); https://github.com/niemasd/FAVITES-COVID-Lite.

64. X. Hao, S. Cheng, D. Wu, T. Wu, X. Lin, C. Wang, Reconstruction of the full transmission dynamics of COVID-19 in Wuhan. *Nature* **584**, 420–424 (2020). doi:10.1038/s41586-020-2554-8 Medline

65. J. E. Pekar, A. Rambaut, sars-cov-2-origins/multi-introduction: v1.0.0. Zenodo (2022); doi:10.5281/zenodo.6585475.

66. J. E. Pekar, J. O. Wertheim, Data 1 for: The molecular epidemiology of multiple zoonotic transmissions of SARS-CoV-2. Zenodo (2022); 10.5281/zenodo.6887187.

67. J. Hadfield, C. Megill, S. M. Bell, J. Huddleston, B. Potter, C. Callender, P. Sagulenko, T. Bedford, R. A. Neher, Nextstrain: Real-time tracking of pathogen evolution. *Bioinformatics* **34**, 4121–4123 (2018). doi:10.1093/bioinformatics/bty407 Medline

68. A. Rambaut, *figtree* (Github, 2018); https://github.com/rambaut/figtree/releases.

69. H. Li, Minimap2: Pairwise alignment for nucleotide sequences. *Bioinformatics* **34**, 3094–3100 (2018). doi:10.1093/bioinformatics/bty191 Medline

70. H. Li, B. Handsaker, A. Wysoker, T. Fennell, J. Ruan, N. Homer, G. Marth, G. Abecasis, R. Durbin; 1000 Genome Project Data Processing Subgroup, The Sequence Alignment/Map format and SAMtools. *Bioinformatics* **25**, 2078–2079 (2009). doi:10.1093/bioinformatics/btp352 Medline

71. N. D. Grubaugh, K. Gangavarapu, J. Quick, N. L. Matteson, J. G. De Jesus, B. J. Main, A. L. Tan, L. M. Paul, D. E. Brackney, S. Grewal, N. Gurfield, K. K. A. Van Rompay, S. Isern, S. F. Michael, L. L. Coffey, N. J. Loman, K. G. Andersen, An amplicon-based sequencing framework for accurately measuring intrahost virus diversity using PrimalSeq and iVar. *Genome Biol.* **20**, 8 (2019). doi:10.1186/s13059-018-1618-7 Medline

72. *gofasta* (Github, 2022); https://github.com/virus-evolution/gofasta.

73. G. Dudas, *baltic: baltic - backronymed adaptable lightweight tree import code for molecular phylogeny manipulation, analysis and visualisation* (Github, 2021); https://github.com/evogytis/baltic.

74. S. L. Kosakovsky Pond, D. Posada, M. B. Gravenor, C. H. Woelk, S. D. W. Frost, GARD: A genetic algorithm for recombination detection. *Bioinformatics* **22**, 3096–3098 (2006). doi:10.1093/bioinformatics/btl474 Medline

75. D. P. Martin, B. Murrell, M. Golden, A. Khoosal, B. Muhire, RDP4: Detection and analysis of recombination patterns in virus genomes. *Virus Evol.* **1**, vev003 (2015). doi:10.1093/ve/vev003 Medline

76. H. M. Lam, O. Ratmann, M. F. Boni, Improved Algorithmic Complexity for the 3SEQ Recombination Detection Algorithm. *Mol. Biol. Evol.* **35**, 247–251 (2018). doi:10.1093/molbev/msx263 Medline

77. M. F. Boni, P. Lemey, X. Jiang, T. T.-Y. Lam, B. W. Perry, T. A. Castoe, A. Rambaut, D. L. Robertson, Evolutionary origins of the SARS-CoV-2 sarbecovirus lineage responsible for the COVID-19 pandemic. *Nat. Microbiol.* **5**, 1408–1417 (2020). doi:10.1038/s41564-020-0771-4 Medline

78. A. Rambaut, T. T. Lam, L. Max Carvalho, O. G. Pybus, Exploring the temporal structure of heterochronous sequences using TempEst (formerly Path-O-Gen). *Virus Evol.* **2**, vew007 (2016). doi:10.1093/ve/vew007 Medline

79. A. Rambaut, A. J. Drummond, D. Xie, G. Baele, M. A. Suchard, Posterior Summarization in Bayesian Phylogenetics Using Tracer 1.7. *Syst. Biol.* **67**, 901–904 (2018). doi:10.1093/sysbio/syy032 Medline

80. F. Li, Y.-Y. Li, M.-J. Liu, L.-Q. Fang, N. E. Dean, G. W. K. Wong, X.-B. Yang, I. Longini, M. E. Halloran, H.-J. Wang, P.-L. Liu, Y.-H. Pang, Y.-Q. Yan, S. Liu, W. Xia, X.-X. Lu, Q. Liu, Y. Yang, S.-Q. Xu, Household transmission of SARS-CoV-2 and risk factors for susceptibility and infectivity in Wuhan: A retrospective observational study. *Lancet Infect. Dis.* **21**, 617–628 (2021). doi:10.1016/S1473-3099(20)30981-6 Medline

81. *EpiNow2: Estimate Realtime Case Counts and Time-varying Epidemiological Parameters* (Github, 2020); https://github.com/epiforecasts/EpiNow2.

82. N. Moshiri, NiemaGraphGen: A memory-efficient global-scale contact network simulation toolkit. *GIGAbyte* 10.46471/gigabyte.37 (2022).

83. A. L. Barabasi, R. Albert, Emergence of scaling in random networks. *Science* **286**, 509–512 (1999). doi:10.1126/science.286.5439.509 Medline

84. S. Eubank, H. Guclu, V. S. Kumar, M. V. Marathe, A. Srinivasan, Z. Toroczkai, N. Wang, Modelling disease outbreaks in realistic urban social networks. *Nature* **429**, 180–184 (2004). doi:10.1038/nature02541 Medline

85. J. Mossong, N. Hens, M. Jit, P. Beutels, K. Auranen, R. Mikolajczyk, M. Massari, S. Salmaso, G. S. Tomba, J. Wallinga, J. Heijne, M. Sadkowska-Todys, M. Rosinska, W. J. Edmunds, Social contacts and mixing patterns relevant to the spread of infectious diseases. *PLOS Med.* **5**, e74 (2008). doi:10.1371/journal.pmed.0050074 Medline

86. F. D. Sahneh, A. Vajdi, H. Shakeri, F. Fan, C. Scoglio, GEMFsim: A stochastic simulator for the generalized epidemic modeling framework. *J. Comput. Sci.* **22**, 36–44 (2017). doi:10.1016/j.jocs.2017.08.014

87. X. Yang, Y. Yu, J. Xu, H. Shu, J. Xia, H. Liu, Y. Wu, L. Zhang, Z. Yu, M. Fang, T. Yu, Y. Wang, S. Pan, X. Zou, S. Yuan, Y. Shang, Clinical course and outcomes of critically ill patients with SARS-CoV-2 pneumonia in Wuhan, China: A single-centered, retrospective, observational study. *Lancet Respir. Med.* **8**, 475–481 (2020). doi:10.1016/S2213-2600(20)30079-5 Medline

88. F. Zhou, T. Yu, R. Du, G. Fan, Y. Liu, Z. Liu, J. Xiang, Y. Wang, B. Song, X. Gu, L. Guan, Y. Wei, H. Li, X. Wu, J. Xu, S. Tu, Y. Zhang, H. Chen, B. Cao, Clinical course and risk factors for mortality of adult inpatients with COVID-19 in Wuhan, China: A retrospective cohort study. *Lancet* **395**, 1054–1062 (2020). doi:10.1016/S0140-6736(20)30566-3 Medline

89. J. Yang, X. Chen, X. Deng, Z. Chen, H. Gong, H. Yan, Q. Wu, H. Shi, S. Lai, M. Ajelli, C. Viboud, P. H. Yu, Disease burden and clinical severity of the first pandemic wave of COVID-19 in Wuhan, China. *Nat. Commun.* **11**, 5411 (2020). doi:10.1038/s41467-020-19238-2 Medline

90. N. Moshiri, TreeSwift: A massively scalable Python tree package. *SoftwareX* **11**, 100436 (2020). doi:10.1016/j.softx.2020.100436

91. J. Ma, First Chinese coronavirus cases may have been infected in October 2019, says new research. *South China Morning Post* (2021); https://www.scmp.com/news/china/science/article/3126499/first-chinese-covid-19-cases-may-have-been-infected-october-2019.

92. K. Andersen, Clock and TMRCA based on 27 genomes. *Virological* (2020); https://virological.org/t/clock-and-tmrca-based-on-27-genomes/347/6.

93. L. Pipes, H. Wang, J. P. Huelsenbeck, R. Nielsen, Assessing Uncertainty in the Rooting of the SARS-CoV-2 Phylogeny. *Mol. Biol. Evol.* **38**, 1537–1543 (2021). doi:10.1093/molbev/msaa316 Medline

94. T. Murata, A. Sakurai, M. Suzuki, S. Komoto, T. Ide, T. Ishihara, Y. Doi, Shedding of Viable Virus in Asymptomatic SARS-CoV-2 Carriers. *MSphere* **6**, e00019-21 (2021). doi:10.1128/mSphere.00019-21 Medline

95. T. Sekizuka, K. Itokawa, T. Kageyama, S. Saito, I. Takayama, H. Asanuma, N. Nao, R. Tanaka, M. Hashino, T. Takahashi, H. Kamiya, T. Yamagishi, K. Kakimoto, M. Suzuki, H. Hasegawa, T. Wakita, M. Kuroda, Haplotype networks of SARS-CoV-2 infections in the *Diamond Princess* cruise ship outbreak. *Proc. Natl. Acad. Sci. U.S.A.* **117**, 20198–20201 (2020). doi:10.1073/pnas.2006824117 Medline

96. Y. Turakhia, B. Thornlow, A. S. Hinrichs, N. De Maio, L. Gozashti, R. Lanfear, D. Haussler, R. Corbett-Detig, Ultrafast Sample placement on Existing tRees (UShER) enables real-time phylogenetics for the SARS-CoV-2 pandemic. *Nat. Genet.* **53**, 809–816 (2021). doi:10.1038/s41588-021-00862-7 Medline

97. P. Zhou, X.-L. Yang, X.-G. Wang, B. Hu, L. Zhang, W. Zhang, H.-R. Si, Y. Zhu, B. Li, C.-L. Huang, H.-D. Chen, J. Chen, Y. Luo, H. Guo, R.-D. Jiang, M.-Q. Liu, Y. Chen, X.-R.

Shen, X. Wang, X.-S. Zheng, K. Zhao, Q.-J. Chen, F. Deng, L.-L. Liu, B. Yan, F.-X. Zhan, Y.-Y. Wang, G.-F. Xiao, Z.-L. Shi, A pneumonia outbreak associated with a new coronavirus of probable bat origin. *Nature* **579**, 270–273 (2020). doi:10.1038/s41586-020-2012-7 Medline

98. M. Ghafari, L. du Plessis, J. Raghwani, S. Bhatt, B. Xu, O. G. Pybus, A. Katzourakis, Purifying selection determines the short-term time dependency of evolutionary rates in SARS-CoV-2 and pH1N1 influenza. *Mol. Biol. Evol.* **39**, msac009 (2022). doi:10.1093/molbev/msac009 Medline

99. S. Duchêne, E. C. Holmes, S. Y. W. Ho, Analyses of evolutionary dynamics in viruses are hindered by a time-dependent bias in rate estimates. *Proc. Biol. Sci.* **281**, 20140732 (2014). doi:10.1098/rspb.2014.0732 Medline

100. J. Dushoff, S. W. Park, Speed and strength of an epidemic intervention. *Proc. Biol. Sci.* **288**, 20201556 (2021). doi:10.1098/rspb.2020.1556 Medline

101. J. T. Wu, K. Leung, M. Bushman, N. Kishore, R. Niehus, P. M. de Salazar, B. J. Cowling, M. Lipsitch, G. M. Leung, Estimating clinical severity of COVID-19 from the transmission dynamics in Wuhan, China. *Nat. Med.* **26**, 506–510 (2020). doi:10.1038/s41591-020-0822-7 Medline

102. C. Huang, Y. Wang, X. Li, L. Ren, J. Zhao, Y. Hu, L. Zhang, G. Fan, J. Xu, X. Gu, Z. Cheng, T. Yu, J. Xia, Y. Wei, W. Wu, X. Xie, W. Yin, H. Li, M. Liu, Y. Xiao, H. Gao, L. Guo, J. Xie, G. Wang, R. Jiang, Z. Gao, Q. Jin, J. Wang, B. Cao, Clinical features of patients infected with 2019 novel coronavirus in Wuhan, China. *Lancet* **395**, 497–506 (2020). doi:10.1016/S0140-6736(20)30183-5 Medline

103. R. Ke, E. Romero-Severson, S. Sanche, N. Hengartner, Estimating the reproductive number R_0 of SARS-CoV-2 in the United States and eight European countries and implications for vaccination. *J. Theor. Biol.* **517**, 110621 (2021). doi:10.1016/j.jtbi.2021.110621 Medline

104. L. Pellis, F. Scarabel, H. B. Stage, C. E. Overton, L. H. K. Chappell, E. Fearon, E. Bennett, K. A. Lythgoe, T. A. House, I. Hall; University of Manchester COVID-19 Modelling Group, Challenges in control of COVID-19: Short doubling time and long delay to effect of interventions. *Philos. Trans. R. Soc. Lond. B Biol. Sci.* **376**, 20200264 (2021). doi:10.1098/rstb.2020.0264 Medline

105. Q. Li, X. Guan, P. Wu, X. Wang, L. Zhou, Y. Tong, R. Ren, K. S. M. Leung, E. H. Y. Lau, J. Y. Wong, X. Xing, N. Xiang, Y. Wu, C. Li, Q. Chen, D. Li, T. Liu, J. Zhao, M. Liu, W. Tu, C. Chen, L. Jin, R. Yang, Q. Wang, S. Zhou, R. Wang, H. Liu, Y. Luo, Y. Liu, G. Shao, H. Li, Z. Tao, Y. Yang, Z. Deng, B. Liu, Z. Ma, Y. Zhang, G. Shi, T. T. Y. Lam, J. T. Wu, G. F. Gao, B. J. Cowling, B. Yang, G. M. Leung, Z. Feng, Early Transmission Dynamics in Wuhan, China, of Novel Coronavirus-Infected Pneumonia. *N. Engl. J. Med.* **382**, 1199–1207 (2020). doi:10.1056/NEJMoa2001316 Medline

106. M. Chinazzi, J. T. Davis, M. Ajelli, C. Gioannini, M. Litvinova, S. Merler, A. Pastore Y Piontti, K. Mu, L. Rossi, K. Sun, C. Viboud, X. Xiong, H. Yu, M. E. Halloran, I. M. Longini Jr., A. Vespignani, The effect of travel restrictions on the spread of the 2019 novel coronavirus (COVID-19) outbreak. *Science* **368**, 395–400 (2020). doi:10.1126/science.aba9757 Medline

107. R. Li, S. Pei, B. Chen, Y. Song, T. Zhang, W. Yang, J. Shaman, Substantial undocumented infection facilitates the rapid dissemination of novel coronavirus (SARS-CoV-2). *Science* **368**, 489–493 (2020). doi:10.1126/science.abb3221 Medline

108. N. Moshiri, CoaTran: Coalescent tree simulation along a transmission network. bioRxiv [Preprint] (2020). https://doi.org/10.1101/2020.11.10.377499.

109. K. M. Braun, G. K. Moreno, C. Wagner, M. A. Accola, W. M. Rehrauer, D. A. Baker, K. Koelle, D. H. O'Connor, T. Bedford, T. C. Friedrich, L. H. Moncla, Acute SARS-CoV-2 infections harbor limited within-host diversity and transmit via tight transmission bottlenecks. *PLOS Pathog.* **17**, e1009849 (2021). doi:10.1371/journal.ppat.1009849 Medline

110. J. Ma, Coronavirus: China's first confirmed Covid-19 case traced back to November 17. *South China Morning Post* (2020); https://www.scmp.com/news/china/society/article/3074991/coronavirus-chinas-first-confirmed-covid-19-case-traced-back.
